# Ribosome Synthesis and MAPK Activity Modulate Ionizing Radiation-Induced Germ Cell Apoptosis in *Caenorhabditis elegans*

**DOI:** 10.1371/journal.pgen.1003943

**Published:** 2013-11-21

**Authors:** Ralf Eberhard, Lilli Stergiou, E. Randal Hofmann, Jen Hofmann, Simon Haenni, Youjin Teo, André Furger, Michael O. Hengartner

**Affiliations:** 1 Institute of Molecular Life Sciences, University of Zurich, Zurich, Switzerland; 2 MLS Graduate School and MD/PhD program, Zurich, Switzerland; 3 Department of Biochemistry, University of Oxford, Oxford, United Kingdom; The University of Texas Health Science Center at Houston, United States of America

## Abstract

Synthesis of ribosomal RNA by RNA polymerase I (RNA pol I) is an elemental biological process and is key for cellular homeostasis. In a forward genetic screen in *C. elegans* designed to identify DNA damage-response factors, we isolated a point mutation of RNA pol I, *rpoa-2(op259)*, that leads to altered rRNA synthesis and a concomitant resistance to ionizing radiation (IR)-induced germ cell apoptosis. This weak apoptotic IR response could be phenocopied when interfering with other factors of ribosome synthesis. Surprisingly, despite their resistance to DNA damage, *rpoa-2(op259)* mutants present a normal CEP-1/p53 response to IR and increased basal CEP-1 activity under normal growth conditions. In parallel, *rpoa-2(op259)* leads to reduced Ras/MAPK pathway activity, which is required for germ cell progression and physiological germ cell death. Ras/MAPK gain-of-function conditions could rescue the IR response defect in *rpoa-2(op259)*, pointing to a function for Ras/MAPK in modulating DNA damage-induced apoptosis downstream of CEP-1. Our data demonstrate that a single point mutation in an RNA pol I subunit can interfere with multiple key signalling pathways. Ribosome synthesis and growth-factor signalling are perturbed in many cancer cells; such an interplay between basic cellular processes and signalling might be critical for how tumours evolve or respond to treatment.

## Author Summary

Maintenance of genome integrity and tissue homeostasis is critically important for organisms to survive. For adequate responses to DNA damage or other types of stress, cells have evolved intricate surveillance mechanisms such as repair, cell cycle arrest and apoptosis. Disruption of these response pathways leads to an enhanced probability of cancer. The nematode *C. elegans* has proven a valuable model to study the genetic basis of apoptosis as an adaptive response to genotoxic or cytotoxic insults. Here, we have used *C. elegans* in a non-biased genetic screen to identify further regulators of DNA damage-induced apoptosis and find that ribosome synthesis factors critically determine cell death levels. We show that the newly isolated mutant *rpoa-2(op259)*, which has a ribosomal RNA synthesis defect, is resistant to apoptosis as a result of concurrent alterations in p53 and Ras/MAP kinase signalling. Changes in ribosome synthesis and altered Ras/MAPK activity can be observed in many tumor types, and perturbed p53 activity is among the most frequently detected abnormities in human cancer. The interaction between ribosome synthesis and major signalling pathways is potentially of high relevance for tumor biology.

## Introduction

Multicellular organisms use genetically determined cell death mechanisms – most prominently apoptosis – to ensure the timely and innocuous removal of superfluous, damaged, or potentially harmful cells. Apoptosis is tightly regulated and an integral part of the delicate balance between cell proliferation and cell loss, which is essential for the formation and maintenance of tissues and organs. Disturbances leading to excessive or diminished apoptosis contribute to devastating human diseases with increasing prevalence worldwide, such as neurodegeneration, immunological disorders, and cancer.

The integrity of our genome is continuously challenged by endogenous processes (replication errors, reactive oxygen species, by-products of cellular metabolism) and by exogenous genotoxic agents – naturally occurring or iatrogenic [e.g., 1]. Depending on the cell type, cell cycle stage, metabolic state and probably also tissue context, excessive DNA damage can be a strong stimulus for cells to undergo apoptosis. Detailed knowledge of the DNA damage response network and its failures is required for our understanding of tumor formation and progression; and also for effective and safe tumor treatment, as many of the current treatments produce DNA damage to induce replicative arrest or death of rapidly proliferating tumor cells.

Nucleoli have been increasingly acknowledged as pivot systems for homeostatic regulation and stress responses [e.g.], [ 2]–[4]. Hypertrophic and irregularly shaped nucleoli were reported to be characteristic of malignant cells already at the end of the 19^th^ century, and have gained a high prognostic value for several human neoplasias [5]. However, large nucleoli and increased ribosome biogenesis are common signatures of proliferating cells given the increased demand for protein synthesis. It has therefore been challenging to explore whether nucleolar hypertrophy in cancer cells is a mere expression of rapid proliferation, or whether the enlarged nucleoli could play a causative role in tumor development [6]. Synthesis of ribosomal RNA accounts for up to 75% of total transcriptional activity in yeast [7] and at least 50% of the synthetic effort of rapidly proliferating eukaryotic cells are expended on ribosome production [8]. Transcription of rRNA by RNA polymerase I (RNA pol I) is the rate-limiting step for ribosome synthesis [e.g., 9] and a major target of many signalling pathways of cellular growth and proliferation, e.g., Ras/ERK [10], Myc [e.g., 11], mTOR [12], pRb [13], and p53 [14]. In turn, physiological or pathological changes in rRNA transcription and processing, or in nucleolar integrity affect cellular fate decisions through altered translation, but also through more direct regulatory signalling [6], [15], [16]. Various disturbances in early steps of rRNA synthesis have strong pro-apoptotic effects [17]–[19]. Somewhat contrasting, partial depletion of ribosomal proteins in zebrafish proved to be cancerogenic [20]. Also in humans, tumor predisposing diseases could be linked to mutations in rRNA processing and ribosome assembly factors, such as Dyskeratosis congenita (to pseudouridine synthase DKC1) or Diamond-Blackfan anaemia (to various ribosomal proteins) [e.g.], [ 16,21].

The germ line of *Caenorhabditis elegans* has proven a versatile model to dissect the classical DNA damage responses [22] in the context of an optically transparent, living organism [23]. Proliferation arrest of mitotic germ stem cells and apoptosis of meiotic germ cells occur in two spatially separate areas and can be morphologically followed by Nomarski differential interference contrast (DIC) microscopy. Under standard laboratory conditions, germ cells at the exit from late meiotic pachytene stage alternatively progress into large oocytes or undergo apoptosis [24]. This developmental, ‘physiological’ apoptosis affects an estimated half of all oocyte precursors in an apparently stochastic manner, possibly to supply the rapidly growing oocytes with cytoplasmic constituents [reviewed in [25]]. Two regulatory pathways of global importance for cellular growth and proliferation have been associated with physiological cell death: the Rb complex [26], [27] and Ras/MAP kinase signalling [28], [29]. In contrast to physiological apoptosis, DNA damage-induced apoptosis depends on CEP-1 (a functional *C. elegans* homolog of mammalian p53 [30], [31]), which transcriptionally upregulates the two pro-apoptotic BH3-only proteins EGL-1 and CED-13 [32]. In the classical model (schematic in Fig. S13A), EGL-1 activates the core apoptotic machinery by binding to CED-9/Bcl-2, therewith releasing CED-4/Apaf-1 [33], which in turn oligomerises and serves as a platform for activation of the effector caspase CED-3 [reviewed in [34]].

Many of the factors involved in the maintenance of genome integrity are evolutionarily conserved in *C. elegans*. In this work, we characterise the mutation *rpoa-2(op259)* – isolated in an unbiased forward genetic screen – which affects the second largest subunit of RNA polymerase I (RNA pol I) and thus an essential and highly conserved gene. Taking advantage of this viable allele, we identified causal links between ribosome synthesis and cellular checkpoints in the context of a whole organism. We show that *rpoa-2(op259)* leads to stimulation of stress signalling and notably to chronic activation of the pro-apoptotic CEP-1/p53 pathway. It simultaneously interferes with Ras/MAPK signalling, possibly by increasing LIP-1 phosphatase activity, and thereby modulates apoptosis downstream of CEP-1, likely at the level of CED-9. Only the combination of these two effects explains the apoptotic phenotype of the RNA pol I mutant under normal conditions and in response to irradiation.

## Results

### Genetic screen for DNA damage response genes yields an RNA polymerase I subunit

**Figure 1:**
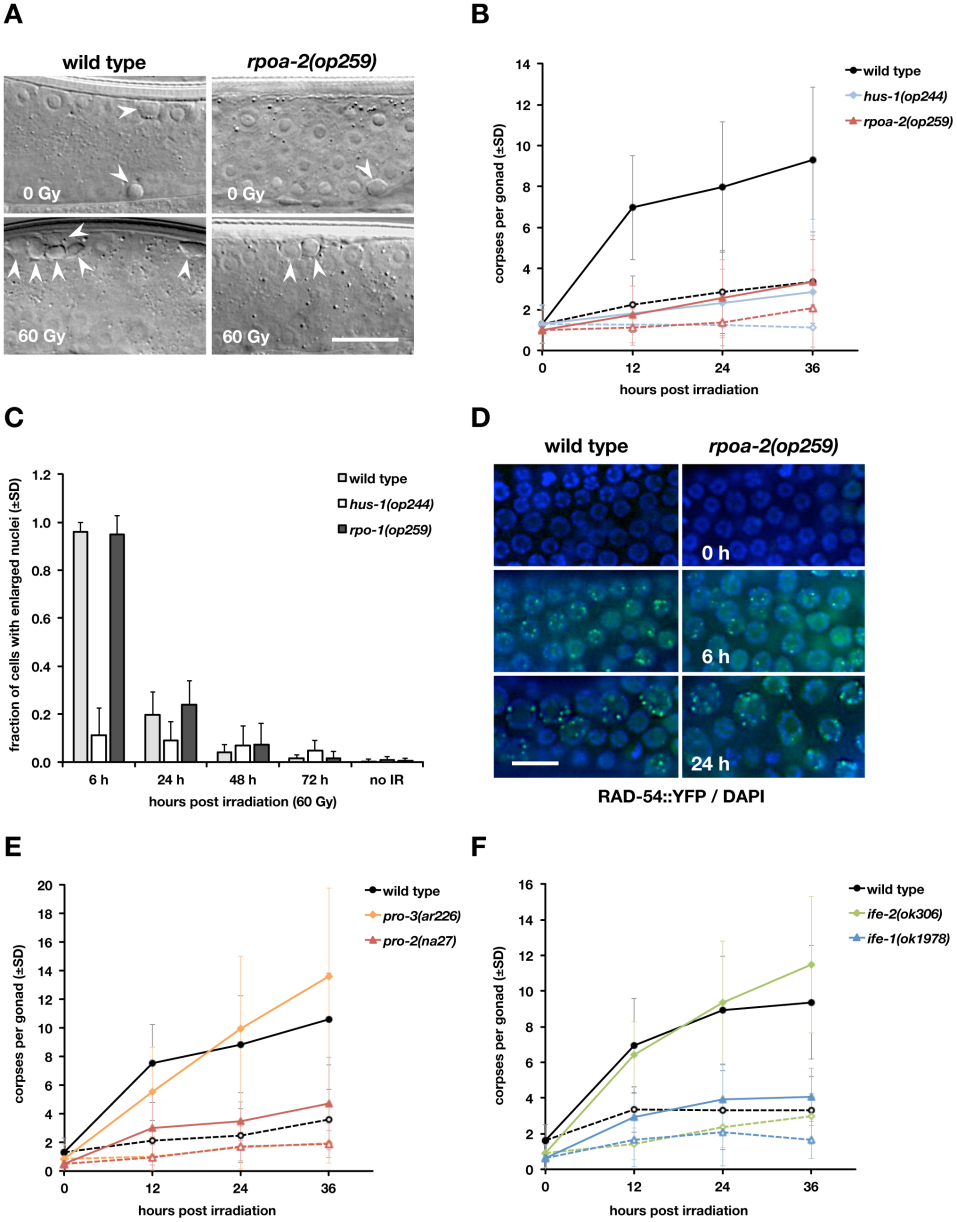
The *rpoa-2(op259)* mutation reduces irradiation-induced germ cell apoptosis but not cell cycle arrest response or DNA damage repair. A) DIC images of adult worms 24 hours after irradiation. Basal (physiologic) as well as DNA damage-induced germ cell death is confined to the late meiotic pachytene region of the germ line tubes; corpses are visible as cellularised, refractile discs (arrowheads). Size bar, 15 µm. B) Apoptotic cell corpses in the germ lines of X-ray treated adult hermaphrodites. Dashed lines represent basal levels (0 Gy), straight lines the levels following irradiation (60 Gy). At least three independent experiments (with n = 20 animals per condition) were performed with wild-type and *rpoa-2(op259)* worms, and one with animals mutant for the 9-1-1 complex subunit HUS-1. Error bars, standard deviation (SD) of corpse number per gonad over all experiments. C, D) In contrast to the DNA damage response mutant *hus-1(op241)*, cell cycle arrest is induced normally in *rpoa-2(op259)*. C) Percentage of mitotic cells with nuclear enlargement following IR. Error bars, SD from at least 12 gonads (approx. 40–50 total mitotic cells per gonad). D) Gonads were dissected from *opIs257[P_rad-54_::rad-54::yfp 3′UTR; unc-119(+)]; unc-119(ed3)* or *rpoa-2(op259)*; *opIs257; unc-119(ed3)* adults, stained with Hoechst, and scored for YFP::RAD-54 foci. Size bar, 10 µm. E, F) A subset of rRNA processing and translation initiation mutants have reduced IR-induced apoptosis. Apoptotic response to IR irradiation in the gonads of the rRNA processing mutants *pro-2(na27)* and *pro-3(ar226)* (E) and of the eIF4E homolog mutants *ife-1(ok1978)* and *ife-2(ok306)* (F). Dashed lines, 0 Gy, straight lines, 60 Gy; error bars, SD of the number of germ cell corpses per gonad over at least 3 experiments (n = 20 animals per data point and experiment).

We searched for additional regulators of cellular DNA damage responses with a forward genetic (EMS mutagenesis) screen (see Methods). Of 2'000 haploid genomes tested, one candidate mutation, *op259*, led to reduced apoptosis of meiotic germ cells following ionising radiation (IR) (Fig. 1A). In *op259*, the basal levels of germ cell corpses were lower than wild type, and IR-induced apoptosis was strongly reduced (Fig. 1B). *op259* mutants also showed a reduced apoptotic response to ultraviolet light (UV-C) (Fig. S1A), speaking for a more global impairment of damage-induced apoptosis.

Genetic mapping and molecular characterisation of *op259* mutants (see Methods) led to the identification of a C→T transition in the second exon of F14B4.3 (Fig. S2A), resulting in a Proline to Serine change (P70S). F14B4.3 codes for the second-largest subunit of eukaryotic RNA polymerase I; we hence named the gene *rpoa-2*.

Several lines of evidence suggest that this missense mutation is the cause of the apoptotic defect in *op259* mutants. First, we tried to phenocopy *rpoa-2(op259)* by knockdown of F14B4.3 using RNAi by feeding [35], [36]. Whereas worms grown on control RNAi conditions showed a strong increase of the cell corpse number upon irradiation, F14B4.3*(RNAi)*-treated animals had only few germ cell corpses (Fig. S1C). Second, transgenic expression of wild-type RPOA-2 could mostly rescue the apoptotic phenotype of *rpoa-2(op259)* (Fig. S1B). Third, *rpoa-2(op259)* failed to complement the independent deletion allele *rpoa-2(ok1970)* available from a public source [37]. Because this allele is homozygous lethal, we generated transheterozygous *rpoa-2(ok1970/op259)* animals by crossing *rpoa-2(op259)* males with *rpoa-2(ok1970)*/*hT2* hermaphrodites. *rpoa-2(ok1970/op259)* were viable and shared the apoptotic phenotype of *rpoa-2(op259)* homozygotes (Text S1 and Fig. S1E). Taken together, our observations confirm that *rpoa-2(op259)* is the cause of the observed apoptotic defects.

The core subunits of the DNA-dependent RNA polymerases (Table S1) are evolutionarily highly conserved [38], particularly the catalytic (largest and second-largest) subunits [39]. Alignment of the RPOA-2 protein sequence with its homologs reveals that the P70S mutation affects a highly conserved residue at the beginning of a well-conserved stretch of amino acids both when comparing diverse orthologous eukaryotic RNA pol I β-subunits (Fig. S2B) as well as the three paralogous *C. elegans* β-subunits of RNA pol I, II, and III (Fig. S2C). In the crystal structure of the RNA pol II core complex from yeast ([40], PDB entry 1I50), the proline corresponding to the residue mutated in RPOA-2 maps to a region that lies distant to the catalytic centre of the polymerase, towards an outer surface of the core complex (not shown), suggesting that the synthesis of RNA is preserved and possibly explaining why the *op259* mutants, unlike the *ok1970* null animals, are viable.

Consistent with the expected function of RPOA-2, we found that a YFP::RPOA-2 transgene under the control of its endogenous promoter is ubiquitously expressed, and localises mainly to the nucleolus (Fig. S3 and Fig. S8B). To test for potential effects of the *op259* mutation on RPOA-2 expression and localisation, we compared YFP-tagged mutant and wild-type protein. In spite of variable expression levels between different transgenic strains, YFP::RPOA-2(P70S) consistently showed stronger relative cytoplasmic fluorescence than YFP::RPOA-2(wt) (Fig. S3).

In conclusion, we found that *op259* is a viable hypomorphic mutation in the essential *rpoa-2* gene and thus offers a valuable means to investigate disturbances in the process of ribosome synthesis in a living system.

### Cell cycle arrest and DNA repair are normal in *rpoa-2(op259)* mutants

Many *C. elegans* DNA damage response mutants show not only defective apoptosis but also defects in DNA repair and cell cycle arrest of proliferating germ cells [23], [41]. In wild-type worms, DNA damage induces cell cycle arrest of mitotic germ cells until the damage has been repaired [42]. Nuclear growth and cytoplasmic expansion meanwhile persist, resulting in a visible enlargement of the germ cell nuclei [43]. Chromatin staining of isolated gonads from control animals showed a uniform pool of nuclei in the mitotic compartment, which was interspersed with larger nuclei shortly after irradiation; at 6 hours, almost all nuclei were considerably enlarged (Fig. 1C). The nuclei in *rpoa-2(op259)* gonads showed a similar response (while the nucleoli grew only little), confirming that germ cells in the mutant do arrest following IR.

To assess DNA repair in *rpoa-2(op259)* germ lines, we used the fluorescent reporter RAD-54::YFP expressed from the transgene *opIs257* [44]. RAD-54 is involved in homologous recombination at sites of DNA double strand breaks (DSB). In addition to its role in meiotic recombination, RAD-54 is also recruited to repair foci when DNA has been damaged exogenously [44], [45]. We found that meiotic cells in *rpoa-2(op259)* animals had similar numbers of RAD-54::YFP foci both under control conditions (0.3±0.1 vs. 0.4±0.1 (95% CI) foci per nucleus), and following IR (3.7±0.3 vs. 3.4±0.4 foci at 3 hours). In the mitotic region, *rpoa-2(op259)* and wild-type worms alike had very few foci under control conditions; and upon irradiation, the number strongly increased in both strains (Fig. 1D). Twenty-four hours after IR, we noticed a strong association of RAD-54::YFP foci with enlarged cells (Fig. 1D), i.e., cells arrested in the cell cycle, likely due to unrepaired DNA damage. The numbers of RAD-54::YFP foci per large mitotic cell were 6.4±1.8 (95% CI) for wild-type and 6.7±1.0 for *rpoa-2(op259)* gonads. Taken together, our observations indicate that *rpoa-2(op259)* mutants have no gross defect in cell cycle arrest response or DSB-repair.

### Abnormal germ cell proliferation and differentiation at sensitive conditions

*rpoa-2(op259)* mutants develop and reproduce less rapidly than wild-type worms. A full generation cycle at 20°C took at least 24 hours longer in *rpoa-2(op259)* than in the wild type (Fig. S4A). Most significantly delayed was the progression from the L4 stage to the appearance of the first eggs on the plate. Further, the egg-laying rate of *rpoa-2(op259)* animals in young adulthood was reduced in comparison to wild type (Fig. S4B); however, the total number of progeny laid per animal was less divergent (179±32 vs. 200±38, n = 6). *rpoa-2(op259)* mutants had no clear signs of oocyte retention or stacking of embryos that would explain a lower output; we thus considered that germ cell proliferation or maturation might be slowed down and assessed germ line dynamics in early adulthood (Fig. S5A and Text S1). The total number of germ cells indeed increased at a slower rate in *rpoa-2(op259)* mutants (Fig. S5B). However, in contrast to many mutants of germ line proliferation characterised so far [e.g.], [ 46], [47, chapters in 48], the numerical proportions of the distinct germ cell stages – mitotic, transition or mid-late pachytene cells – did not significantly deviate from the wild-type pattern; at 24 hours after onset of egg laying, wild type and *rpoa-2(op259)* gonads showed very similar germ cell populations (174 vs. 201 mitotic, 516 vs. 511 meiotic pachytene cells) (Fig. S5C). Thus, *rpoa-2(op259)* mutants have a reduced germ cell production rate, not so much due to an altered mitotic cell pool but most likely due to slower cell cycles. A lower rate of cells exiting late meiotic pachytene might thus contribute to the lower number of apoptotic cells in *rpoa-2(op259)* mutants. However, reduced proliferation alone is unlikely to explain the lower apoptosis rate and weak IR-response in *rpoa-2(op259)* gonads (see further results).

*rpoa-2(op259)* mutants were smaller than wild type at the L4 stage. The mutants however caught up during adulthood and exceeded the wild-type length by 10% at the third day of adulthood. Older animals also contained significantly more intestinal and interstitial lipids, indicative of metabolic alterations. Knockdown of certain translation initiation factors had been shown to considerably prolong adult lifespan in *C. elegans* [e.g.], [ 49,50]. In contrast, *rpoa-2(op259)* animals had a slightly shortened median survival (Fig. S4C).

Whereas *rpoa-2(op259)* worms were fertile at 15°C or 20°C, animals raised at 25°C were sterile. Most *rpoa-2(op259)* worms had not switched from spermatogenesis to oogenesis by 18 to 24 hours after L4 as wild-type worms would [51], and more than half of all gonads revealed the presence of small cells resembling pre-diakinetic germ cells between the most proximal oocyte and the uterus (Fig. S6A). At later stages, these cell clusters expanded to large masses of innumerable cells (Fig. S6B). DAPI staining of these tumours revealed a chromatin pattern typical for mitotic germ cells (Fig. S6B). A similar proximal proliferation (Pro) phenotype had previously been described by Hubbard and colleagues [52]. Interestingly, the conditions that provoked this Pro phenotype were mutations or RNAi knockdowns of genes involved in rRNA processing [53]. Formation of the proximal tumours in these conditions involves events in early germ line development (as many germ cell proliferation phenotypes do [e.g.], [ 54,55]) and is likely a result of spatiotemporal mismatch between early germ cells and the signalling environment [56]. Similarly, we found in temperature shift experiments that the critical developmental stage that determines tumor formation and fertility in *rpoa-2(op259)* mutants was the L3 larval stage (not shown).

We observed a further distinct germ line differentiation defect in a small fraction of *rpoa-2(op259)* mutant gonads: the ectopic presence of large, apparently mature oocytes in the distal arm, followed proximally by apparently normal early pachytene nuclei (Fig. S7A). The penetrance of this defect, which is described in further detail in the Supplementary Results section (Text S1) as Gogo phenotype, was clearly enhanced by irradiation of mutant animals at adult stage. In contrast to the Pro phenotype, which arose independently of *cep-1* and *ced-3* (not shown), the Gogo phenotype could be suppressed by loss of *cep-1* function (Fig. S7C).

In summary, *rpoa-2(op259)* mutants have a delayed germ line development and a reduced germ cell proliferation rate, and show at least two distinct germ cell maturation disorders under restrictive conditions. One of these, the proximal tumor phenotype, is shared with other mutants of ribosome synthesis.

### *rpoa-2(op259)* mutants have altered ribosome synthesis

**Figure 2:**
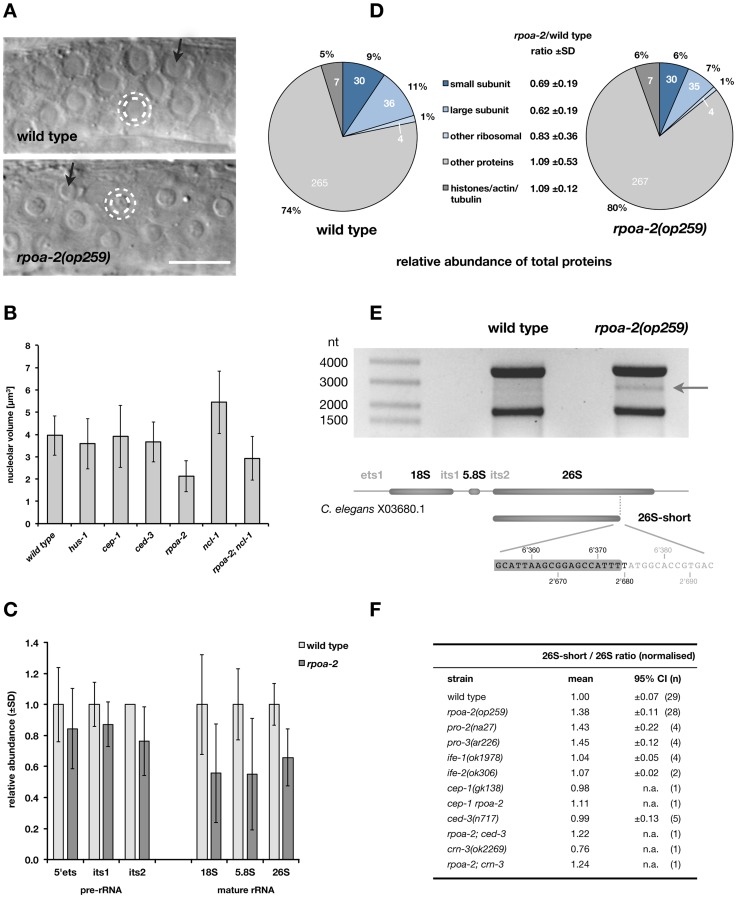
*rpoa-2(op259)* mutants have altered ribosome synthesis. A) Most cells in *C. elegans* exhibit a single but often very prominent nucleolus. In germ cells of the meiotic pachytene stage, the nucleoli (inner white dashed circle) impose as central spheres of approximately half the diameter of the containing nuclei (outer white dashed circle). In *rpoa-2(op259)*, the nucleolar diameter is reduced. Many of the germ cell nucleoli show a substructure by DIC that is enlarged in *rpoa-2(op259)* mutants (black arrows). Size bar, 10 µm. B) Nucleolar volume calculated from measures of the diameter in DIC images of the late meiotic pachytene region of wild-type, *hus-1(op241)*, *cep-1(gk138)*, *ced-3(n717)*, *rpoa-2(op259)*, *ncl-1(e1865)*, and *rpoa-2(op259); ncl-1(e1865)* gonads. Error bars, SD of nucleolar size over all animals tested (12 animals, 5 nucleoli per animal). C) Quantification of ribosomal RNA in whole worm extracts by qRT-PCR. The mean levels of the mature rRNAs (competimer strategy) and of transcribed spacer sequences of the pre-rRNA were normalised by the internal controls in each sample (Pol II transcripts) and compared to N2 wild type. At least 9 or 4 samples per strain were probed for rRNAs or pre-rRNAs, respectively; error bars, 95% CI of the mean. D) Ribosomal proteins represent a smaller fraction of total proteins in *rpoa-2(op259)* mutants compared to wild-type animals. Individual protein abundance was determined by spectral counting and a quantitative value was assigned by the Scaffold 3 software [109]. Ribosomal proteins were grouped as small or large ribosomal subunit proteins, or a small group of acidic ribosomal proteins. A subset of structural proteins is included as a control group. Similar numbers of proteins were identified in each group for both samples (numbers inside circle). Mean ratio of protein abundance in *rpoa-2(op259)* over wild type is indicated. E) Ethidium bromide-stained denaturing agarose gel with 800 ng total RNA from adult wild-type and *rpoa-2(op259)* mutant worms. An approximately 2.8 kb distinct band running between the 26S and 18S ribosomal RNAs is more prominent in *rpoa-2(op259)* (arrow). Characterisation of its sequence and its endings (Text S1 and Fig. S11) revealed a precise truncation of the mature 26S rRNA and no addition of a poly-A tail. The base numbers indicate the position in the reference sequence of the rDNA repeat unit (top) or along the 26S rRNA (bottom), respectively. F) Relative abundance of the 26S-short rRNA in *rpoa-2(op259)*, rRNA processing mutants (*pro-2*, *pro-3*), translation initiation factor mutants (*ife-1*, *ife-2*), apoptosis mutants (*cep-1*, *ced-3*), an exosome mutant (*crn-3*), and of double mutant combinations with *rpoa-2(op259)*. On each northern blot membrane, the ratio of the 26S-short to 26S rRNA signals was arbitrarily set to 1.0 for the wild-type controls. Mean relative ratios, 95% CI of the mean, and number of samples analysed per strain.

Considering that *rpoa-2* codes for the second-largest subunit of RNA pol I, we wished to determine the effect of the *rpoa-2(op259)* mutation on ribosomal RNA synthesis. Germ cell nucleoli in *C. elegans* make up a significant fraction of the nuclear volume and can be readily observed by DIC microscopy. Nucleolar volume was reduced by more than 50% in *rpoa-2(op259)* mutant gonads (Fig. 2B). Additionally, the nucleoli in *rpoa-2(op259)* mutants often exhibited an enlargement of a substructure visible by DIC (Text S1 and Fig. 2A). Similar changes could also be observed in somatic nucleoli (Fig. S8A).

Eukaryotic nucleoli form around the tandem rDNA repeat units, which are transcribed by RNA pol I as one precursor rRNA (pre-rRNA) that is subsequently processed to generate the 18S, 5.8S and 28S rRNA [e.g., 57]. The 18S rRNA together with 33 canonical ribosomal proteins (in yeast) forms the 40S subunit (SSU); the 26S (28S in mammals), 5.8S and the RNA pol III-transcribed 5S rRNAs combine with 46 ribosomal proteins to the 60S subunit (LSU).

Mature ribosomal RNA is thought to constitute about 60% of total cellular RNA in yeast [58]. To quantify such highly abundant RNA by qRT-PCR, we adopted the competimer strategy to the worm (see Methods). We designed competimer primer sets for amplicons of the three RNA pol I transcribed rRNAs (Fig. S11A and Table S4). On average, the relative levels of 18S, 5.8S or 26S in *rpoa-2(op259)* were only about 70% of wild type (Fig. 2C). For the short-lived precursors (pre-rRNA), we used primers that annealed to the transcribed spacers ets, its1 or its2, and would thus amplify the cDNAs of transcripts that had not yet been processed in the respective regions, but not the fully mature rRNAs. Overall, pre-rRNA levels were only slightly reduced in *rpoa-2(op259)* mutants (Fig. 2C). Measurements of *in vivo* rRNA synthesis activity using 5-fluorouridine (5-FU) incorporation also suggested that rRNA transcription is grossly normal in *rpoa-2(op259)* mutants (Text S1 and Fig. S9B).

Eukaryotic pre-rRNA is cleaved at specific sites in a well-defined sequence of exo- and endonucleolytic events [e.g., 59], and more than 100 nucleotides are modified in human rRNA, to yield the mature ribosomal RNAs. Numerous non-ribosomal factors and small nucleolar RNPs are required for this processing and for the assembly and nuclear export of ribosomal subunits [e.g.], [ 60,61]. Defects of certain early steps of eukaryotic rRNA processing can lead to accumulation of pre-rRNA or to processing by alternative routes, eventually resulting in a characteristic pattern of processing intermediates that can be separated by electrophoresis. For instance, *pro-1* mutants in *C. elegans* differently process rRNA at the internal transcribed spacer its2, similar to yeast mutants of its homolog Ipi3 [53].

We examined *rpoa-2(op259)* mutant worms for aberrant rRNA intermediates using digoxigenin (DIG)-labelled antisense RNA probes to various regions of the polycistronic pre-rRNA (ets1, its1, its2; and 18S, 5.8S, 26S rRNAs) (Text S1 and Fig. S11A). We did not detect significant alterations in the levels of early pre-rRNA species or shorter processing intermediates that retained transcribed spacer sequences (not shown). However, we noticed a distinct band between the outstanding 26S and 18S rRNA bands already when separating total RNA in denaturing agarose gels and staining with EtBr (Fig. 2E). This product was more prominent in *rpoa-2(op259)* samples than in wild-type extracts. We found that this molecule corresponded to a truncated version of the 26S rRNA (26S-short), with a processed 3′ end without signs of polyadenylation (Text S1 and Fig. S11D). We assessed the quantitative difference of the 26S-short band between wild type and various mutants from northern blots with probes complementary to the 5′ terminus. The intensity relative to the 26S rRNA was on average 1.4-fold higher in *rpoa-2(op259)* than in wild type (Fig. 2F).

Truncated versions of ribosomal RNA had been described in other species; in mammalian cells, truncated 28S rRNA were observed in the context of apoptosis or of viral infection. We did, however, not find a dependence of the 26S-short band on apoptosis execution (tested with *ced-3(lf)*) or on the multi-exonuclease exosome (tested with *crn-3(lf)*) (Fig. 2F; for details and references, see Text S1). We further looked at the two rRNA processing mutants *pro-2(na27)* (Noc2 in yeast) and *pro-3(ar226)* (Sda1), which share the Pro phenotype with *rpoa-2(op259)*. Similarly to *rpoa-2(op259)*, the 26S-short to 26S rRNA ratio was about 1.4-fold that of wild type for both mutants. The accumulation might thus result from defects in certain steps of rRNA processing, and it supports an effect of *rpoa-2(op259)* on ribosome synthesis beyond transcription of ribosomal RNA.

Quantitative or qualitative changes in transcription and processing of ribosomal RNA likely alter the composition and the activity of ribosomes, and therefore might influence translation. Interfering with translation regulation mechanisms often leads to alterations in protein expression that are confined to a subset of factors rather than being global [e.g.], [ 62,63]. Expression of some genes or groups of genes might thus be differentially affected in *rpoa-2(op259)* – even with a background of largely normal translation. To determine the effect of the *rpoa-2(op259)* mutation on the proteome level, we performed mass spectrometric analysis, applying a label-free quantitation technique on whole worm protein extracts. We could identify a total of 342 proteins in wild-type samples and 343 in *rpoa-2(op259)*; of these, 328 proteins were present in both samples and could be compared quantitatively. Seventy proteins were ribosomal; thus, most of the total 80 ribosomal proteins were represented. Strikingly, in the mutant, most ribosomal proteins were reduced to about 50–70% of wild type when normalised by their fraction of the total in each sample. The average of the normalised ratios between *rpoa-2(op259)* and wild type of individual ribosomal proteins was 0.66 (0.69 for SSU and 0.64 for LSU) (Fig. 2D). Collectively, ribosomal proteins formed 21.8% of total protein in wild type, whereas in *rpoa-2(op259)* they represented a mere 14.2%.

Altogether, we have found that *rpoa-2(op259)* mutants have no significant decrease of ribosomal RNA transcription, but a clear reduction of mature rRNA levels and a similar reduction in the abundance of ribosomal proteins. Cells in *rpoa-2(op259)* animals might therefore have a smaller pool of mature ribosomal subunits and thus a somewhat reduced protein synthesis capacity. In addition, we have characterised an unconventional RNA of relatively high abundance, which has the nucleotide sequence of a 3′-terminally truncated, non-polyadenylated 26S rRNA, and which has higher relative abundance in *rpoa-2(op259)* and in mutants of rRNA processing.

### Impaired rRNA processing reduces irradiation-induced apoptosis

Based on the data above, we considered three alternatives how the *rpoa-2(op259)* mutation might lead to disturbed cell death. First, the apoptotic defects in *rpoa-2(op259)* animals might be a result of globally reduced translation; DNA damage-induced apoptosis is induced through CEP-1-dependent transcriptional upregulation of the BH3 domain protein EGL-1 [30], [64] and therefore clearly requires new protein synthesis. Second, alterations in ribosome biogenesis and the nucleolus might affect germ cell apoptosis through mechanisms that do not necessarily involve protein synthesis. Third, the role of RPOA-2 in apoptosis regulation might be an independent, specific characteristic of this second largest polymerase subunit or even of the mutated site, and might not be directly linked with the overall performance of RNA pol I as a transcription apparatus. To distinguish between these possibilities, we tested the effect of knocking down the expression of other RNA pol I core subunits or associated factors, as well as of other factors involved in ribosome biogenesis or protein synthesis (Table S2 and Table S3). We chose the L3/L4 stage for initiation of RNAi by feeding to minimize detrimental effects on germ line development. Knockdown of RNA polymerase subunits frequently resulted in fertility and growth defects, but did, with the exception of F14B4.3 (*rpoa-2*) and F36A4.7 (*ama-1*) not lead to defective DNA damage-induced apoptosis (Table S2). A possible explanation for the apoptosis defect in *ama-1* (which codes for the largest RNA pol II subunit) is that the reduction of mRNA transcription might become rate-limiting for the rapid transcriptional upregulation of pro-apoptotic BH3-only factors that is required for DNA-damage induced apoptosis or critical for the expression levels of other pro-apoptotic factors.

rRNA processing- and ribosomal assembly factors are critical for the maturation of the small 40S and the large 60S ribosomal subunits. There is a high number of factors involved specifically in the synthesis of either of the two subunits as well as factors needed for both [61]. Interestingly, several worm mutants of rRNA processing factors had been isolated from one genetic screen, based on their conditional proximal proliferation (Pro) phenotype described above [52], [53]. We thus looked for apoptotic defects in the viable Pro mutants *pro-2(na27)* and *pro-3(ar226)*. *pro-2(na27)* animals indeed failed to exhibit increased apoptosis upon IR at the permissive 20°C, similarly to *rpoa-2(op259)* (Fig. 1E). This strengthens the notion that *rpoa-2(op259)* and *pro-2(na27)* have very similar phenotypes and that they likely result in similar molecular defects.

We addressed the question of whether failure in properly processing rRNA might more generally lead to changes in apoptosis. RNAi knockdown of nucleolar proteins, of rRNA processing factors and of representative ribosomal proteins indeed frequently blocked IR-induced apoptosis. However, knockdown of several of these factors entailed strong germ line defects that became visible soon after apoptosis scoring, so that an interpretation is difficult in these cases (Text S1 and Table S3). Nevertheless, these observations support the notion that proper rRNA processing and ribosome synthesis are critical for IR-induced apoptosis of germ cells.

Finally, we looked at the effect of interfering with translation. eIF4E has been recognised as a central factor in translation initiation and is frequently altered in various proliferative diseases [65]. *C. elegans* has five homologs of eIF4E, coded for by the genes *ife-1* to *ife-5* [66]. The five isoforms show specificity for translation of distinct sets of mRNAs [e.g.], [ 67,68]. We tested the major isoforms for somatic translation, *ife-2*, and for germ line translation, *ife-1*. The *ife-1* loss-of-function alleles *ok1978* (Fig. 1F) and *bn127* (data not shown) reduced apoptosis in response to IR, whereas *ife-2(lf)* animals were not obviously defective (Fig. 1F). We also used the bacterial toxin cycloheximide (CHX) to pharmacologically block translation (see Text S1). At 500 µg/ml, most of the IR response was abolished, whereas baseline apoptosis remained unaffected (Fig. S12A). However, this dose also led to massive impairment of animal health and growth. Lower doses that only marginally impaired development had little to no effect on apoptosis (see Text S1).

In summary, we found that interfering with different steps of ribosome synthesis can lead to inhibition of germ cell apoptosis. Chemically interfering with protein translation also inhibits germ cell apoptosis, but only under conditions that are so drastic that they result in widespread defects. By contrast, *rpoa-2(op259)* animals appear largely normal and healthy and with a largely normal proteome profile (excepting ribosomal proteins), suggesting that the *rpoa-2(op259)* mutation is able to reduce apoptotic irradiation response without drastically hampering global translation. Even though we can herewith not exclude that the apoptotic phenotype is due to rate-limiting factors of apoptosis pathways whose expression is critically sensitive to particular alterations of the translation apparatus, the disturbed IR response is likely a more direct effect of abnormal ribosome biogenesis.

### *rpoa-2(op259)* mutants lack physiological germ cell death and show weak apoptotic response to high CEP-1/p53 activity

**Figure 3:**
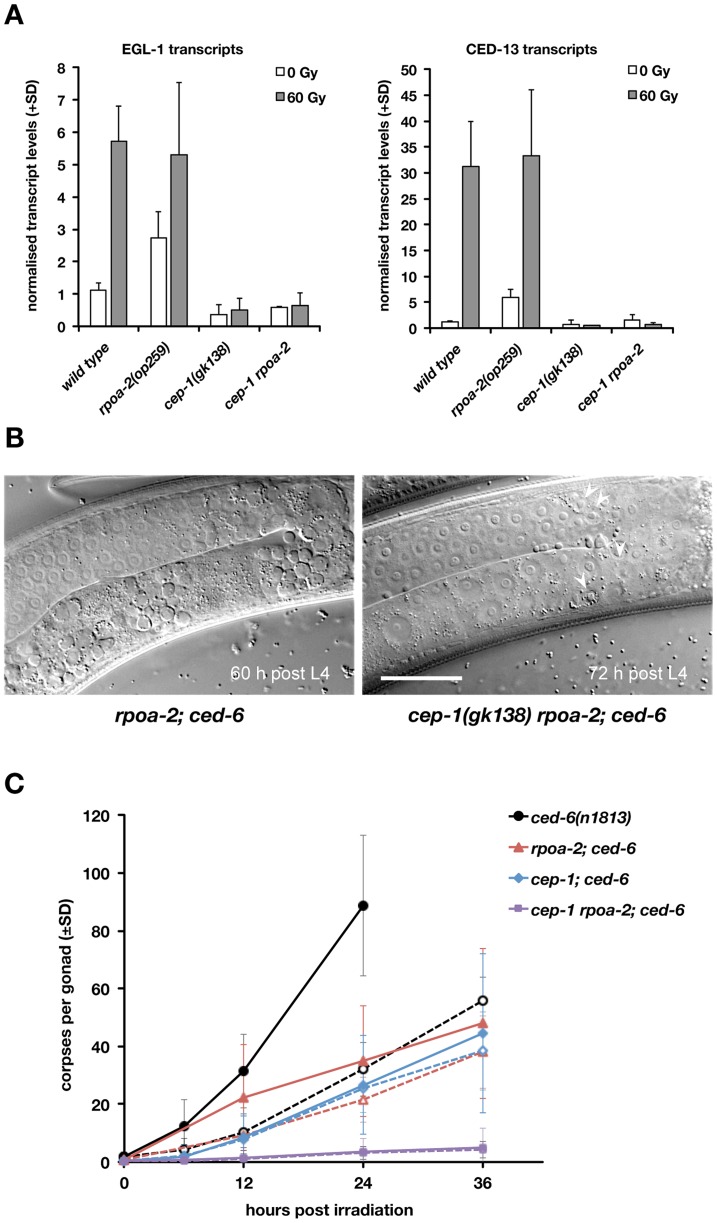
Basal apoptosis in *rpoa-2(op259)* mutant animals depends on CEP-1/p53. A) *rpoa-2(op259)* mutants show increased basal expression of CEP-1 target genes. qRT-PCR measurements of EGL-1 and CED-13 transcript levels in total worm RNA extracts 200 min post treatment. Error bars, SD of normalised transcript levels (wild type and *rpoa-2(op259)*, n>11 independent samples per condition; *cep-1(gk138)* and *cep-1 rpoa-2(op259)*, n = 2). B) Apoptotic corpses (arrowheads) are abundant in *rpoa-2(op259); ced-6(n1813)* double mutants, but rare in *cep-1 rpoa-2; ced-6* triple mutants. Distal gonad arm is at the top, proximal arm with oocytes at the bottom; size bar, 25 µm. C) Germ cell corpse accumulation in the engulfment defective mutant background *ced-6(n1813)*. Apoptotic corpses persist in the gonad instead of being swiftly removed by the surrounding sheath cells, which allows for assessing the cumulative number of cells having undergone apoptosis. Note that an immense number of germ cell corpses are found in irradiated *ced-6(n1813)* animals already at 24 hours after irradiation, but not in *rpoa-2(op259)* or *cep-1(lf)*. Basal levels of apoptosis are observed in *rpoa-2(op259)* and *cep-1* single mutants, but not in the *cep-1 rpoa-2* double mutant. Dashed lines, 0 Gy, straight lines, 60 Gy; error bars, SD over at least 3 experiments (n = 20 animals per data point and experiment).

The data above suggest that *rpoa-2(op259)* might selectively interfere with DNA damage-induced apoptotic signalling. CEP-1 – a functional homolog of mammalian p53 family proteins [30], [69] – is a key player in this pathway, mediating apoptosis mainly by transcriptionally activating the two pro-apoptotic BH3-only proteins EGL-1 and CED-13 [30], [64]. We evaluated the possibility that insufficient activation of CEP-1 is responsible for the weak IR response in *rpoa-2(op259)* by measuring transcript levels of EGL-1 and CED-13 by qRT-PCR. To our surprise, we found that both EGL-1 and CED-13 were efficiently induced in irradiated *rpoa-2(op259)* mutants (Fig. 3A). Moreover, the levels in non-irradiated *rpoa-2(op259)* control animals were much higher than in the wild type. Increased basal levels of EGL-1 and CED-13 were CEP-1-dependent, as they were abrogated in *cep-1(gk138) rpoa-2(op259)* double mutants (Fig. 3A). These observations suggest that *rpoa-2(op259)* mutant animals suffer from a chronic activation of CEP-1/p53, while at the same time being surprisingly resistant to this activation. Consistent with the hypothesis that *rpoa-2(op259)* mutants are resistant to CEP-1-dependent apoptosis, we found that *rpoa-2(op259)* could suppress the strongly increased baseline levels of germ cell apoptosis in the dsDNA break repair mutant *rad-51(lg8701)* (Fig. S13C) as well as the hypersensitivity to irradiation-induced apoptosis in in the Abl kinase mutant *abl-1(ok171)* (Fig. S13B), both of which involve CEP-1 [70].

Intriguingly, *cep-1(gk138) rpoa-2(op259)* double mutants had extremely few corpses, far less than either *cep-1(gk138)* or *rpoa-2(op259)* alone. We used the engulfment defective background *ced-6(n1813)* for better numerical ‘resolution’ of germ cell corpses around the low baseline levels and could confirm these findings (Fig. 3B and Fig. 3C). Thus, loss of *cep-1* function not only suppresses the remaining moderate levels of IR-induced death in *rpoa-2(op259)* gonads, but also eliminates baseline cell death. This result is surprising in so far that most of the basal ‘physiological’ apoptosis observed in adult gonads normally is CEP-1-independent [30]. A hypothesis consistent with all the above information would be that *rpoa-2(op259)* mutants show a reduced sensitivity not only to DNA damage-induced apoptosis, but also to physiological germ cell death cues. The basal apoptosis observed in non-irradiated *rpoa-2(op259)* could then be explained by the increased CEP-1 activity observed in these mutants.

Considering this broader defect in germ cell apoptosis downstream of CEP-1-induced EGL-1 transcription, the *rpoa-2(op259)* mutation ought to influence cell death at the level or downstream of the core apoptotic machinery. In experiments that we describe in detail in the Supplementary Results (see Text S1) we observed that *rpoa-2(op259)* had no influence on the transcript levels of the core factors CED-4 and CED-3 and no obvious effect on the CED-4 expression pattern in pachytene stage germ cells (Fig. S14C). By contrast, *rpoa-2(op259)* showed a strong genetic interaction with *ced-9(RNAi)* or with the hypomorphic *ced-9(n1653)* mutation (Fig. S15C and Fig. S15A). The combination *rpoa-2(op259); ced-9(n1653)* led to highly excessive apoptosis, which could be suppressed by *egl-1(lf)* (Fig. S15B). These findings suggest that *rpoa-2(op259)* impacts the apoptotic machinery at the level of CED-9.

### *rpoa-2(op259)* mutants have low germ line levels of activated MPK-1

MAPK signalling pathways are central regulators of cell proliferation and growth. In the *C. elegans* gonad, Ras/MAPK is required for ‘physiological’ germ cell death and has recently also been shown to modulate DNA damage-induced apoptosis [71]. In addition to the lack of physiological apoptosis, *rpoa-2(op259)* mutants showed a shift of the appearance of the first large oocyte to the proximal arm of the gonad, another germ line feature reminiscent of reduced Ras/MAP kinase activity. This suggested that the loss of CEP-1-independent baseline germ cell death in *rpoa-2(op259)* could be due to reduced Ras/MAPK pathway activity. Hypothetically, such a reduction might also contribute to the weak irradiation response in *rpoa-2(op259)*.

**Figure 4:**
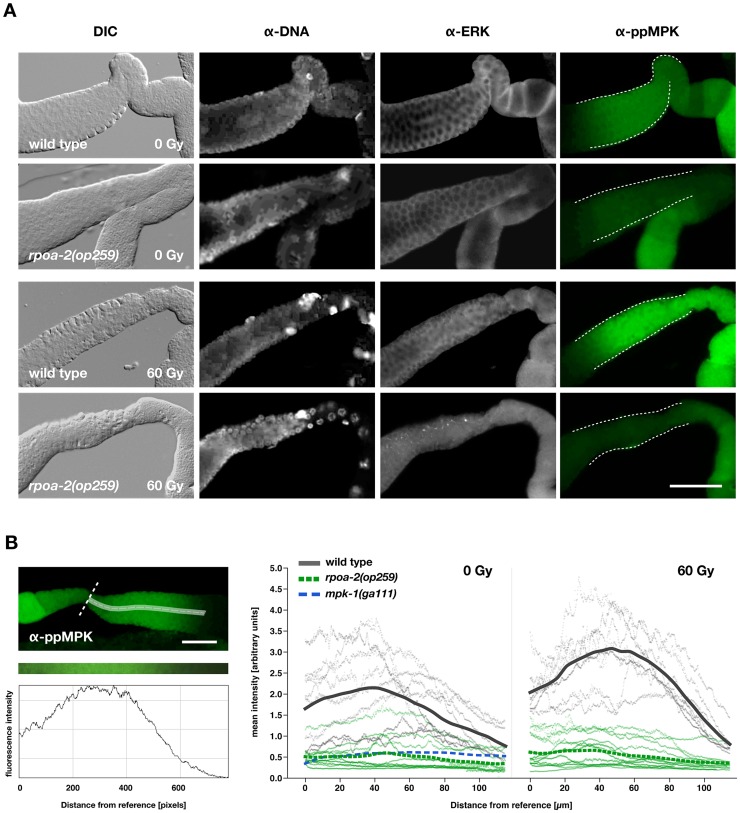
Baseline levels of activated MPK-1 in the gonad are reduced in *rpoa-2(op259)* and do not increase upon irradiation. A) Gonads were extruded from non-irradiated (0 Gy) and irradiated (60 Gy) adult worms 4 hours after treatment, fixed, and immunostained for the doubly phosphorylated (activated) MPK-1. MPK-1 activity is high in the late meiotic pachytene region (white dashed lines) and is further increased following IR in wild-type worms, but not in *rpoa-2(op259)* mutants. Control staining included total MPK-1 with anti-ERK, and anti-dsDNA to detect an epitope other than protein for normalisation of fluorescence intensity. Size bar, 40 µm. B) Quantification of the anti-ppMPK signal intensity along the central axis (white streak) of the late meiotic pachytene region, starting distally to the first oocyte (white dashed reference line) and extending into the mid-pachytene region. Profiles were plotted for at least 8 worms per condition (thin grey lines for individual wild-type worms, thin green lines for individual *rpoa-2(op259)* mutants) and a spline was calculated from their overlay. Size bar, 25 µm.

To measure the levels of activated MPK-1 *in situ*, we performed immunofluorescence analysis on dissected gonads. MAPK-YT, an antibody to di-phosphorylated ERK with cross-reactivity to activated MPK-1 [72] detects high ppMPK-1 levels in the most proximal oocytes and distinct accumulation in the region of late pachytene cells, whereas the distal gonad is generally devoid of signal [73]. We co-stained dissected gonads for ppMPK-1; for total MPK-1; and for dsDNA. For wild-type worms, we observed the signal pattern described above (Fig. 4A). By contrast, in *rpoa-2(op259)* gonads, fluorescence intensity of activated MPK-1 was clearly reduced in the late pachytene region, where germ cells start maturing into oocytes or become apoptotic (Fig. 4A and Fig. 4B). These findings support that the low levels of germ cell apoptosis in *rpoa-2(op259)* mutants could be due to reduced Ras/MAPK activity.

### Overactivation of Ras/MAPK renders germ cells hypersensitive to irradiation-induced apoptosis and restores IR response in *rpoa-2(op259)* mutants

**Figure 5:**
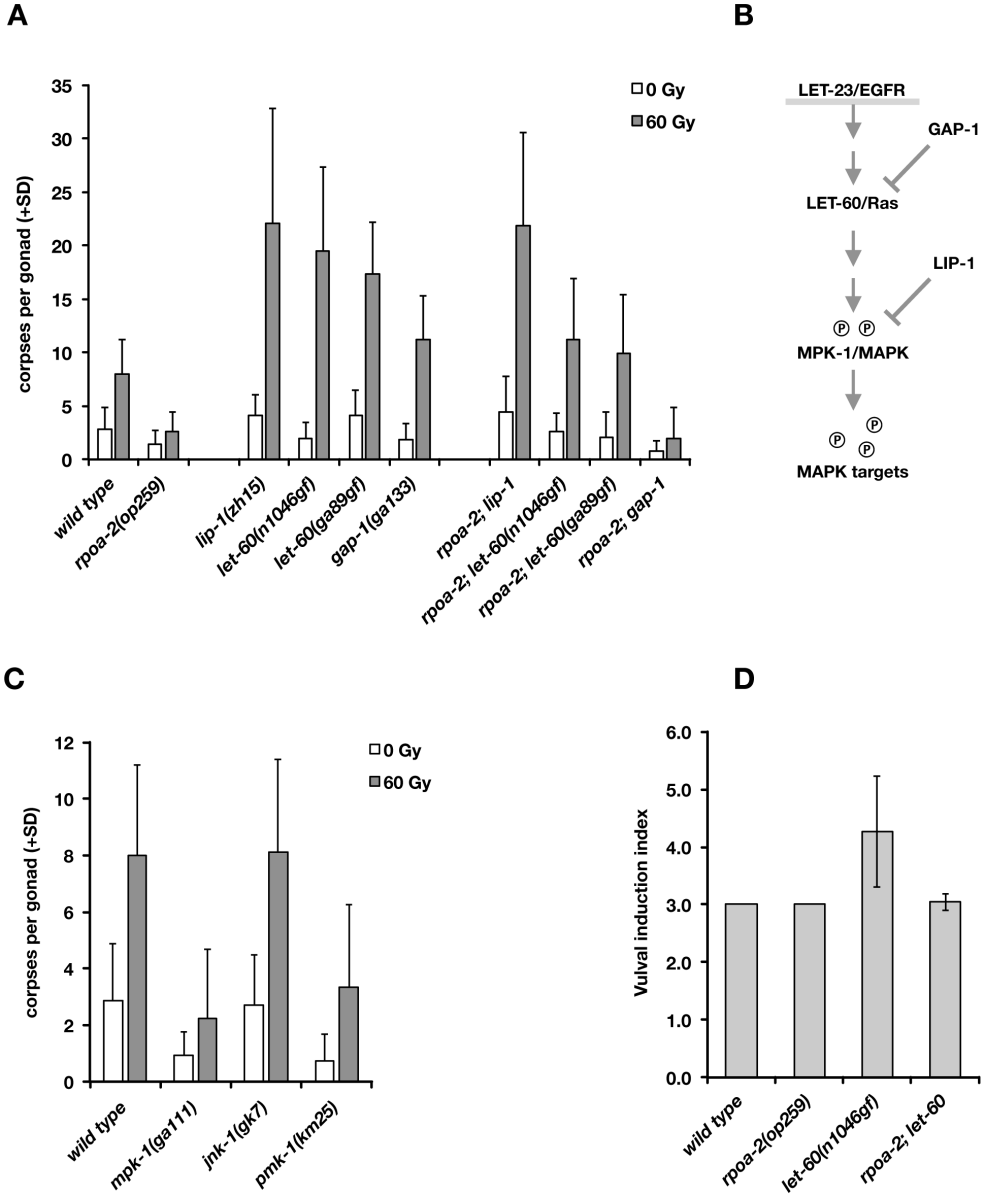
Activated Ras/MAPK pathway enhances the apoptotic DNA damage response and restores irradiation-induced germ cell death in *rpoa-2(op259)* mutants. A) Apoptotic germ cell corpses at 24 hours post irradiation. *lip-1(lf)* and *let-60(gf)* increase the IR response of *rpoa-2(op259)* mutants. *lip-1* is epistatic to *rpoa-2(op259)* regarding the IR response; the response in *rpoa-2(op259); let-60(gf)* is intermediate between the single mutants. Error bars, SD; n≥40 for each condition. B) Simplified model of the EGFR/Ras/MAPK signal axis. Gain-of-function mutations in LET-60 or loss of the phosphatase LIP-1 increase MPK-1 activity. GAP-1 is a GTPase-activating protein acting as a negative regulator of LET-60; *gap-1(lf)* mutants show synthetic effects on vulval induction with other genes but have no strong phenotype on their own. C) Reduced kinase activity in the Ras/MAPK pathway mutant *mpk-1(ga111)* or in the p38 MAPK mutant *pmk-1(km25)* but not the Jnk mutant *jnk-1(gk7)* reduces germ cell apoptosis. Experiments performed at 20°C; error bars, SD; n = 20 animals per condition. D) *rpoa-2(op259)* suppresses excessive vulval induction of *let-60(n1046gf)*. See Methods for assessment of vulval induction index (VI) (reference value is 3.0 for wild type, corresponding to normal induction of one vulva). Error bars, SD of at least 20 animals.

The EGFR/Ras/ERK signalling cascade has been studied in much detail in the context of vulval development in *C. elegans* [e.g., 74]. Briefly (Fig. 5B), binding of the LIN-3 (EGF) signal to LET-23 (EGFR) and activation of the receptor tyrosine kinase activity leads to activation of the Ras GTPase homolog LET-60, and via a kinase cascade, to phosphorylation of the ERK homolog MPK-1. The phosphatase LIP-1 antagonises MAPK activity through dephosphorylation of MPK-1. LIP-1 is also an important regulator of germ cell proliferation [75], oocyte maturation [76], and germ cell apoptosis [77].

We genetically tested whether an increase in Ras/MAPK pathway activity would compensate for the abolished ‘physiological’ cell death in *rpoa-2(op259)* and what the effect would be on irradiation-induced germ cell apoptosis. We generated double mutants between *rpoa-2(op259)* and the *let-60* gain-of-function alleles *let-60(n1046gf)* and *let-60(ga89)*, which both lead to constitutive moderate activation of Ras GTPase activity [77]. In both strains, germ line anatomy was largely normal at 20°C; yet, the cell corpse number upon irradiation rose significantly higher than in wild type (Fig. 5A). Thus, both *let-60(gf)* alleles can at least partially compensate for the apoptotic defect of *rpoa-2(op259)*.

The germ lines of *lip-1(zh15)* loss-of-function mutants had slightly increased baseline apoptosis and were strongly hypersensitive to IR (Fig. 5A), consistent with a recent report [71]. *rpoa-2(op259); lip-1(zh15)* double mutant worms also had higher than wild-type levels of germ cell death at baseline and were equally hypersensitive to IR as *lip-1(zh15)* animals, indicating that *lip-1(zh15)* is fully epistatic to *rpoa-2(op259)* in terms of IR-induced germ cell apoptosis. To evaluate the possibility that the *rpoa-2(op259)* mutation affected LIP-1, we performed anti-LIP-1 immunostaining [75] on extruded gonads of adult animals (see Text S1): the wild-type pattern of membrane-association puncta was lost in the late pachytene region of *rpoa-2(op259)*, leaving a relatively higher cytoplasmic signal (Fig. S17B). We also looked at the somatic expression pattern of a *P_lip-1_::GFP* transcriptional reporter [78] in late larval stages, and found a strongly increased signal in hypodermal cells (Fig. S17A). The genetic and expression data support that *rpoa-2(op259)* might reduce MAPK activity by activating the phosphatase LIP-1. The observation of antagonistic effects of *rpoa-2(op259)* and *let-60(gf)* on germ cell apoptosis levels would fit a model where *rpoa-2(op259)* stimulates phosphatase activity and counteracts phosphorylation of MAPK resulting from constitutive MAPK-kinase activation by *let-60(gf)*.

We and others previously described mutations that lead to increased baseline levels of germ cell death [79]. *gla-3* mutants, which show high levels of CEP-1-independent apoptosis, have increased Ras/MAPK activity both in vulval development and in the germ line [80]. We tested whether reduced *gla-3* would also render germ cells hypersensitive to IR. Due to very close linkage of *gla-3*, *cep-1*, and *rpoa-2*, we used *gla-3(RNAi)* instead of a mutation. Germ cell death was increased to about 25 corpses per gonad in *gla-3(RNAi)* treated worms; upon irradiation, this number rose massively, to 50 corpses on average (Fig. S16A). *gla-3(RNAi)* therefore does not only increase baseline apoptosis, but it potently enhances irradiation response. In *rpoa-2(op259)* mutants, *gla-3(RNAi)* increased the corpse number to approximately wild-type levels. Moreover, *gla-3(RNAi)* significantly restored the IR response in *rpoa-2(op259)* mutants (Fig. S16A), similarly to *let-60(gf)*.

We also tested whether *lip-1(zh15)*, *let-60(n1046gf)* or *gla-3(RNAi)* would restore CEP-1-independent – i.e. ‘physiological’ – germ cell apoptosis in *rpoa-2(op259)*. Indeed, all these conditions re-established baseline apoptosis in *cep-1(gk138) rpoa-2(op259)* at levels that were similar to *cep-1(gk138)* (Fig. S16B).

Remarkably, the conditions leading to activation of Ras/MAPK increased the cell corpse number in *rpoa-2(op259)* without a significant effect on the slow (germ line) development of this mutant. Taken together, these observations suggest that *rpoa-2(op259)* animals suffer from reduced Ras/MAPK pathway activity in the germ line, which can account for both the reduced physiological germ cell death and the reduced sensitivity to DNA damage-induced apoptosis in this mutant.

### *rpoa-2(op259)* antagonises *let-60(gf)* effects on vulval development

To determine whether the negative effect of *rpoa-2(op259)* on MAPK signalling extends beyond the germ line, we looked at vulval development, where MPK-1 controls the cell fates of vulval precursor cells. *let-60(gf)* hyperactivates MPK-1 and leads to extra inductions of vulval precursor cells and to multiple vulvae (Muv phenotype) in the adult animal [81]. The excessive vulval inductions caused by *let-60(gf)* were almost completely suppressed in *rpoa-2(op259); let-60(n1046gf)* double mutants (Fig. 5D). To exclude the possibility that *rpoa-2(op259)* acts downstream of *mpk-1* in this model, we tested epistasis with the MPK-1 target LIN-1. In its non-phosphorylated state, LIN-1 acts as a negative regulator of genes that regulate vulva formation [82]; it is inhibited through phosphorylation by MPK-1. Loss of *lin-1* function thus mimics Ras/MAPK overactivity downstream of *mpk-1*. We found that all *rpoa-2(op259); lin-1(n304)* animals had the maximal number of four extra vulvae, like *lin-1(n304)* single mutants (50 out of 50 animals in both strains), indicating that the effect of the *rpoa-2(op259)* mutation is at the level or upstream of *mpk-1*.

### ERK and p38 MAPK activity are required for wild-type apoptosis levels upon IR

Finally, we tested mutants of MPK-1 and two other MAP kinase pathways in *C. elegans*, PMK-1/p38 and JNK-1/Jnk, for apoptotic IR response. The *mpk-1(ga111)* allele is temperature-sensitive; at 25°C, many gonads show the *mpk-1* loss-of-function pachytene-exit defect and brood size is strongly reduced, while at 20°C, germ cell progression into oocytes still occurs and viable progeny are produced. Even at this permissive temperature, *mpk-1(ga111)* animals had lower than wild-type levels of ‘physiological’ germ cell death and showed a reduced response to IR (Fig. 5C). We also observed an apoptotic defect with RNAi knockdown of *mpk-1* (not shown). These results confirm the importance of MPK-1 in DNA damage-induced germ cell death.

The MAP kinase p38 family homolog PMK-1 is involved in the response to danger signals, such as infectious agents or excess transition metals [83], [84]; lack of functional PMK-1 sensitizes *C. elegans* to toxic effects of pathogens [85]. We tested whether this pathway might also play a role in irradiation-induced apoptosis. Indeed, *pmk-1(km25)* animals had reduced numbers of apoptotic germ cell corpses at baseline or following irradiation (Fig. 5C). In contrast, animals with the *jnk-1(gk7)* loss-of-function mutation had normal, if not slightly increased levels of germ cell corpses following IR (Fig. 5C).

As described above, MAPK signalling has a strong impact on irradiation-induced apoptosis. *mpk-1(rf)* and *rpoa-2(op259)* mutants had only weak IR response; conversely, elevating MAPK activation had a potentiating effect on apoptosis levels, most significantly following IR. We wondered whether the role of MAPK pathway activity was solely to facilitate apoptosis, independently of the additional pro-apoptotic cues, or whether the pathway might actually be activated by irradiation responses, and examined the effect of IR three hours after treatment. The ppMPK-1 signal in the late pachytene region was indeed stronger in IR treated wild-type worms than in non-irradiated controls (Fig. 4A), as also recently reported [71]. By contrast, *rpoa-2(op259)* mutants did not significantly respond to irradiation, and ppMPK-1 signal intensity remained lower than in non-irradiated wild-type worms (Fig. 4B).

Taken together, our observations support the hypothesis that *rpoa-2(op259)* animals have low activation of Ras/MAPK, which likely is responsible for the reduced germ cell apoptosis observed in these animals. Ras/MAPK activity is a prerequisite for germ cell death and seems to sensitise cells for further pro-apoptotic signals. Possibly, the early increase in Ras/MAPK pathway activity following IR additionally boosts germ cell death in response to this genotoxic and cytotoxic treatment.

## Discussion

Here, we describe our characterisation of a point mutation in the second largest subunit of *C. elegans* RNA pol I. This mutation results in distinct, partly conditional germ line phenotypes, such as ectopic germ cell proliferation and tumor formation, precocious germ cell maturation, and reduced germ cell apoptosis. Germ cells in *rpoa-2(op259)* mutants fail to activate apoptosis following X-ray irradiation despite a normal CEP-1-induced upregulation of pro-apoptotic EGL-1. Importantly, CEP-1/p53 activity in non-irradiated mutants is higher than in wild-type controls, but without a concomitant increase in baseline germ cell death. We found that this block in apoptosis is most likely due to reduced levels of activated MPK-1, itself possibly a result of altered LIP-1 phosphatase activity. Hyperactivation of Ras/MAPK signalling is sufficient to restore IR-induced germ cell apoptosis levels in *rpoa-2(op259)*. Ras/MAPK has long been recognised as a prerequisite for ‘physiological’ germ cell apoptosis. Our findings, together with two recent reports [71], [86] extend its role to setting the levels of cell death following irradiation. Whereas the first of these studies suggested a modulatory effect of MPK-1 on CEP-1, we find that it regulates apoptosis most likely downstream of CEP-1 activation, possibly at the level of CED-9/Bcl-2.

### Transcription and processing of ribosomal RNA in *rpoa-2(op259)*

The *rpoa-2(op259)* mutation affects a highly conserved residue in one of the two main catalytic subunits of the RNA polymerase. With various assays, we detected no strong reduction of early pre-rRNA transcripts, but a significant drop of mature ribosomal RNAs, and an according reduction of ribosomal proteins to approximately 70% of wild type. Thus, *rpoa-2(op259)* mutant cells likely have a reduced pool of mature ribosomal subunits, either as a cumulative result of steadily subnormal rRNA transcription, or due to a post-transcriptional effect of *rpoa-2(op259)* on rRNA maturation and ribosome assembly. Supporting the latter possibility, transcription of rRNA has been shown to be interdependent and co-regulated with rRNA processing and pre-ribosome assembly, especially of the SSU [87], [88]. Intriguingly, a mutation in yeast Rpa135, the RPOA-2 homolog, also shows clear evidence for such a link [89]. Northern blot analyses of *rpoa-2(op259)* worm RNA extracts revealed an increase of an RNA band which we were able to characterise as a truncated, non-polyadenylated version of the 26S rRNA. Increased 26S-short levels were also present in the rRNA processing mutant *pro-2(na27)*, which shares various phenotypic aspects with *rpoa-2(op259)*, as well as in mutants of *wdr-46* (UTP7 in yeast, involved in small subunit assembly) [90]. Even though the relevance of this molecule remains speculative (see Text S1), its relative increase in *rpoa-2(op259)* indicates altered rRNA processing or turnover and thus an effect of the *rpoa-2(op259)* mutation beyond transcription. Given the localisation of the P70S substitution on the surface of the RPOA-2 protein, it is conceivable that this mutation disrupts the interaction between the RNA pol I core complex and associated proteins required for proper assembly or disassembly of processing factors, e.g., of the SSU processosome.

### Nucleolar integrity and p53 activation

*rpoa-2(op259)* mutants show increased CEP-1/p53 activation in the absence of exogenous DNA damage. Studies over the last two decades clearly place the nucleolus and ribosome biogenesis at the crossroads of cellular metabolism, cell cycle regulation, growth control, cellular stress responses, aging, and cell death [e.g., 91]. Nucleolar disruption has been recognised as a major hallmark of cellular stress; it can result from reduced rRNA synthesis [e.g., 18] and is possibly even recruited as a mechanistical step towards cellular fate in response to stress like DNA damage [2], [15]. A key function is attributed to the regulation of p53 by nucleolar integrity or nucleolar disintegration. Mdm2, the major ubiquitin ligase and negative regulator of p53, is controlled by the nucleolar tumor suppressor protein ARF (p19^ARF^). ARF is released upon nucleolar breakdown, eventually leading to p53 stabilisation [e.g., 4]. The Mdm2–p53 interaction is also target of ribosomal proteins, e.g., RPL11 or RPL26, that are released from the nucleolus following different types of cellular stress [92].

*rpoa-2(op259)* mutant germ cells have smaller than wild-type nucleoli and enlarged nucleolar substructures, indicating altered nucleolar physiology. Unfortunately, the proposed molecular link between nucleolar integrity and p53 stability cannot be easily transferred from mammals to the worm. Whereas p53 is functionally – and less tightly by sequence – conserved in CEP-1 [30], [31], no homolog of Mdm2 has been found in *C. elegans* (it is conceivable that sequence conservation is very low and therefore the homolog has not been identified, or that alternative ubiquitin ligases regulate CEP-1 stability), and there is no obvious *C. elegans* homolog of ARF. Nevertheless, loss of nucleolar proteins was found to increase resistance to pathogenic bacteria, via a mechanism that involves CEP-1 [93]. Thus, whether by the yet to be identified homologs of ARF and Mdm2 or by alternative mechanisms, it is well possible that the nucleolus is involved in stress responses in *C. elegans* and that CEP-1/p53 activation in *rpoa-2(op259)* is a result of disturbed nucleolar function. It remains to be determined whether the CEP-1::GFP-positive substructures that we observed in germ cell nucleoli [see Text S1; similar structures have also been reported in mammalian cells] are relevant for CEP-1 regulation.

### Anti-apoptotic effects of ribosome synthesis defects

**Figure 6:**
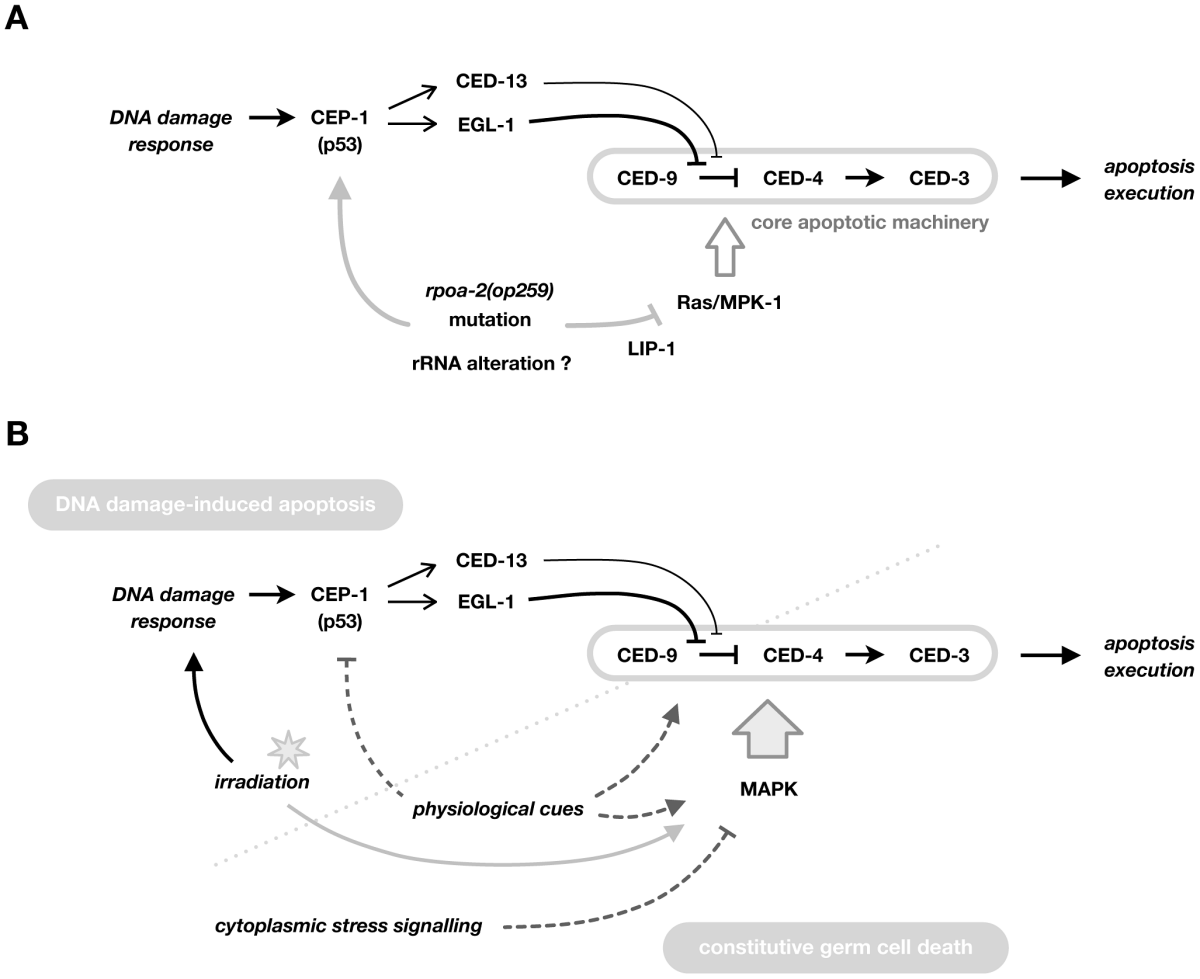
Model for the role of RPOA-2 and Ras/MAPK in determining apoptosis. A) Model for the effect of *rpoa-2(op259)* on the apoptotic network via the Ras/MAPK pathway. CEP-1/p53 activity is induced by irradiation, and increased at baseline in *rpoa-2(op259)* by an unknown mechanism, but this results in only little germ cell apoptosis. *rpoa-2(op259)* thus has an additional inhibitory effect on apoptosis downstream of CEP-1 activation, at the level of the core apoptotic machinery (possibly at the level of CED-9). *rpoa-2(op259)* leads to reduced levels of activated MPK-1 and genetic overactivation of Ras/MAPK restores IR-induced germ cell apoptosis levels in *rpoa-2(op259)* animals, suggesting that this pathway provides the link. *rpoa-2(op259)* might influence Ras/MAPK activity by affecting the phosphatase LIP-1. B) Model for the role of the Ras/MAPK pathway as a central and general modulator of germ cell apoptosis levels. Classically, DNA damage-induced and constitutive ‘physiological’ germ cell death have been distinguished, genetically converging only at the level of the core apoptotic machinery. We and others see that the level of Ras/MAPK pathway activity is decisive for the extent of germ cell death, both at standard growth conditions and following ionising irradiation. Furthermore, activated MPK-1 levels are increased soon after irradiation treatment. For constitutive cell death, it remains to be resolved whether Ras/MAPK pathway activity is simply permissive or whether it is a direct part of the induction signalling. Overall, MAP kinase pathways could be acting as a master lever for the sensitivity of germ cells towards further pro-apoptotic stimuli.

Contrasting with the expected pro-apoptotic effect of increased CEP-1 signalling, germ cell apoptosis levels were not increased in *rpoa-2(op259)*. We show that there is an additional effect of *rpoa-2(op259)* in this *in vivo* system – reduced Ras/MAPK activity – that eventually blocks the pro-apoptotic drive from increased CEP-1/p53 activity, leading to lower than wild-type levels of germ cell death both under normal growth conditions and following DNA damage (Fig. 6A).

How might disturbances in ribosome production lead to reduced Ras/MAPK signalling? *rpoa-2(op259)* counteracts Ras/MAPK activation not only in the germ line, but also e.g., during vulval development. It is possible that disturbed ribosome production, beyond the specific nucleolar signalling described above, leads to a stress response, which might include a reduction in Ras/MAPK signalling. Consistent with this hypothesis, a recent study demonstrated upregulation of pathogen- and xenobiotic-associated defences in worms that had been treated with drugs or RNAi to reduce ribosome synthesis and other essential cellular processes [94]. That study and work in many species have shown that cellular stress leads to changes in the activity of MAPK superfamily members and in their cross-talks [e.g.], [ 95,96], including possibly a reduction in MPK-1/ERK activity.

In our quantitative proteome comparison of *rpoa-2(op259)* and wild type adults, we have found a tendency of the group of proteins annotated with ‘stress response’ towards higher relative abundance in the mutant (Fig. S10). Additionally, we have tested four reporters of stress response genes in the *rpoa-2(op259)* mutant background. Three of them showed increased expression in *rpoa-2(op259)* (Fig. S18). Remarkably, the formerly mentioned study found that upregulation of defence genes was raised globally even if only specific tissues like intestine or hypodermis were targeted with RNAi [94]. Such cross-tissue effects are likely to be in play in *rpoa-2(op259)* as well; altered MAPK activity and germ cell apoptosis need not be a germ line autonomous effect of *rpoa-2(op259)*. Indeed, we found that tissue specific knockdown of *rpoa-2* specifically in the soma reduced germ cell apoptosis as much as knockdown specifically in the germ line (Fig. S1C), suggesting a more global mechanism for the transmission from altered ribosome synthesis to cell death regulation.

It is also conceivable that impaired ribosome synthesis signals starvation-like conditions. Nutritional signalling – particularly the insulin/IGF pathway – is critical for the soma-germ line interaction during germ line development and also for germ cell apoptosis [86], [97], [98]. Additionally, starvation of larval stage worms was shown to counteract the effects of *let-60(gf)* in vulval development [99]. *rpoa-2(op259)* mutants have slower larval growth than wild-type worms and show increased lipid accumulation in adults, indicating significant metabolic alterations. The *rpoa-2(op259)* mutation leads to reduced MPK-1 activity in the gonad and antagonises the effects of LET-60/Ras overactivity on IR-induced germ cell death and on cell fate determination in somatic development. Intriguingly, a very similar phenotypic pattern has recently been reported for the insulin signalling mutants *daf-2* and *pdk-1* [86]. This congruence suggests that an alteration in insulin signalling by *rpoa-2(op259)*, issued in the germ line and/or the soma, could be the link to reduced MAPK activity. Conversely, since mammalian target of rapamycin (mTOR) coordinates ribosome synthesis in response to metabolic state [12], insulin pathway mutants could lead to altered ribosome production and thereby affect MAPK activity and apoptosis.

### Ras/MAPK signalling and germ cell apoptosis

Our genetic analyses of *rpoa-2(op259)* confirm the recently discovered functional relevance for the Ras/MAPK pathway in regulating IR-induced apoptosis [71] and additionally indicate that Ras/MAPK activity modulates germ cell death at a genetic level downstream of CEP-1/p53, likely at the level of CED-9/Bcl-2. Several other mutants beyond *rpoa-2(op259)* are known to show a reduced sensitivity to IR despite functional CEP-1 activation: e.g., the pRb homolog *lin-35* [26], the Sirtuin homolog *sir-2.1* [100], the ceramide synthesis mutant *lagr-1* [101], or the ubiquitin ligase ARF-BP1 homolog *eel-1* [102]. Whether these mutations also lead to reduced MPK-1 signalling remains to be determined. Supporting the model that MAP kinases play a role in stress-induced germ cell apoptosis in parallel to, or downstream of, CEP-1/p53 activation, apoptotic response to excessive arsenite [103] or copper [84] were found to depend on various MAP kinase (ERK/JNK/p38) cascades but not on CEP-1. MAPK pathways are also required for germ cell apoptosis induced by osmotic, oxidative, or heat shock stress [104]. Finally, pathogen-driven germ cell death requires the p38 MAP kinase PMK-1 but not CEP-1 [85].

Altogether, these findings support a model in which CEP-1 and MPK-1 collaboratively establish the level of IR-induced germ cell apoptosis (Fig. 6B). Ras/MAPK activity, beyond facilitating ‘physiological’ germ cell death, is critical for how sensitive cells are towards further pro-apoptotic signals. Thus, MAP kinases might act as “master modulators” of germ cell apoptosis in *C. elegans*.

### *rpoa-2(op259)* – a single mutation at the basis of multiple pathways

Our analysis of *rpoa-2(op259)* mutants demonstrates that a single point mutation affecting a basic cellular process can readily influence multiple key signalling networks, and that the combinatorial effect of these disturbances can lead to quite complex phenotypes. The *rpoa-2(op259)* mutation on the one hand leads to CEP-1/p53 activation, on the other hand reduces MAPK pathway activity (Fig. 6A). Both effects influence germ cell apoptosis, but in opposite directions; reduced Ras/MAPK activity in *rpoa-2(op259)* mutants abolishes ‘physiological’ germ cell death and significantly impairs CEP-1-induced apoptosis; this explains the weak IR-response as well as the reduced baseline germ cell death levels in *rpoa-2(op259)*. At the same time, we find activation of cytoplasmic stress response factors; we can guess from the literature but have no direct evidence from our studies that the activation of these specific stress response pathways leads to the observed reduced levels of activated MAPK. Nevertheless, our observations support the notion that moderate disturbance of an elemental process can lead to very specific changes in signalling rather than an amalgam of generally disrupted cellular functioning. A similar conclusion was recently reached by Melo and Ruvkun, who showed that interfering with basic cellular processes can lead to the specific activation of pathogen-associated response pathways in *C. elegans* [94]. The altered signalling becomes particularly evident when tissues are additionally challenged through exogenous stimuli. Conditional germ line phenotypes in *rpoa-2(op259)* and in other mutants of basic cellular processes are indicative of the delicate new balance in these systems regarding cell proliferation, differentiation, and death.

As the signalling pathways and cellular processes described in this work are all highly conserved through evolution, similar mechanisms might also operate in humans. This has particularly implications for the field of cancer genomics, as it suggests that mutations in genes participating in core cellular processes might, through their modulatory effects on cancer signalling pathways, significantly contribute to the development of at least some types of cancer, and influence how these respond to therapeutic treatment.

## Methods

### Worm strains and culture conditions

Worms were cultured at 20°C (unless indicated otherwise) on NGM agar plates seeded with OP50 strain *E. coli*, according to standard procedures. Synchronisation was reached by bleaching gravid adults. The moment when the first few eggs had been laid by a synchronous worm population was defined as the 0 hour reference time point for adulthood (12 hours post L4 stage in wild-type worms at standard conditions).

Genotypes used in this study (information available on www.wormbase.org):

N2 wild type, *rpoa-2(op259)* (this study); *rpoa-2(ok1970)/hT2*, *pro-2(na27)*, *pro-3(ar226)*, *ife-1(ok1978)*, *ife-1(bn127)*, *ife-2(ok306)*, *ncl-1(e1865)*, *hus-1(op241)*, *cep-1(gk138)*, *ced-6(n1813), ced-3(n717)*, *rad-51(lg8701)*, *abl-1(ok171)*, *let-60(n1046)*, *let-60(ga89)*, *lip-1(zh15)*, *gap-1(ga133)*, *lin-1(n304)*, *mpk-1(ga111)*, *pmk-1(km25)*, *jnk-1(gk7)*, *crn-3(ok2269)*, *rrf-1(pk1417)*, *mut-7(pk204)*, *unc-119(ed3)*.

*opIs257[P_rad-54_::rad-54::yfp; unc-119(+)]*, *opIs219[P_ced-4_::ced-4::gfp; unc-119(+)]*, *opIs198[P_cep-1_::cep-1::gfp; unc-119(+)]*, *zhIs4[P_lip-1_::gfp; unc-119(+)]*, *dvIs19[P_gst-4_::GFP::NLS]*, *frIs7[P_nlp-29_::GFP + P_col-12_::DsRed]*, *muIs84[P_sod-3_::GFP + rol-6]*, *zcIs13[P_hsp-6_::GFP]*, *opIs110[P_lim-7_::act-5::yfp; unc-119(+)]*; *opIs372[P_rpoa-2_::yfp::rpoa-2(+); unc-119(+)]*, *opIs375[P_rpoa-2_::yfp::rpoa-2(op259); unc-119(+)]*, *opIs413[P_hus-1_::yfp::rpoa-2(op259); unc-119(+)]*, *opEx1431[P_hus-1_::yfp::rpoa-2(+); unc-119(+)]*, *opEx1416[P_rpoa-2_rpoa-2(+); unc-119(+)]*.

For the *[rpoa-2]* transgenes, the genomic sequence of *rpoa-2* was fused with optionally an YFP tag and the endogenous promoter- and 3′UTR sequences in the Lazyboy cloning system (primer sequences, see Table S4). Plasmid vectors were introduced into the *C. elegans* germ line by ballistic transformation [105] of *rpoa-2(op259); unc-119(ed3)* animals. The Pro phenotype and temperature sensitive sterility at 25°C could be rescued with *[rpoa-2(+)]* transgenes (Fig. S6C); no rescue was attained, though, with those transgenes that did not show germ line expression.

### RNAi experiments

Gene expression knockdown was performed with RNAi by feeding as described [35], [36]. Briefly, bacteria expressing double-stranded RNA (plasmids were sequenced to confirm the correct target genes) were seeded on NGM agarose plates supplemented with ampicillin and 3 mM IPTG for efficient induction. Worms were synchronised by bleaching; depending on the stage when RNAi had to be initiated, freshly hatched L1 larvae were transferred to the RNAi plates directly, or they were grown on OP50 seeded plates until reaching the appropriate stage and then washed and transferred. As controls, an empty vector RNAi clone and an *unc-22* clone were always included.

### DNA damage response screen

The forward genetic screen was performed as a F1 clonal screen. Because the screen was fairly laborious, we limited our search to 2000 haploid genomes, which is significantly below saturation level. Adult wild-type worms were mutagenized using the chemical alkylating agent ethyl methane sulfonate (EMS, 1.25 mM), 1000 F1 progeny were transferred singly onto new plates, and their offspring were exposed to 60 Gy of X-rays at young adulthood; multiple of these F2 animals were examined for DNA damage response defects using DIC microscopy. One candidate mutation of this screen, *op259*, was mapped with a combination of three-factor and two-factor mapping techniques to the right arm of chromosome I. Fine mapping and gene sequencing in the candidate region revealed a C→T transition in the second exon of F14B4.3. The mutant allele was 6× backcrossed into the wild-type genetic background prior to further characterisation.

### Germ cell apoptosis

Apoptotic germ cell corpses – visible as refractile discs – were scored using Nomarski optics (DIC). Irradiation treatments were performed at the reference time point of adulthood as described above. The number of apoptotic corpses in the late meiotic pachytene region of one of the gonads (anterior or posterior) was counted in 20 worms per condition, either in a time course, or selectively at 24 hours post treatment, when the irradiation-induced corpse number was tending towards a plateau and the germ lines were still without strong signs of aging. For ionizing irradiation (IR), well-fed worms on agar plates were exposed to X-rays in an Isovolt irradiation device for 18.5 min, corresponding to a dose of 60 Gy; for UV-irradiation, worms were treated with short pulses of UV-C in a Stratalinker (254 nm) to reach the indicated energy doses.

### Germ cell arrest and DNA repair foci

Gonads were extruded from adult worms and stained with Hoechst at the indicated time points after irradiation treatment, and images were acquired immediately; the fluorescent images depict slightly more than a simple axial cross-section through the gonads, as these were flattened by the preparation. The total number of mitotic cells and the fraction of enlarged cells were counted according to the chromatin-staining pattern. RAD-54::YFP foci were counted using the ImageJ [106] local intensity peak detection tool with a defined threshold.

### Vulval induction

The six vulval precursor cells (VPCs) are instructed to adopt a certain fate mainly through signalling from the anchor cell (EGFR/Ras/MAPK) and by lateral crosstalk (Notch). Aberrant vulval cell inductions can result in extra vulvae (Muv) or a missing vulva (Vul) in adult animals. Cellular inductions can be accurately assessed with DIC microscopy of L4 larvae [81]. The vulval induction index represents the average number of VPCs that have adopted a 1° or 2° vulval fate (in wild type, VI index is 3.0).

### qRT-PCR experiments

Adult worms were collected once the majority of the population had started laying eggs but no progeny had hatched yet (18 hours post L4 larval stage for wild type; 24 hours later for *rpoa-2(op259)* mutants). Gravid adults were washed from plates, cleared from bacteria and external progeny by settling in M9 buffer 3×5 min, and frozen in liquid nitrogen. Total RNA was extracted using the Nucleospin II kit (Machery and Nagel), and cDNA synthesis performed on 400 ng DNA-free RNA by Superscript III (Invitrogen). qPCR reactions were performed in triplicates, and the mean measured levels for transcripts of interest were normalised to the mean of a set of internal controls, including (in part) PGK-1, RPL-29, RPS-4 and transcripts shown to behave relatively stably between many conditions [107], namely CDC-42, PMP-3, or Y45F10D.4.

#### Competimers

Amplification of highly abundant RNAs can be inhibited by addition of competitive, non-extensible primers to the specific primers of the qPCR reaction [108]. Thus, only a fraction of the highly abundant template is accessible for the polymerase, and amplification efficiency decreases; signal intensity is lowered (Ct value increased) to the range of other (control) transcripts in the same, non-diluted sample. We adapted this method to quantify mature ribosomal RNA in *C. elegans*. Competimer primer sets were designed for amplicons of all the RNA pol I transcribed rRNAs (Table S4), and competitive primers were added to extensible primers in an excess of 4∶1.

### Proteome analysis

Proteins were extracted from well-synchronised adult worms (collected as for RNA extraction, with 2 additional washes in ddH_2_O on ice) using glass beads and Urea/Thiourea (7 M/1 M) buffer and supplemented with 2% CHAPS and 75 mM DTT. Equal amounts of protein were digested with trypsin (100 mM Tris pH 8.5, 1 mM CaCl_2_), and the resulting peptides were – without further fractionation – purified on an MCX cartridge; the high sample volume after elution (5% formic acid/methanol) was reduced by evaporation, and the concentrates were desalted with C18 ZipTips. Peptide concentrations in the final samples were determined to ensure similar loading of the mass spectrometry column. Peptides were measured in triplicate LTQ-FT runs of each non-labelled sample. Estimates of individual protein abundance in the original samples were derived from spectral counting: based on the relative abundance of proteotypic peptides for the proteins identified by a ‘Mascot’ search, a quantitative value was assigned to each protein with the Scaffold 3 software [109].

### MPK-1 immunofluorescence

Antibodies used for immunodetection were: MAPK-YT (Sigma M9692, 1∶100) raised against di-phosphorylated ERK and binding specifically to the homologous ppMPK-1 in *C. elegans* as well [72]; anti-total ERK (1∶200), cross-reacting with MPK-1; a protein-independent control antibody to dsDNA (Abcam HYB331-01; 1∶500) for normalisation. Secondary antibodies: Alexa Fluor goat anti-mouse IgG1 (MAPK-YT), goat anti-rabbit IgG (ERK), goat anti-mouse IgG2a (anti-dsDNA), all 1∶500. Blocking buffer: 10% goat serum in antibody buffer according to [110]. In our staining protocol, gonads were extruded by dissecting adult hermaphrodites 3 hours after irradiation; samples were fixed in 3% PFA for 30 min at 4°C, freeze cracked, fixed in 100% methanol for 10 min at −20°C, stained (blocking for 1 hour at RT; 1° antibodies in blocking buffer o/n at 4°C; 2° antibodies in blocking buffer for 1 hour at RT; >3 washes with PBS/Tween 0.1% between all steps) and mounted on poly-lysine coated slides. To test for specificity of the MPK-1 antibodies, we applied the immunofluorescence protocol to *mpk-1(RNAi)* treated worms. Loss of MPK-1 function was not very severe since most animals showed only minute phenotypes at the stage of analysis. Accordingly, ppMPK-1 and total MPK-1 signals were reduced but not completely absent. Fluorescence pictures were acquired using constant exposure settings and analysed with ImageJ.

## Supporting Information

Figure S1 DNA damage-induced apoptosis in animals with mutations or knockdown of *rpoa-2*. A) *rpoa-2(op259)* mutants fail to induce apoptosis following UV-C irradiation (254 nm). Error bars, SD; n = 20 animals per condition. B) Rescue of the apoptotic phenotype with wild-type *rpoa-2 [rpoa-2(+)]* from the transgene *opEx1416[P_rpoa-2_rpoa-2(+)::rpoa-2 3′UTR; unc-119(+)]*. Error bars, SD of at least 40 gonads. C) Knockdown of *rpoa-2* in the whole animal (wild-type worms), or specifically in the germ line (*rrf-1* mutants) or in the soma (*mut-7* mutants) all affect germ cell apoptosis. *rrf-1(pk1417)*, carrying a mutation in the RNA-directed RNA polymerase RdRP (QDE-1) homolog, selectively abolishes RNAi effects in somatic cells [1], thus exhibiting germ line-specific knockdown, whereas *mut-7(pk204)*, a mutation in the RNaseD homolog, affects the RNAi machinery specifically in the germ line [2] and therefore exhibits soma-specific knockdown. *rpoa-2(RNAi)* was started at L3/L4 larval stage; *ced-9(RNAi)* (started at L1 stage) is included as a control for a germ cell autonomous gene in apoptosis regulation. Error bars, SD; n>15 animals per condition. D) Germ line apoptosis in animals with a balanced deletion of the *rpoa-2* gene at 24 hours after irradiation. Anterior (ant) and posterior (post) gonads were grouped separately due to obviously different levels of germ cell corpses in the balanced *rpoa-2(ok1970)/+* strain. Error bars, SD of at least 40 gonads. E) Germ line apoptosis in transheterozygous *rpoa-2(op259/ok1970)* animals, which were generated by crossing *rpoa-2(op259)* males with *rpoa-2(ok1970)/hT2* hermaphrodites. Error bars, SD of at least 24 gonads.
(PDF)

Figure S2 RPOA-2 is a highly conserved nucleolar protein. A) Overview of the F14B4.3 locus (*rpoa-2* gene); positions of the single base transition in *rpoa-2(op259)* and the deletion of a 1.2 kb genomic fragment in *ok1970* are shown. B) Sequence alignment of eukaryotic RNA polymerase I β-subunit proteins. The Proline mutated in *rpoa-2(op259)* (P70) is conserved from yeast to human. Together with the Proline at 3 positions towards the N-terminus, P70 defines a predicted SH3-domain binding site (PxxP), a motif that in higher eukaryotic orthologs is also present nearby. (A motif search by ScanSite [3] predicted binding of Src, Crk, Grb2, or Abl SH3 domains (low stringency settings)). In the mutated protein P70S, this site is no longer presenting an SH3 binding motif. C) Sequence alignment of the *C. elegans* RNA Pol I, II and III β-subunits. P70, corresponding to the residue that is substituted in the *rpoa-2(op259)* mutant with Serine, and the subsequent amino acids predicted to form an α-helical structure are conserved between the paralogs.
(PDF)

Figure S3 Cytoplasmic enrichment of mutant YFP::RPOA-2(P70S) protein. Expression of transgenic YFP-tagged RPOA-2 protein. Mutant YFP::RPOA-2(P70S) has a visibly increased ratio of cytoplasmic versus nucleolar protein localisation in comparison to wild-type YFP::RPOA-2(wt) (three transgenic lines each). YFP::RPOA-2 abundance is low in the nucleoplasm (outlined by outer and inner dashed circles in the top row; arrowheads in the third row), which makes cytoplasmic fluorescence of mutant YFP::RPOA-2(P70S) clearly visible. Meiotic pachytene region of the adult germ line (top; tangential imaging plane in the first row, central sagittal plane to illustrate the rachis (shared cytoplasm) in the second row); somatic cells of the developing vulva and uterus at L3 stage (middle); and intestinal cells of young adult worms (bottom). *opEx* and *opIs* are extrachromosomal or integrated transgenes, respectively. Exposure has been adjusted between lines to reach similar fluorescence intensity for the nucleoli. Size bar, 12 µm (top), 15 µm (middle), 25 µm (bottom).
(PDF)

Figure S4 Growth and lifespan of *rpoa-2(op259)* mutant animals. A) Reproductive cycles of wild-type and *rpoa-2(op259)* mutant worms grown at 15°C, 20°C, or 25°C, showing the duration of each developmental stage. Adult worms grown under standard conditions were bleached and the synchronised embryos were transferred to fresh plates and raised at the indicated temperature. Time points when the majority of the population had passed a developmental stage transit were recorded. At 20°C, *rpoa-2(op259)* animals have a delay mostly on account of an extended period as young adults (after moulting but prior to egg laying); the duration of young adulthood at 25°C is approximated by rare escaping animals from temperature sensitive sterility. B) Egg laying rate in the first 2 days of adulthood. Staged, well-fed animals were transferred in small groups to fresh plates and allowed to lay eggs for 3–6 hours. Eggs were then counted and the average number of eggs laid per animal per hour was calculated. Average of at least 36 animals and 3 plates per condition, SD of the weighted averages of the plates. The onset of egg laying is delayed to approximately 30 hours post L4 in *rpoa-2(op259)* animals. C) Life span is not extended in *rpoa-2(op259)*, unlike in the translation initiation mutant *ife-2*. Adult worms were transferred onto fresh plates every two days so they would always be clearly distinguishable from the progeny. Death was defined as ceasing of pharyngeal pumping activity, of (head) movements and of reaction to touch. Bagging animals with internal hatching of larvae and animals drying on the dishes were censored. Kaplan-Meier survival curves were calculated using JMP 9 statistical software. The experiment was started with 120 young adult animals per strain. χ^2^ testing was in comparison to N2 wild-type worms.
(PDF)

Figure S5 Germ cell proliferation and differentiation in *rpoa-2(op259)* mutants. A) Germ line organisation in DAPI stained whole worms (maximal intensity projection) 24 hours after the onset of egg laying. Distribution of distinct zones (defined by the characteristic chromatin patterns of germ cell nuclei) is indicated with coloured lines. B) Germ cell content of wild-type or mutant gonads at several time points with respect to the onset of egg laying (reference). Germ cells were classified individually according to their chromatin pattern. Although germ line maturation is delayed in *rpoa-2(op259)* mutants, a numerically similar germ cell composition as in wild type is eventually established. C) Data table to Fig. S5B. Average number of cells ± SD of 4 gonads per data point. Last row indicates average number ± SD of mitotic figures in 8 gonads.
(PDF)

Figure S6 Proximal proliferation in *rpoa-2(op259)* mutants at 25°C. A) Worms were transferred as L1 larvae to the indicated temperature. Distal gonad ends are oriented towards the top left (asterisks). Whereas wild-type gonads tolerate the shift, in *rpoa-2(op259)* mutants, proliferating cells (outlined in black) gradually fill the space between the spermatheca and the most proximal oocytes (triangles) and some residual sperm (arrows). B) About 2 days post L4 larval stage, some gonads have grown into massive tumours (outlined with black line), that most probably arise from the proximal proliferation. At early time points of post-larval development, few cells of the size of mitotic germ cells become apparent in some worms, proximal to sperm; they continuously expand, so the region of spermatogenesis – or later, the most proximal oocyte – migrate further distal or are virtually consumed (arrow indicates some remaining sperm). Cells in the proximal proliferation region have a chromatin pattern that is distinct from the “spaghetti bowl” pattern of late meiotic pachytene cells (short flashes), and that is consistent with mitotic germ cells (circle in distal region, dashed circle in proximal proliferation). Size bar left, 60 µm; right, 15 µm. C) Rescue of the tumor phenotype at 25°C in a transgenic line expressing YFP-tagged wild-type RPOA-2 in an *rpoa-2(op259); unc-119(ed3)* mutant background. Size bar, 20 µm.
(PDF)

Figure S7 The Gogo phenotype in *rpoa-2(op259)* mutant animals. A) *rpoa-2(op259)* mutants are prone to develop distal oocytes (triangles in upper gonad arm) and associated corpses (arrowheads) in the distal arm within a field of meiotic germ cells, far distal to the gonad bend (right side of the picture), leading to a pattern of pachytene stage germ cells – oocyte(s) – pachytene stage germ cells – oocytes (Gogo). In the animal shown, the appearance of the first of the orthotopic oocytes (triangles in the lower gonad arm) is shifted towards the proximal end of the female gonad. Distal oocytes with condensed chromosome pairs (open triangles) are interspersed with nuclei that have a “spaghetti bowl” chromatin pattern characteristic of late meiotic pachytene cells (short arrows, inset), which distinguishes this abnormality from the Pro phenotype (Fig. S6B). One particularly sensitive condition for the Gogo phenotype is irradiation of *rpoa-2(op259)* worms fed on RNAi bacteria (condition shown). B) Schematic of the Pro and Gogo phenotypes found in a fraction of *rpoa-2(op259)* mutant gonads at sensitive conditions, indicating the topology of ectopic germ cell differentiation stages; colour code as in Fig. S5A. C) Distal oocytes in *rpoa-2(op259)* are strongly suppressed by loss of *cep-1* function. They are not suppressed by inhibition of cell death with *ced-3(RNAi)*; however, the associated corpses are lost, indicating that the latter are indeed apoptotic. RNAi treatment was started at L1 stage. Table indicates the fraction of gonads having developed a Gogo phenotype by 48 hours after irradiation (average of at least three experiments for *rpoa-2(op259)* and *cep-1 rpoa-2(op259)*) and the total number of gonads scored per condition; n.d., not determined.
(PDF)

Figure S8 Nucleolar substructures are enlarged in *rpoa-2(op259)* mutants. A) Nucleoli of prominent somatic cells in *rpoa-2(op259)* mutants (germ cell nucleoli are shown in Fig. 2A). The nuclear or nucleolar borders are outlined by the outer and inner white dashed circles, respectively (vulvae at the Christmas tree-stage), or indicated by white arrowheads and white arrows, respectively. The intestinal cells in *rpoa-2(op259)* mutant worms often have smaller nucleoli but larger nucleolar substructures (black arrow) than wild-type worms, and their nuclear borders are less distinct in the mutant due to overlay with the highly abundant intracellular lipid droplets. Size bar, 8 µm. B) CEP-1::GFP *opIs198[P_cep-1_::cep-1::gfp; unc-119(+)]* and YFP::RPOA-2 *opIs372[P_rpoa-2_::yfp::rpoa-2(+); unc-119(+)]* reporters show an inverse localisation pattern in germ cell nuclei: CEP-1::GFP grossly spares the nucleolus except for the nucleolar dot, whereas YFP::RPOA-2 is concentrated in the nucleolus omitting the nucleolar dot. Size bar, 4 µm.
(PDF)

Figure S9 5-Fluorouridine (5-FU) incorporation highlights rRNA synthesis in germ cell nucleoli. A) Gonads were extruded from adult hermaphrodites and incubated for 15 min with 5-FU before fixation and immunostaining with anti-BrdU antibody. All meiotic germ cells show a rapid uptake and accumulation of 5-FU inside the nuclei, likely the result of incorporation into nascent transcripts. Chromatin (DAPI staining) encircles the central 5-FU signal in the prominent germ cell nucleoli. Size bar, 25 µm. B) Transcription in the late meiotic pachytene region (zone of germ cell apoptosis) of wild-type and *rpoa-2(op259)* mutant gonads. Both show a strong signal predominantly in the nucleoli (RNA Pol I transcription) and a weaker signal where chromatin stains (Pol II/III). The mostly annular 5-FU pattern in the nucleoli is consistent with RNA polymerase I localisation as evidenced with YFP::RPOA-2 (Fig. S3). There is no obvious difference between the mutant and wild type in the pattern or fluorescence intensity. Size bar, 15 µm.
(PDF)

Figure S10 Ribosomal proteins in proteome comparison of *rpoa-2(op259)* and wild-type worms. Distribution of the abundance ratios for individual proteins. The abundances of all proteins that could be identified in both *rpoa-2(op259)* and wild-type worms were compared, and the log_2_-ratios were binned (intervals ±0.15 of indicated value). Ribosomal proteins show a clear left-shift in comparison to the pool of non-ribosomal proteins, i.e. lower abundance in *rpoa-2(op259)* mutants. The proteins annotated with GO: terms referring to ‘stress response’ tend toward higher abundance in *rpoa-2(op259)* mutants (bars in blue). The bars at the extremes show the number of proteins identified in only *rpoa-2(op259)* or wild-type samples.
(PDF)

Figure S11 The RNA band running between 26S and 18S rRNA is a 3′-truncated, non-polyadenylated version of the 26S rRNA. A) Representation of the rDNA locus in *C. elegans*. It is a tandem arrangement of approximately 55 repeat units [4] of 7.2 kb length each [5], located in the subtelomeric region of the right arm of chromosome 1 (LG I) [6]. As in higher eukaryotes, the small subunit 18S rRNA and the large subunit 5.8S and 26S rRNAs are separated by internal transcribed spacers (its1 and its2), and the rRNA polycistronic units are flanked by an external transcribed spacer (ets1); all spacers are cleaved off during processing of the pre-rRNA. The rDNA genes are separated by a short intergenic spacer (less than 500 bp; the precise ends of the 5′ and 3′ ETSs in the pre-rRNA transcript of *C. elegans* have not been clearly delimited from the intergenic spacer). Positions of DIG-labelled antisense RNA probes are indicated; and additionally for the 26S rRNA, positions of the DIG-labelled oligo-deoxy-ribonucleotide probes that were used to narrow down the candidate region of the 26S-short end. B) Sequential hybridisation with DIG-labelled RNA probes on total RNA extracts. (Membrane stripping was not complete, as can be judged from the persistence of the 18S rRNA band.) The its2 probe (tested on other blots and not shown) did not hybridise to the 26S-short band. 26S-01c (26S-short band detected) and 26S-07c (band not detected) delimit the candidate region for the break to 600 nucleotides within the 26S rRNA. The band labelled with 26S-s' is detected by the 26S-2 probe and is more pronounced in *rpoa-2(op259)*. Together with the size (∼0.8–1 kb), this suggests that it could represent the counterpart of the 26S-short fragment, resulting from cleavage of the full-length 26S rRNA (not further tested). C) 26S-short rRNA fragment circularisation and reverse transcription strategy. RNA was gel-extracted and circularised. Reverse transcription was performed with primer_076, and PCR (with short extension time) using primers 191 and 076 was performed on the resulting reverse transcripts, which should reach from the 5′ end directly into the 3′ end of the circularised RNA. D) Sequencing analysis of PCR products for the definition of the precise ends of the 26S-short rRNA. Both sequencing directions give a breakpoint at the 5′ and 3′ ends of the 26S-short sequence, respectively, with a transition from a unique sequence into an overlap of two identical sequences shifted by 1 nt and corresponding exactly to the other end of the 26S-short rRNA. The overlap results from a T (U) that is present in approximately half the template. E) The truncation site (red U) is predicted to be within the loop of a stem-loop structure mapping to the highly variable region D10 in the first sequence and structure analysis of the *C. elegans* rRNA [7]; and to the expansion segment ES31 in the homologous region of the yeast rRNA [8]. Numbers indicate nucleotide position within the 26S rRNA.
(PDF)

Figure S12 Translation-inhibition with cycloheximide (CHX) blocks irradiation-induced apoptosis and strongly affects animal health. A) Apoptotic germ cell corpses 18 hours after irradiation. Adult worms were transferred to freshly prepared CHX plates for 6 hours before irradiation and kept on the drug. The progeny were assessed at 2 and 4 days after treatment initiation for developmental delay or arrest. A significant reduction of corpses occurred in conditions that also had a strong effect on germ line integrity and animal growth. Average number of corpses and SD of at least 16 animals per condition. B) Accumulation of corpses in the engulfment mutant background *ced-6(n1813)*. Young adult worms were transferred to freshly prepared CHX plates and exposed for 16 hours. Effect of CHX on constitutive cell death is weak at intermediate doses in the engulfment single mutant, but pronounced in combination with the *rpoa-2(op259)* mutation, indicating that constitutive cell death depends on new protein synthesis in this mutant. Average number of corpses and SD of at least 20 animals per condition.
(PDF)

Figure S13 *rpoa-2(op259)* suppresses excessive apoptosis in *rad-51(lf)* and *abl-1(lf)* mutants. A) Current model of apoptosis induction in *C. elegans*. Two modes have classically been distinguished for the apoptotic death of germ cells, mainly by their dependence on *cep-1* and *egl-1*. During somatic development, transcriptional regulation of EGL-1 is key for the lineage-specific induction of most cell deaths. EGL-1 physically disrupts the inhibitory binding of CED-9 to CED-4, which in turn serves as platform for CED-3 activation [9]. For DNA damage-induced germ cell death, EGL-1 and CED-13 are transcriptionally up-regulated by CEP-1/p53. By contrast, physiological germ cell death is largely CEP-1- and EGL-1-independent. *abl-1(lf)* and *rad-51(lf)* were shown to increase CEP-1 dependent cell death [10], [11]. B) Apoptotic response to IR irradiation (60 Gy) in the *abl-1(ok171)* kinase mutant background. Error bars, CI 95% of the mean number of germ cell corpses per gonad (n = 15). C) Baseline apoptosis (0 Gy, straight lines) and response to IR irradiation (60 Gy, dashed lines) in the *rad-51(lg8701)* DNA repair-mutant background. *rad-51(lg8701)* homozygous animals were derived from the balanced strains *rad-51(lg8701)/nT1* and *rpoa-2(op259); rad-51(lg8701)/nT1* and irradiated as young adults. Error bars, CI 95% of the mean number of germ cell corpses per gonad (n = 15).
(PDF)

Figure S14 Expression of CEP-1 and core apoptotic factors is not significantly altered in *rpoa-2(op259)*. A) qRT-PCR analysis of core apoptotic factors in whole-worm extracts. Average normalised levels of at least 7 independent samples per condition. Relative transcript levels in adult *rpoa-2(op259)* worms are very similar to wild type, except for CED-9 mRNA, which has a moderate but statistically significant decrease in *rpoa-2(op259)* (*p*-value of *t*-test indicated). B) CEP-1::GFP expression in germ cells of the late meiotic pachytene region. Gonads were extruded from adult worms on the second day of adulthood. Fluorescence pattern and intensity are similar between *rpoa-2(op259)* and wild-type gonads. Of note, the nucleoli in *rpoa-2(op259)* germ cells often have a more pronounced nucleolar dot (visible by DIC), that is positive for CEP-1::GFP (see also Fig. S8B). Size bar, 15 µm. C) CED-4::GFP expression (*opIs219*) in meiotic germ cells. Perinuclear signal intensity in the late meiotic pachytene region increases slightly upon irradiation. No obvious difference in signal intensity or pattern between wild type and mutant. Size bar, 20 µm.
(PDF)

Figure S15 *rpoa-2(op259)* enhances the pro-apoptotic effect of the *ced-9(n1653)* mutant but suppresses the effect of *ced-9(RNAi)*. A) Baseline apoptosis in the temperature-sensitive *ced-9(n1653)* and *rpoa-2(op259); ced-9(n1653)* mutants. The reduction-of-function mutation *ced-3(n2438)* reduces the high corpse number of *ced-9(n1653)* mutants, consistent with the known genetic epistasis. The *rpoa-2(op259)* mutation, however, accentuates the increased corpse number. Average number of corpses and SD of at least 40 animals per condition. B) When shifted from 15° to 20°C at the L4 stage, most of the *rpoa-2(op259); ced-9(n1653)* animals become sterile due to excessive apoptosis, which eventually affects all proximal germ cells in the course of adulthood (red dotted line). Excessive apoptosis is suppressed when *egl-1* function is lost by the *egl-1(n1084 n3082)* allele. Average number of corpses and SD of at least 48 animals per condition. C) Germ cell apoptosis in animals treated with *ced-9(RNAi)* (starting at L1) and irradiated as young adults. *ced-9(RNAi)* does not significantly increase the number of corpses in *rpoa-2(op259)*; however, knockdown of *ced-9* strongly sensitises the mutant for irradiation-induced apoptosis. Average number of corpses at 24 hours after irradiation and SD of at least 40 animals per condition.
(PDF)

Figure S16 Knock-down of *gla-3* sensitises for irradiation-induced apoptosis, which is antagonised by *rpoa-2(op259)*. A) Germ cell apoptosis in animals treated with RNAi to *gla-3* (starting at L1) and irradiated as young adults. *gla-3(RNAi)* does not only increase constitutive cell death in wild-type worms but also significantly increases the response to irradiation; this sensitisation is partly *cep-1*-independent. In *rpoa-2(op259)* mutants, *gla-3(RNAi)* does not strongly increase baseline apoptosis; however, knockdown of *gla-3* strongly sensitises the mutant for irradiation-induced apoptosis. Average number of corpses at 24 hours after irradiation and SD of at least 40 animals per condition. B) Loss of *lip-1* function or a gain-of-function mutation of *let-60* restores baseline germ cell apoptosis in *cep-1 rpoa-2*. Average number of corpses and SD of at least 40 animals at 24 hours of adulthood.
(PDF)

Figure S17 LIP-1 expression and localisation is altered in *rpoa-2(op259)* mutants. A) Expression of the transcriptional reporter *zhIs4[P_lip-1_::GFP; unc-119(+)]* in L3 and L4 stage larvae. Top panel, prominent fluorescence in hypodermal cells of *rpoa-2(op259)* animals. Size bar 15 µm. Second panel, LIP-1 expression is reduced in vulval cells of the mutant at different developmental stages; contrast with the stronger signal in hypodermal cells. The asymmetric expression in wild-type vulvae was consistently observed in multiple animals. The reporter is not expressed in the germ line. Size bar 15 µm. B) Immunostaining for LIP-1 at 18 hours after the onset of egg laying (see Text S1). The membrane-associated signal ubiquitously found in the gonads of wild-type worms is not found in the late pachytene region of *rpoa-2(op259)* mutant germ lines. Control staining with an anti-DNA antibody; same exposure settings in all three lines. Size bar 12 µm.
(PDF)

Figure S18 Multiple stress response factors are upregulated in *rpoa-2(op259)* mutants. Expression in L4 stage larvae of transcriptional reporters for GST-4 (*dvIs19[P_gst-4_::GFP::NLS]*; oxidative stress), NLP-29 (*frIs7[P_nlp-29_::GFP+P_col-12_::DsRed]*; infection, wounding, osmotic stress), SOD-3 (*muIs84[P_sod-3_::GFP+rol-6]*; oxidative stress), and HSP-6 (*zcIs13[P_hsp-6_::GFP]*; heat shock). Worms are oriented with the head to the right and ventral body part to the bottom. Overview (10× lens) and higher magnification (40×) exposures; size bar 120 µm or 20 µm, respectively.
(PDF)

Table S1 Eukaryotic RNA polymerase I/II/III subunits.Common names (grey column) and the human and yeast names of the subunits of the three polymerases are indicated. Shared subunits are in blue (all three nuclear RNA polymerases), or green (Pol I and Pol III). In *C. elegans*, the homologs of the Pol II subunits had been defined and accordingly named *rpb-x* (except for the largest and the two accessory subunits; Wormbase WS200). Homologs of the Pol I and Pol III subunits were assembled from Wormbase database entries and from BLAST searches. For the accessory subunits of the core transcription apparatus of RNA pol I (A14, A43, A34.5), the *C. elegans* homologs remain to be identified (n.i.). “*rpa-1*” and “*rpa-2*” have already been attributed to other genes and cannot be used to name the RNA pol I (‘A’) subunits 1 and 2, hence the gene names *rpoa-x* for RNA pol I subunits. The table was mainly assembled from NCBI Homologene and literature searches [12], [13].
(PDF)

Table S2 RNAi knockdown of individual RNA polymerase subunits does not generally reduce irradiation-induced apoptosis. RNAi knockdown experiments and observed effects on fertility, development, and apoptosis. Synchronised L3 stage worms were transferred to RNAi bacteria, which often led to a visible effect by the time the worms reached adulthood. # experiments indicates the number of independent experiments with either the Ahringer (Ahr) and ORFeome (ORF) RNAi bacterial clones (where ‘0’ means no clones available). Egg laying was categorised as normal, reduced, or strongly reduced (few eggs). Hatching: + normal, most embryos hatch; (+) several non-hatched eggs; (−) mostly non-hatched eggs. Number of apoptotic corpses per gonad at 24 hours after irradiation are shown, as well as SD and total number of animals tested per condition. For knockdown of the ABC27, Rpb2 and Rpb3 homologs together with irradiation, germ line defects were so strong that scoring of apoptotic corpses was no longer possible (n.d.). Consistently, knockdown of *rpb-12*, of *rpb-4*, of F23F1.9 (A49), of Y77E11A.6 (Rpb9 paralog C11), or of Y39B6A.36 (RAP30) did not strongly affect viability or reproduction of the treated parental generation or their F1 progeny. Shared subunits between Pol I and Pol III are indicated with a green bar, and between all three polymerases with a blue bar.
(PDF)

Table S3 Interfering with ribosome synthesis abolishes IR-induced apoptosis. Knockdown of various rRNA processing factors and of ribosomal proteins reduces apoptotic response to irradiation in the germ line. Synchronised L3 stage worms were transferred to RNAi bacteria, which often led to a visible effect by the time the worms reached adulthood. For each RNAi bacterial clone (Gene sequence identifier and gene name where available), the source is indicated as Ahringer library (Ahr) or ORFeome RNAi library (ORF). RNAi target genes are arranged according to an approximate chain of action on ribosomal small subunit (SSU) or large subunit (LSU) synthesis, or ribosome degradation. *fib-1* is a major nucleolar protein; *rrbs-1* is important for 5S incorporation into the large subunit, *eri-1* was shown to be involved in 5.8S rRNA processing. Arbitrarily, *rpl-1*, *rpl-2*, *rps-1*, *rps-2* were selected for testing ribosomal proteins (RPs). P-P indicates the factors for which a physical protein interaction with RPOA-2 has been predicted (Wormbase WS200). Data represent average number of corpses per gonad 24 hours after irradiation, ±SD, and the total number of animals scored per condition; n.d., not determined. For *opIs110; unc-119(ed3)*, corpses were scored by DIC and independently by the number of Actin::YFP halos forming from sheath cell protrusions around corpses (reporter *opIs110[P_lim-7_::act-5::yfp; unc-119(+)]*).
(PDF)

Table S4 Primers used in this study. Primer sequences with internal numbering (ID) and names.
(PDF)

Text S1 Supplementary Results, Supplementary Methods, and Supplementary References. The citations referred to in the Supplementary Figure legends are all listed as Supplementary References in Text S1.
(PDF)
