# Supplementary figures and images for: Ribosome Synthesis and MAPK Activity Modulate Ionizing Radiation-Induced Germ Cell Apoptosis in Caenorhabditis elegans
Source: PLoS Genet. 2013 Nov 21;9(11):e1003943. doi: 10.1371/journal.pgen.1003943 (PMC3836707; doi:10.1371/journal.pgen.1003943)

**A**

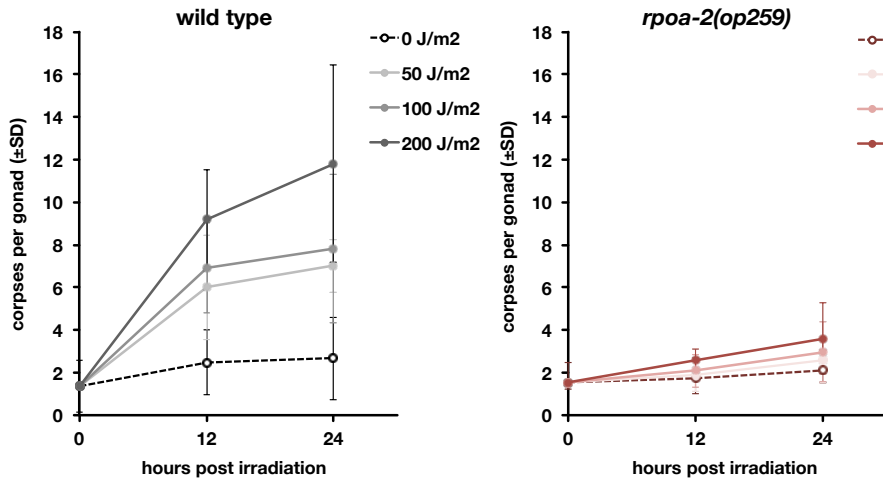

**B**

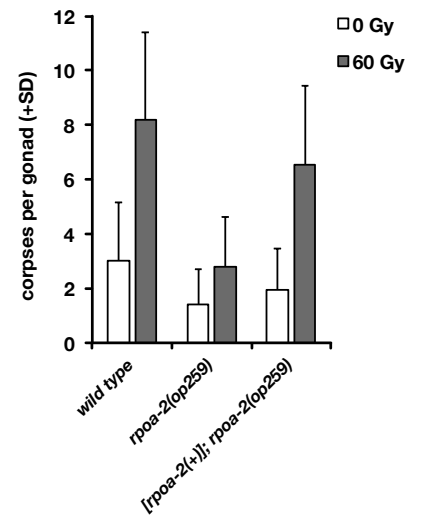

**C**

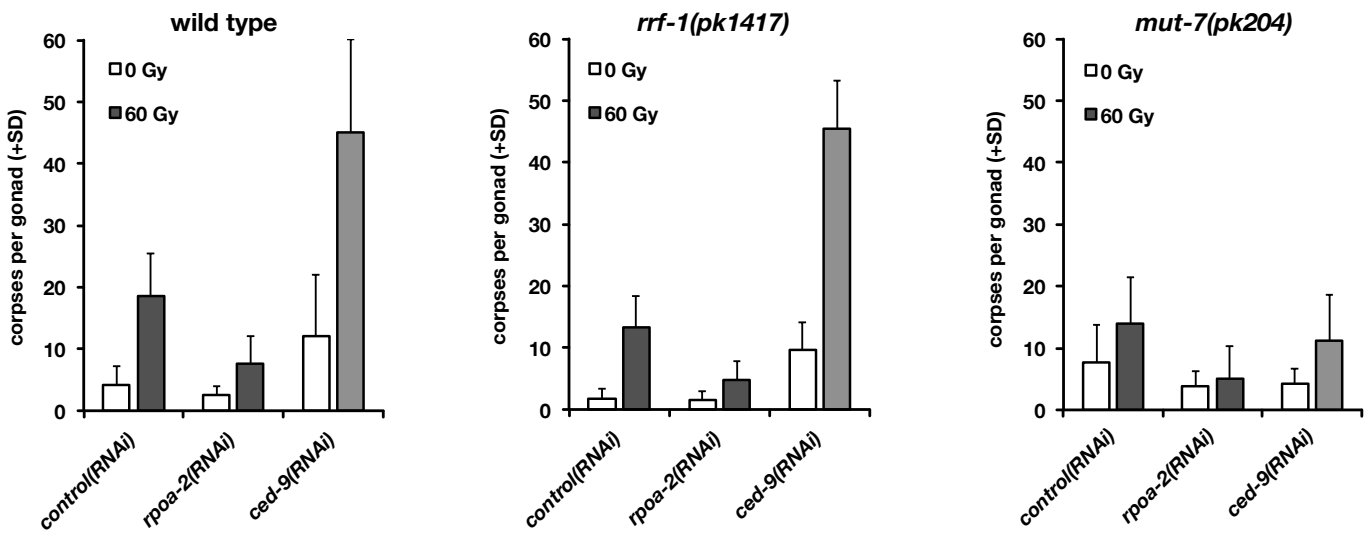

**D**

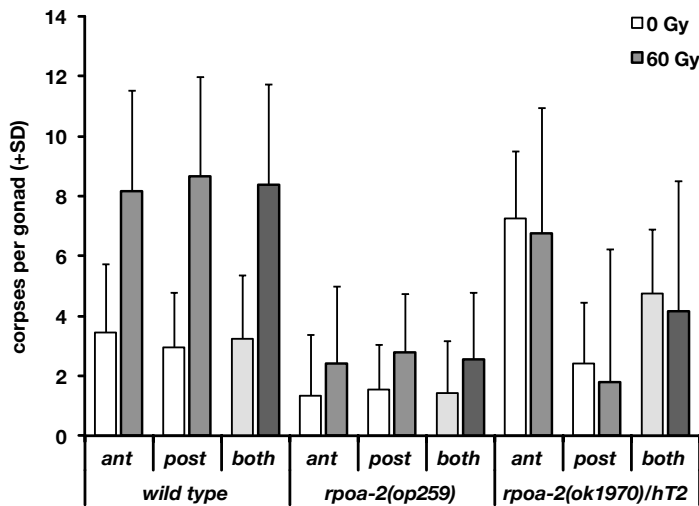

**E**

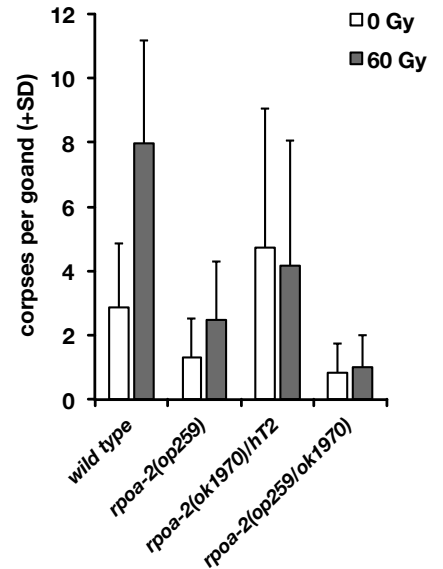

Supplement: Figure S1 — DNA damage-induced apoptosis in animals with mutations or knockdown of rpoa-2. A) rpoa-2(op259) mutants fail to induce apoptosis following UV-C irradiation (254 nm). Error bars, SD; n = 20 animals per condition. B) Rescue of the apoptotic phenotype with wild-type rpoa-2 [rpoa-2(+)] from the transgene opEx1416[Prpoa-2rpoa-2(+)::rpoa-2 3′UTR; unc-119(+)]. Error bars, SD of at least 40 gonads. C) Knockdown of rpoa-2 in the whole animal (wild-type worms), or specifically in the germ line (rrf-1 mutants) or in the soma (mut-7 mutants) all affect germ cell apoptosis. rrf-1(pk1417), carrying a mutation in the RNA-directed RNA polymerase RdRP (QDE-1) homolog, selectively abolishes RNAi effects in somatic cells [1], thus exhibiting germ line-specific knockdown, whereas mut-7(pk204), a mutation in the RNaseD homolog, affects the RNAi machinery specifically in the germ line [2] and therefore exhibits soma-specific knockdown. rpoa-2(RNAi) was started at L3/L4 larval stage; ced-9(RNAi) (started at L1 stage) is included as a control for a germ cell autonomous gene in apoptosis regulation. Error bars, SD; n>15 animals per condition. D) Germ line apoptosis in animals with a balanced deletion of the rpoa-2 gene at 24 hours after irradiation. Anterior (ant) and posterior (post) gonads were grouped separately due to obviously different levels of germ cell corpses in the balanced rpoa-2(ok1970)/+ strain. Error bars, SD of at least 40 gonads. E) Germ line apoptosis in transheterozygous rpoa-2(op259/ok1970) animals, which were generated by crossing rpoa-2(op259) males with rpoa-2(ok1970)/hT2 hermaphrodites. Error bars, SD of at least 24 gonads. (PDF) [file pgen.1003943.s001.pdf]

A

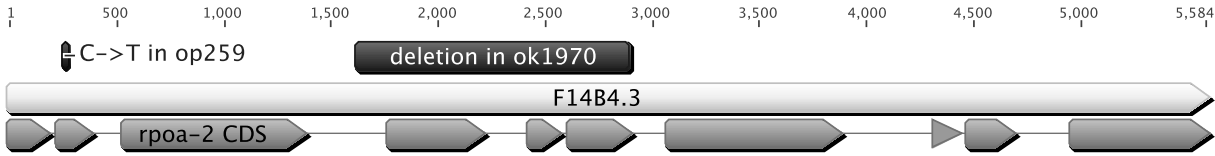

B

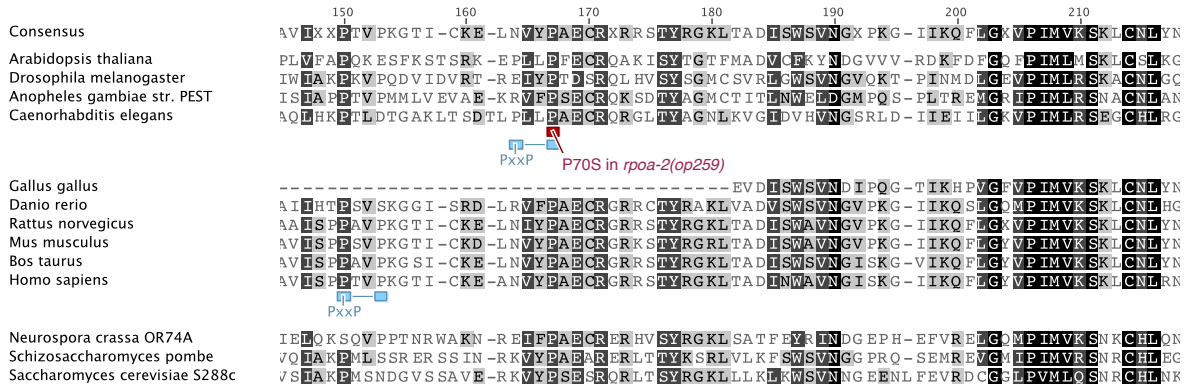

C

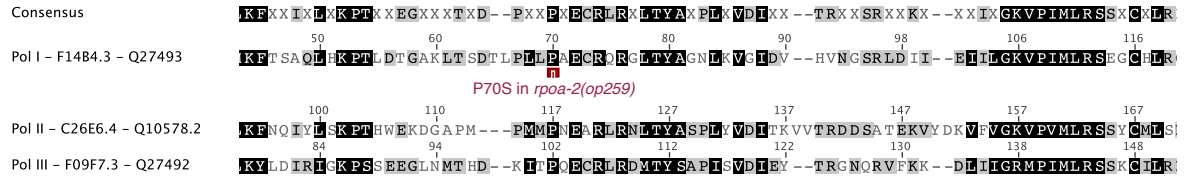

Supplement: Figure S2 — RPOA-2 is a highly conserved nucleolar protein. A) Overview of the F14B4.3 locus (rpoa-2 gene); positions of the single base transition in rpoa-2(op259) and the deletion of a 1.2 kb genomic fragment in ok1970 are shown. B) Sequence alignment of eukaryotic RNA polymerase I β-subunit proteins. The Proline mutated in rpoa-2(op259) (P70) is conserved from yeast to human. Together with the Proline at 3 positions towards the N-terminus, P70 defines a predicted SH3-domain binding site (PxxP), a motif that in higher eukaryotic orthologs is also present nearby. (A motif search by ScanSite [3] predicted binding of Src, Crk, Grb2, or Abl SH3 domains (low stringency settings)). In the mutated protein P70S, this site is no longer presenting an SH3 binding motif. C) Sequence alignment of the C. elegans RNA Pol I, II and III β-subunits. P70, corresponding to the residue that is substituted in the rpoa-2(op259) mutant with Serine, and the subsequent amino acids predicted to form an α-helical structure are conserved between the paralogs. (PDF) [file pgen.1003943.s002.pdf]

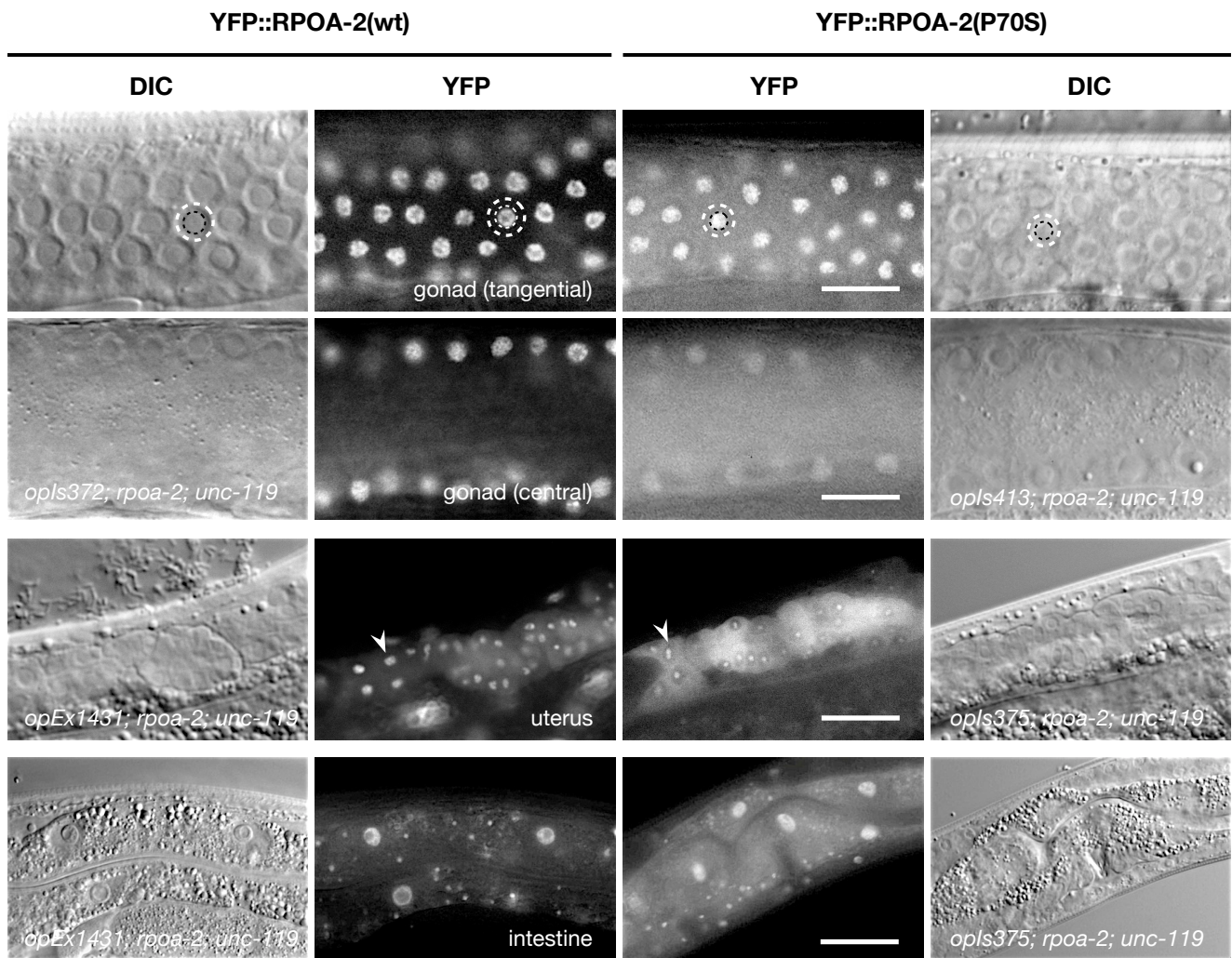

Supplement: Figure S3 — Cytoplasmic enrichment of mutant YFP::RPOA-2(P70S) protein. Expression of transgenic YFP-tagged RPOA-2 protein. Mutant YFP::RPOA-2(P70S) has a visibly increased ratio of cytoplasmic versus nucleolar protein localisation in comparison to wild-type YFP::RPOA-2(wt) (three transgenic lines each). YFP::RPOA-2 abundance is low in the nucleoplasm (outlined by outer and inner dashed circles in the top row; arrowheads in the third row), which makes cytoplasmic fluorescence of mutant YFP::RPOA-2(P70S) clearly visible. Meiotic pachytene region of the adult germ line (top; tangential imaging plane in the first row, central sagittal plane to illustrate the rachis (shared cytoplasm) in the second row); somatic cells of the developing vulva and uterus at L3 stage (middle); and intestinal cells of young adult worms (bottom). opEx and opIs are extrachromosomal or integrated transgenes, respectively. Exposure has been adjusted between lines to reach similar fluorescence intensity for the nucleoli. Size bar, 12 µm (top), 15 µm (middle), 25 µm (bottom). (PDF) [file pgen.1003943.s003.pdf]

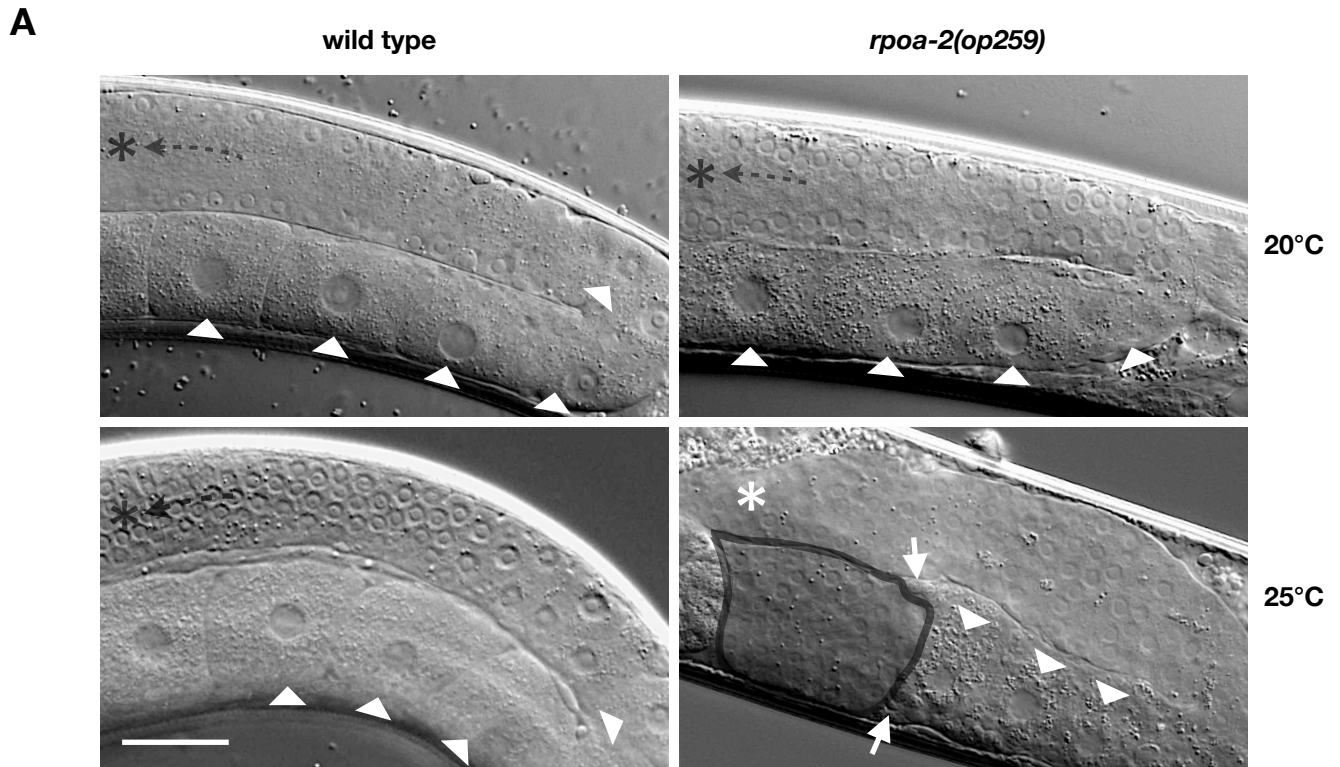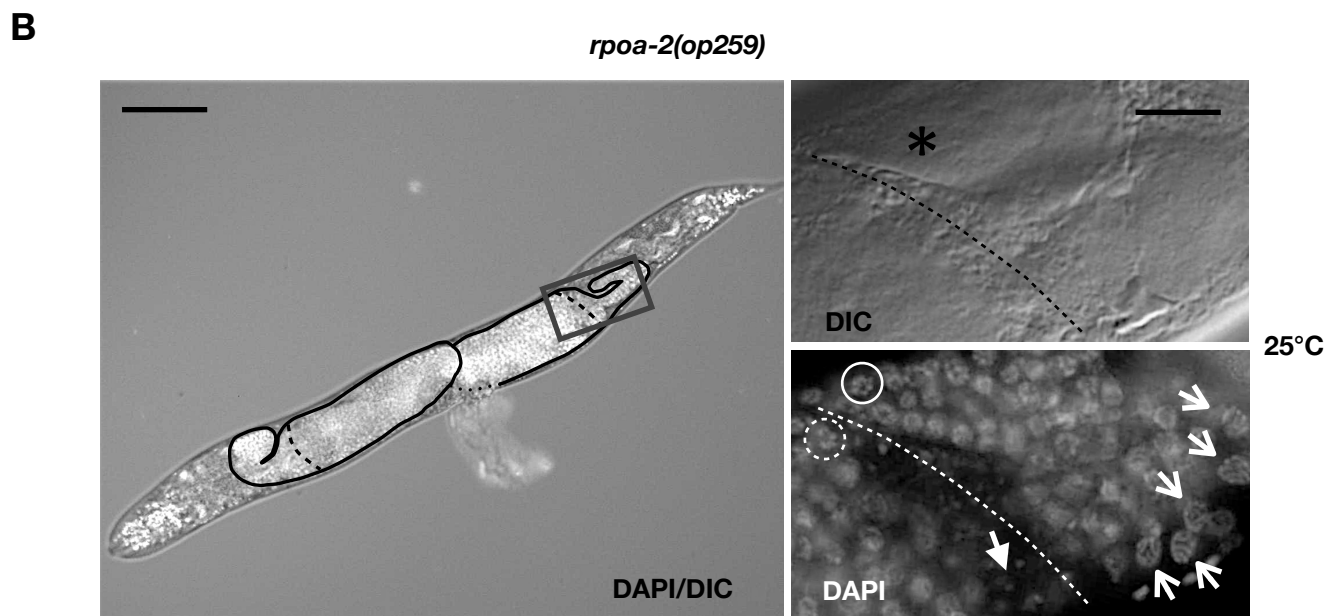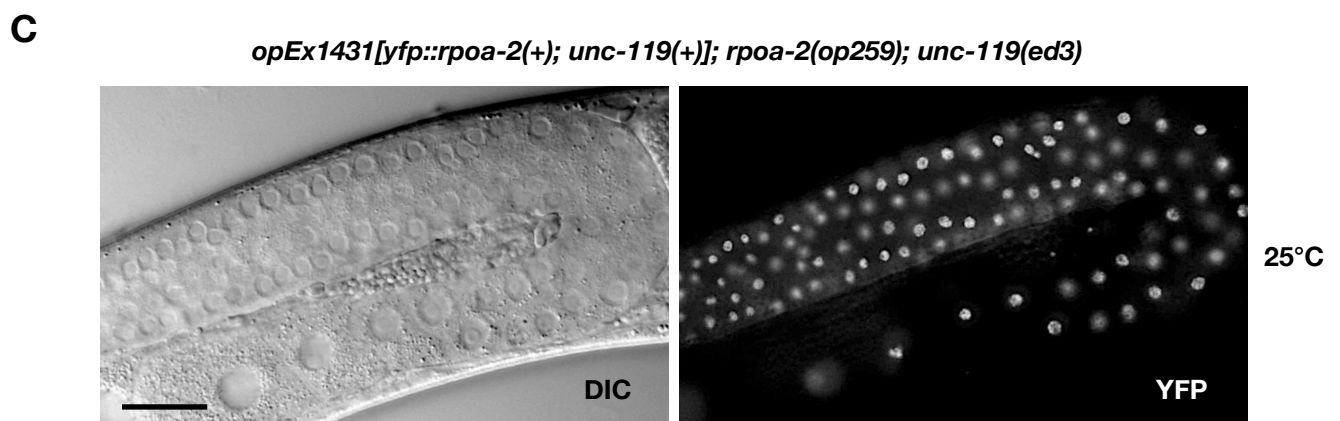

Supplement: Figure S6 — Proximal proliferation in rpoa-2(op259) mutants at 25°C. A) Worms were transferred as L1 larvae to the indicated temperature. Distal gonad ends are oriented towards the top left (asterisks). Whereas wild-type gonads tolerate the shift, in rpoa-2(op259) mutants, proliferating cells (outlined in black) gradually fill the space between the spermatheca and the most proximal oocytes (triangles) and some residual sperm (arrows). B) About 2 days post L4 larval stage, some gonads have grown into massive tumours (outlined with black line), that most probably arise from the proximal proliferation. At early time points of post-larval development, few cells of the size of mitotic germ cells become apparent in some worms, proximal to sperm; they continuously expand, so the region of spermatogenesis – or later, the most proximal oocyte – migrate further distal or are virtually consumed (arrow indicates some remaining sperm). Cells in the proximal proliferation region have a chromatin pattern that is distinct from the “spaghetti bowl” pattern of late meiotic pachytene cells (short flashes), and that is consistent with mitotic germ cells (circle in distal region, dashed circle in proximal proliferation). Size bar left, 60 µm; right, 15 µm. C) Rescue of the tumor phenotype at 25°C in a transgenic line expressing YFP-tagged wild-type RPOA-2 in an rpoa-2(op259); unc-119(ed3) mutant background. Size bar, 20 µm. (PDF) [file pgen.1003943.s006.pdf]

**A**

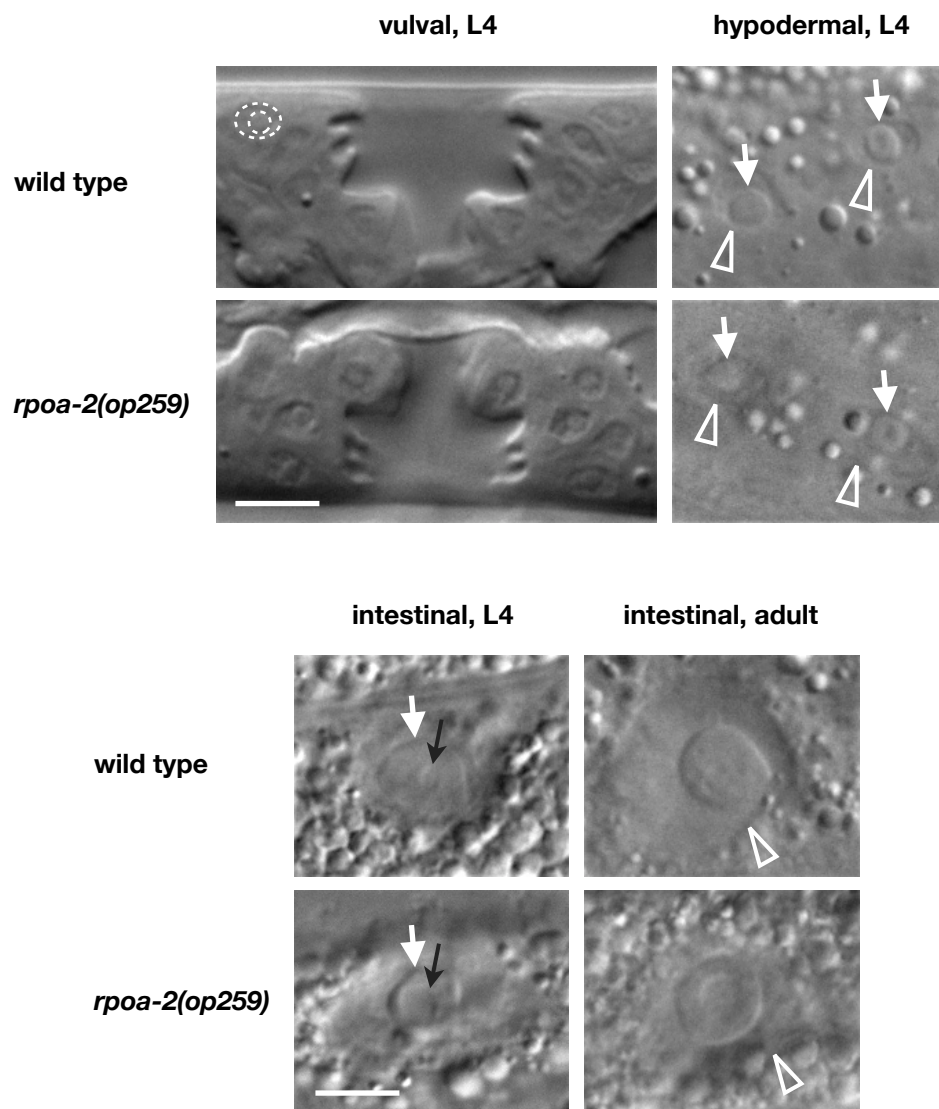

**B**

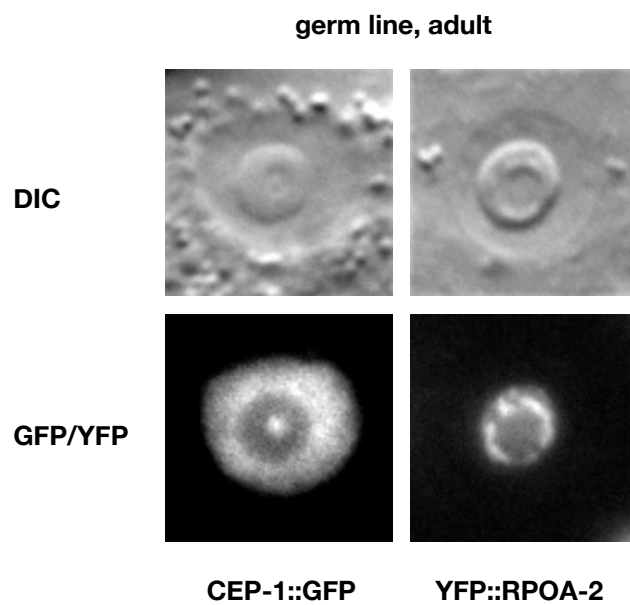

Supplement: Figure S8 — Nucleolar substructures are enlarged in rpoa-2(op259) mutants. A) Nucleoli of prominent somatic cells in rpoa-2(op259) mutants (germ cell nucleoli are shown in Fig. 2A). The nuclear or nucleolar borders are outlined by the outer and inner white dashed circles, respectively (vulvae at the Christmas tree-stage), or indicated by white arrowheads and white arrows, respectively. The intestinal cells in rpoa-2(op259) mutant worms often have smaller nucleoli but larger nucleolar substructures (black arrow) than wild-type worms, and their nuclear borders are less distinct in the mutant due to overlay with the highly abundant intracellular lipid droplets. Size bar, 8 µm. B) CEP-1::GFP opIs198[Pcep-1::cep-1::gfp; unc-119(+)] and YFP::RPOA-2 opIs372[Prpoa-2::yfp::rpoa-2(+); unc-119(+)] reporters show an inverse localisation pattern in germ cell nuclei: CEP-1::GFP grossly spares the nucleolus except for the nucleolar dot, whereas YFP::RPOA-2 is concentrated in the nucleolus omitting the nucleolar dot. Size bar, 4 µm. (PDF) [file pgen.1003943.s008.pdf]

**A**

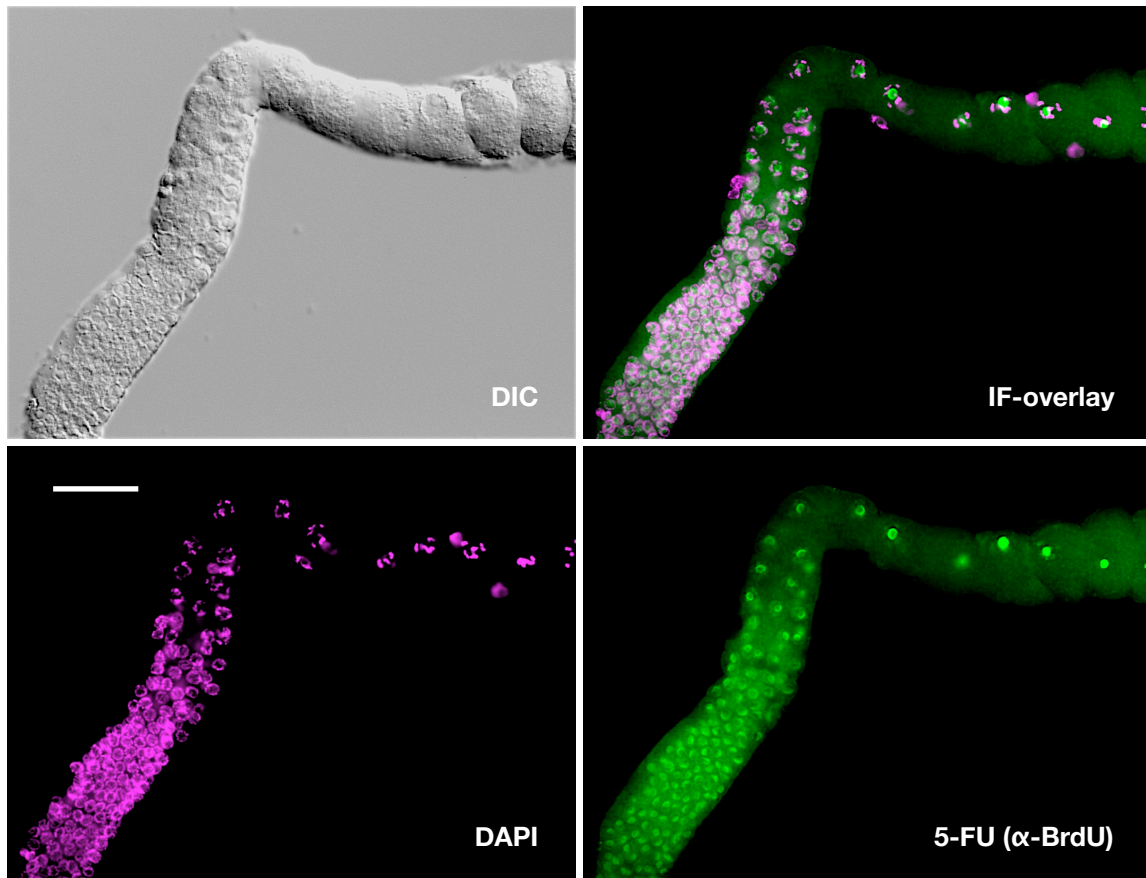

**B**

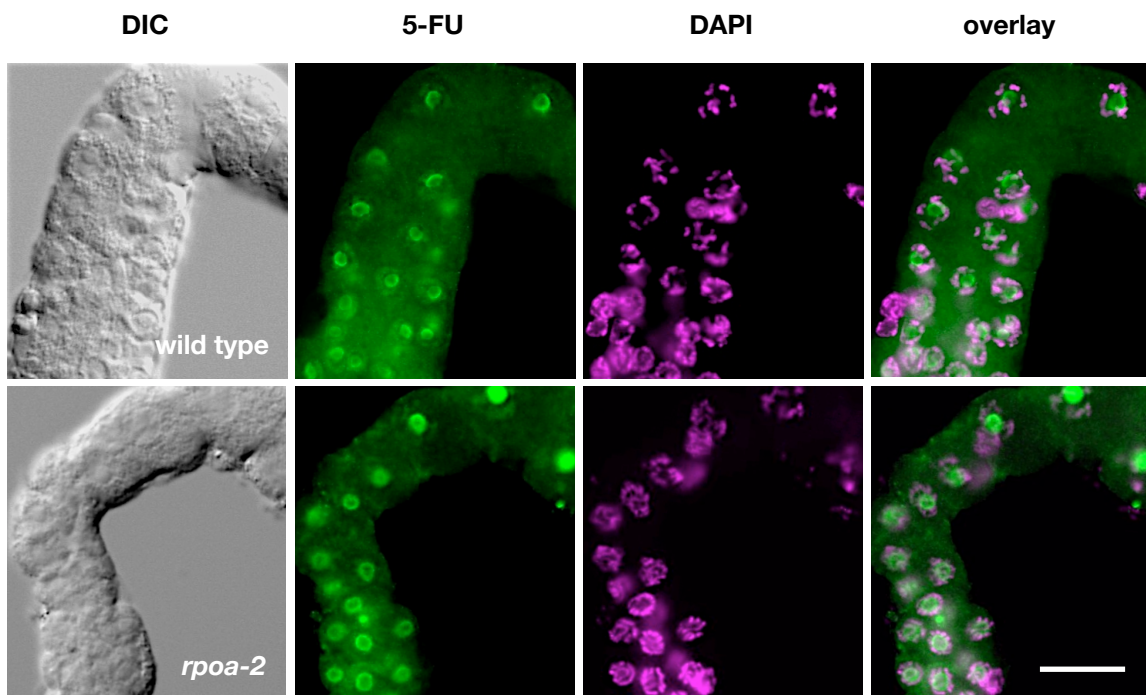

Supplement: Figure S9 — 5-Fluorouridine (5-FU) incorporation highlights rRNA synthesis in germ cell nucleoli. A) Gonads were extruded from adult hermaphrodites and incubated for 15 min with 5-FU before fixation and immunostaining with anti-BrdU antibody. All meiotic germ cells show a rapid uptake and accumulation of 5-FU inside the nuclei, likely the result of incorporation into nascent transcripts. Chromatin (DAPI staining) encircles the central 5-FU signal in the prominent germ cell nucleoli. Size bar, 25 µm. B) Transcription in the late meiotic pachytene region (zone of germ cell apoptosis) of wild-type and rpoa-2(op259) mutant gonads. Both show a strong signal predominantly in the nucleoli (RNA Pol I transcription) and a weaker signal where chromatin stains (Pol II/III). The mostly annular 5-FU pattern in the nucleoli is consistent with RNA polymerase I localisation as evidenced with YFP::RPOA-2 (Fig. S3). There is no obvious difference between the mutant and wild type in the pattern or fluorescence intensity. Size bar, 15 µm. (PDF) [file pgen.1003943.s009.pdf]

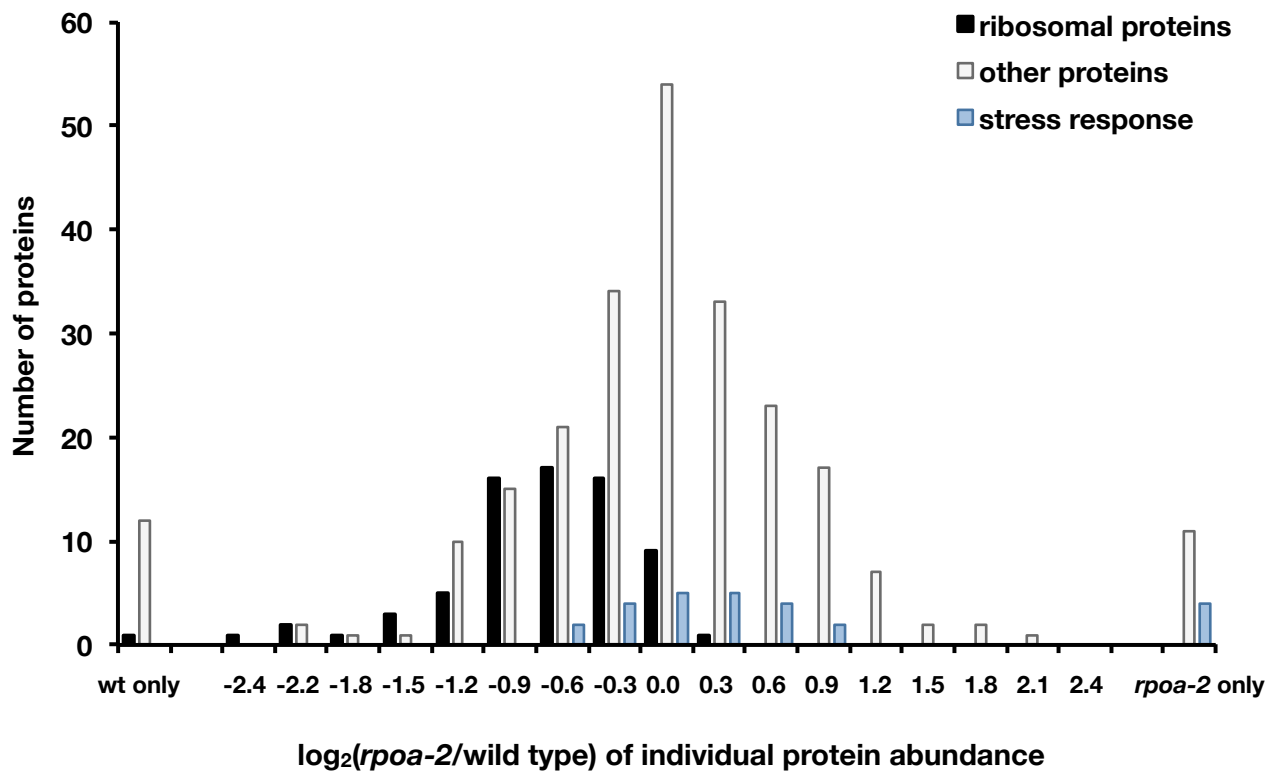

Supplement: Figure S10 — Ribosomal proteins in proteome comparison of rpoa-2(op259) and wild-type worms. Distribution of the abundance ratios for individual proteins. The abundances of all proteins that could be identified in both rpoa-2(op259) and wild-type worms were compared, and the log2-ratios were binned (intervals ±0.15 of indicated value). Ribosomal proteins show a clear left-shift in comparison to the pool of non-ribosomal proteins, i.e. lower abundance in rpoa-2(op259) mutants. The proteins annotated with GO: terms referring to ‘stress response’ tend toward higher abundance in rpoa-2(op259) mutants (bars in blue). The bars at the extremes show the number of proteins identified in only rpoa-2(op259) or wild-type samples. (PDF) [file pgen.1003943.s010.pdf]

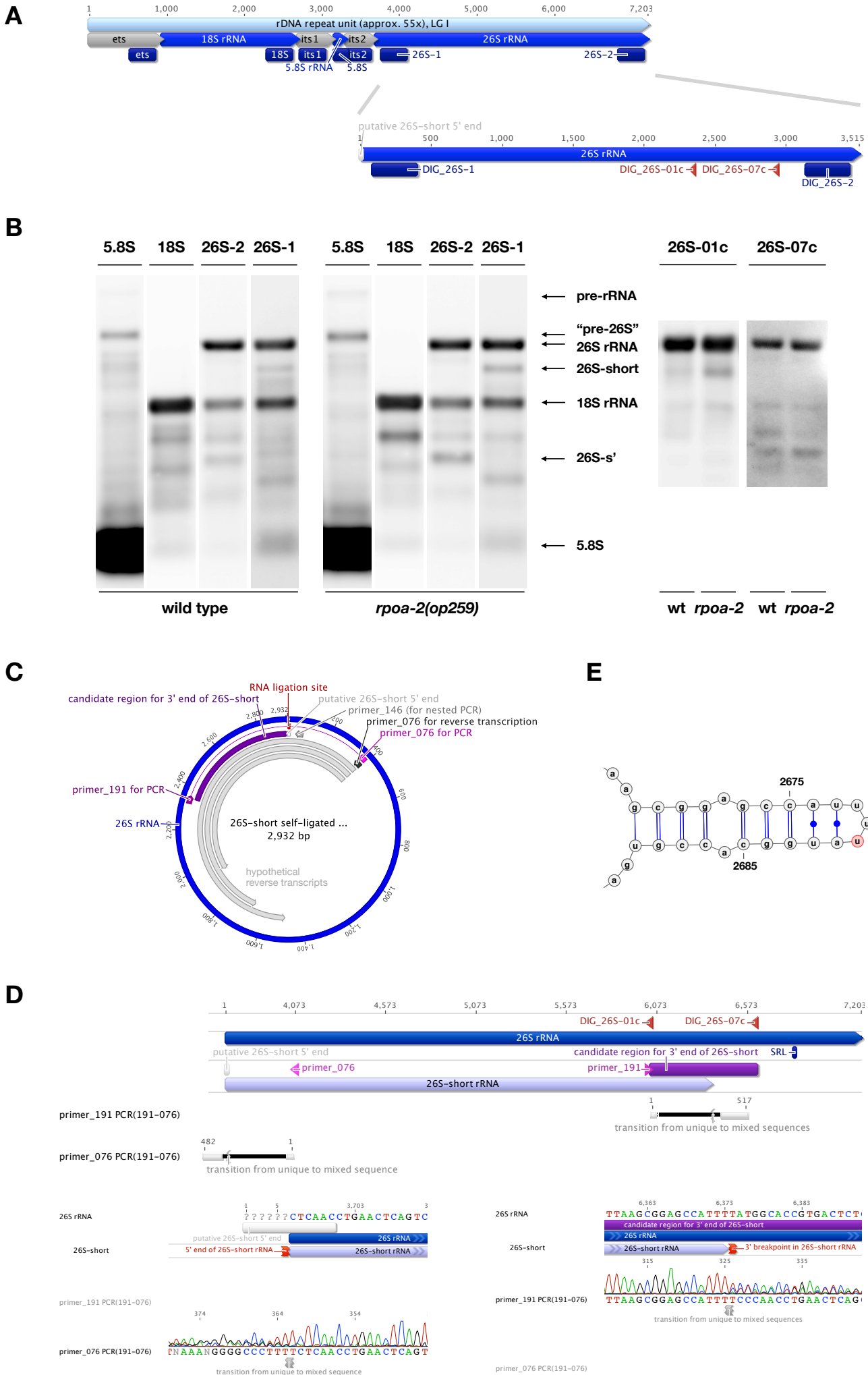

Supplement: Figure S11 — The RNA band running between 26S and 18S rRNA is a 3′-truncated, non-polyadenylated version of the 26S rRNA. A) Representation of the rDNA locus in C. elegans. It is a tandem arrangement of approximately 55 repeat units [4] of 7.2 kb length each [5], located in the subtelomeric region of the right arm of chromosome 1 (LG I) [6]. As in higher eukaryotes, the small subunit 18S rRNA and the large subunit 5.8S and 26S rRNAs are separated by internal transcribed spacers (its1 and its2), and the rRNA polycistronic units are flanked by an external transcribed spacer (ets1); all spacers are cleaved off during processing of the pre-rRNA. The rDNA genes are separated by a short intergenic spacer (less than 500 bp; the precise ends of the 5′ and 3′ ETSs in the pre-rRNA transcript of C. elegans have not been clearly delimited from the intergenic spacer). Positions of DIG-labelled antisense RNA probes are indicated; and additionally for the 26S rRNA, positions of the DIG-labelled oligo-deoxy-ribonucleotide probes that were used to narrow down the candidate region of the 26S-short end. B) Sequential hybridisation with DIG-labelled RNA probes on total RNA extracts. (Membrane stripping was not complete, as can be judged from the persistence of the 18S rRNA band.) The its2 probe (tested on other blots and not shown) did not hybridise to the 26S-short band. 26S-01c (26S-short band detected) and 26S-07c (band not detected) delimit the candidate region for the break to 600 nucleotides within the 26S rRNA. The band labelled with 26S-s' is detected by the 26S-2 probe and is more pronounced in rpoa-2(op259). Together with the size (∼0.8–1 kb), this suggests that it could represent the counterpart of the 26S-short fragment, resulting from cleavage of the full-length 26S rRNA (not further tested). C) 26S-short rRNA fragment circularisation and reverse transcription strategy. RNA was gel-extracted and circularised. Reverse transcription was performed with primer_076, and PCR (with short exte [file pgen.1003943.s011.pdf]

**A**

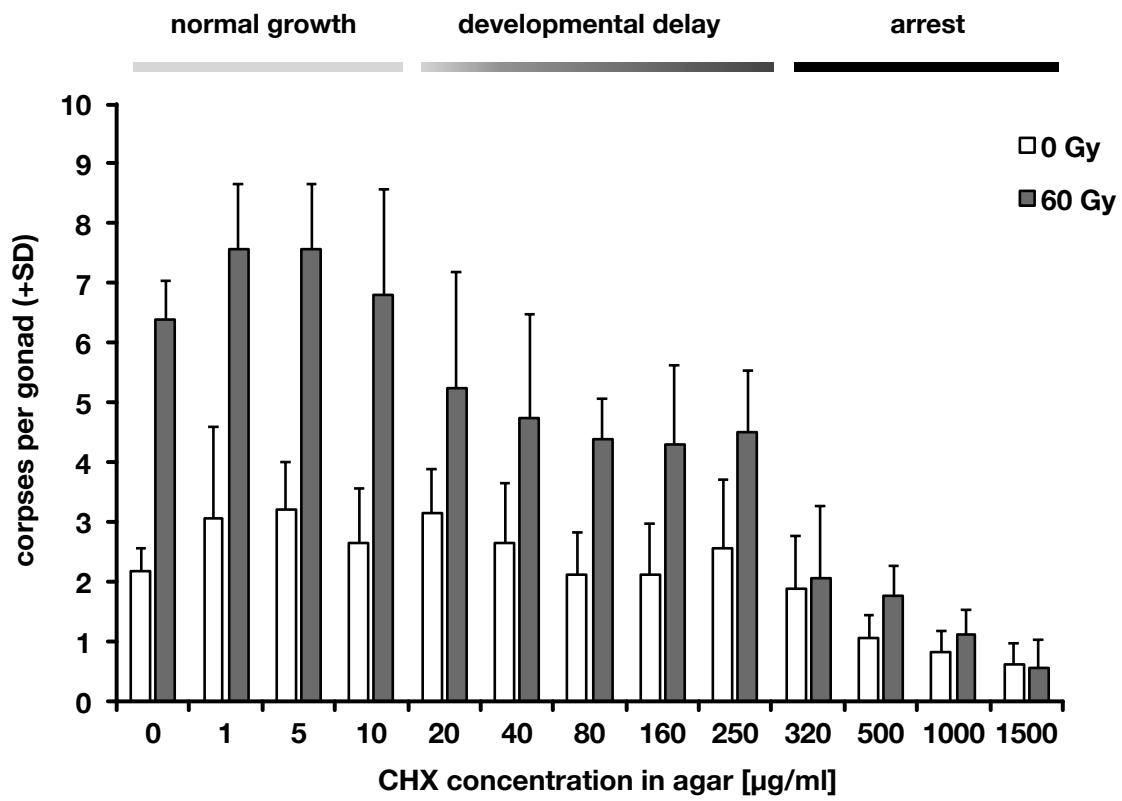

**B**

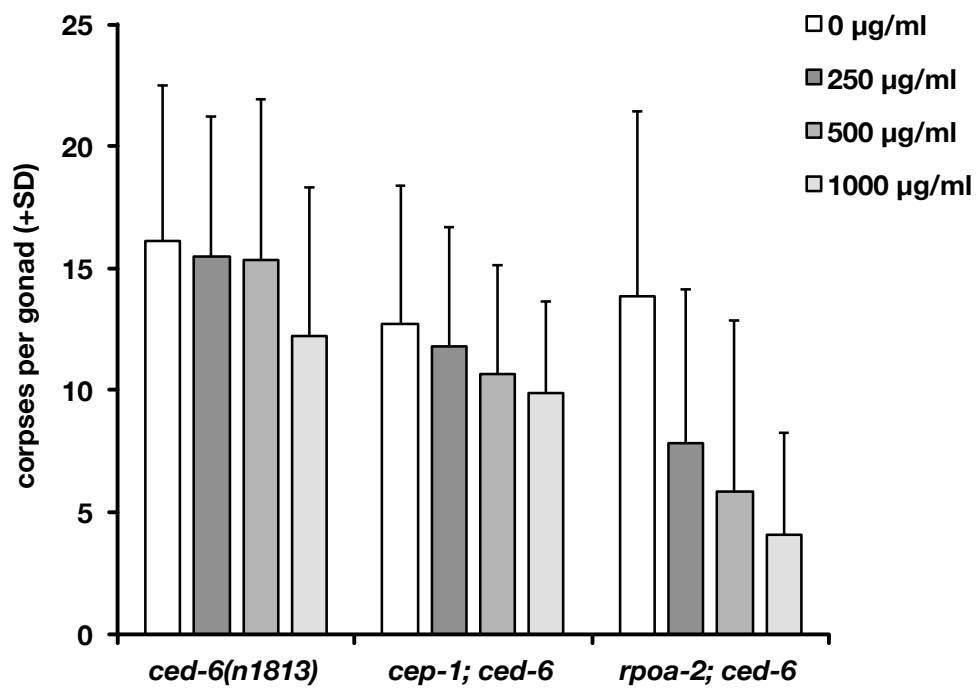

Supplement: Figure S12 — Translation-inhibition with cycloheximide (CHX) blocks irradiation-induced apoptosis and strongly affects animal health. A) Apoptotic germ cell corpses 18 hours after irradiation. Adult worms were transferred to freshly prepared CHX plates for 6 hours before irradiation and kept on the drug. The progeny were assessed at 2 and 4 days after treatment initiation for developmental delay or arrest. A significant reduction of corpses occurred in conditions that also had a strong effect on germ line integrity and animal growth. Average number of corpses and SD of at least 16 animals per condition. B) Accumulation of corpses in the engulfment mutant background ced-6(n1813). Young adult worms were transferred to freshly prepared CHX plates and exposed for 16 hours. Effect of CHX on constitutive cell death is weak at intermediate doses in the engulfment single mutant, but pronounced in combination with the rpoa-2(op259) mutation, indicating that constitutive cell death depends on new protein synthesis in this mutant. Average number of corpses and SD of at least 20 animals per condition. (PDF) [file pgen.1003943.s012.pdf]

**A**

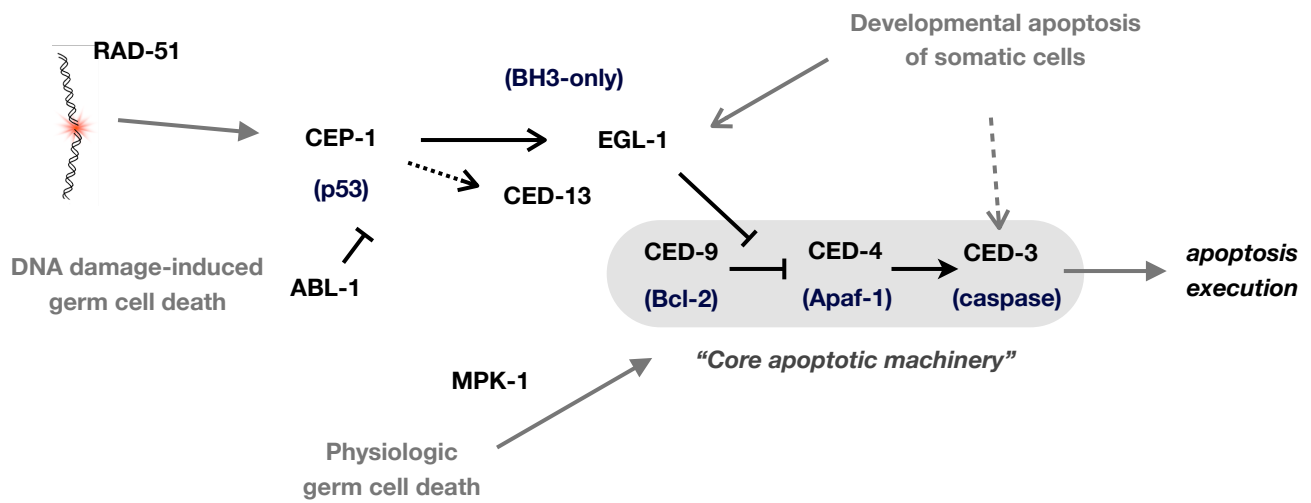

# B

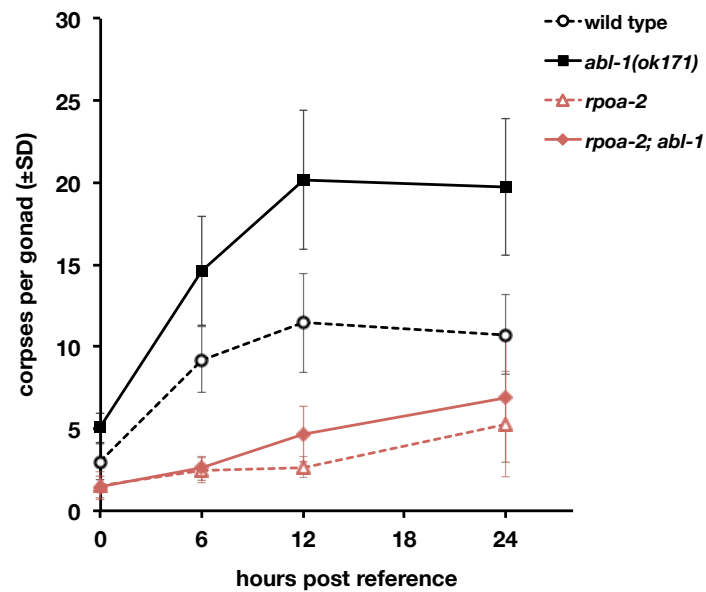

**C**

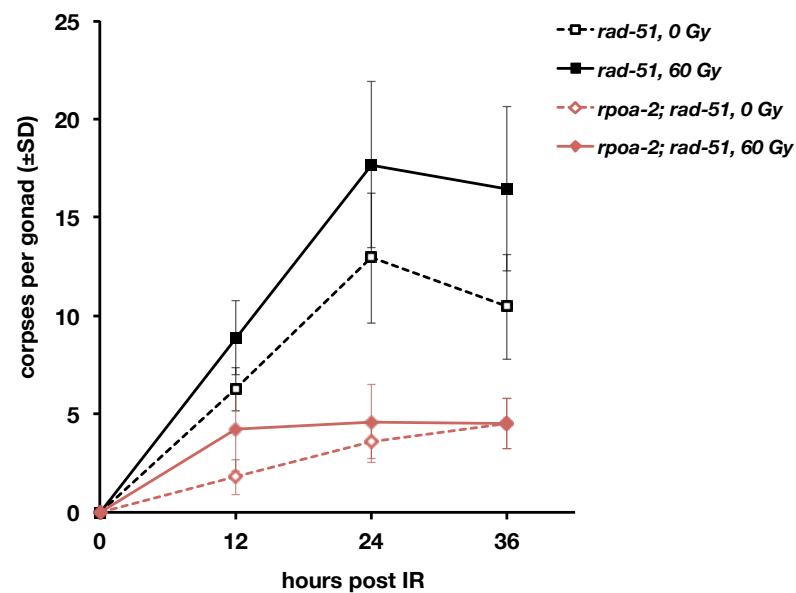

Supplement: Figure S13 — rpoa-2(op259) suppresses excessive apoptosis in rad-51(lf) and abl-1(lf) mutants. A) Current model of apoptosis induction in C. elegans. Two modes have classically been distinguished for the apoptotic death of germ cells, mainly by their dependence on cep-1 and egl-1. During somatic development, transcriptional regulation of EGL-1 is key for the lineage-specific induction of most cell deaths. EGL-1 physically disrupts the inhibitory binding of CED-9 to CED-4, which in turn serves as platform for CED-3 activation [9]. For DNA damage-induced germ cell death, EGL-1 and CED-13 are transcriptionally up-regulated by CEP-1/p53. By contrast, physiological germ cell death is largely CEP-1- and EGL-1-independent. abl-1(lf) and rad-51(lf) were shown to increase CEP-1 dependent cell death [10], [11]. B) Apoptotic response to IR irradiation (60 Gy) in the abl-1(ok171) kinase mutant background. Error bars, CI 95% of the mean number of germ cell corpses per gonad (n = 15). C) Baseline apoptosis (0 Gy, straight lines) and response to IR irradiation (60 Gy, dashed lines) in the rad-51(lg8701) DNA repair-mutant background. rad-51(lg8701) homozygous animals were derived from the balanced strains rad-51(lg8701)/nT1 and rpoa-2(op259); rad-51(lg8701)/nT1 and irradiated as young adults. Error bars, CI 95% of the mean number of germ cell corpses per gonad (n = 15). (PDF) [file pgen.1003943.s013.pdf]

**A**

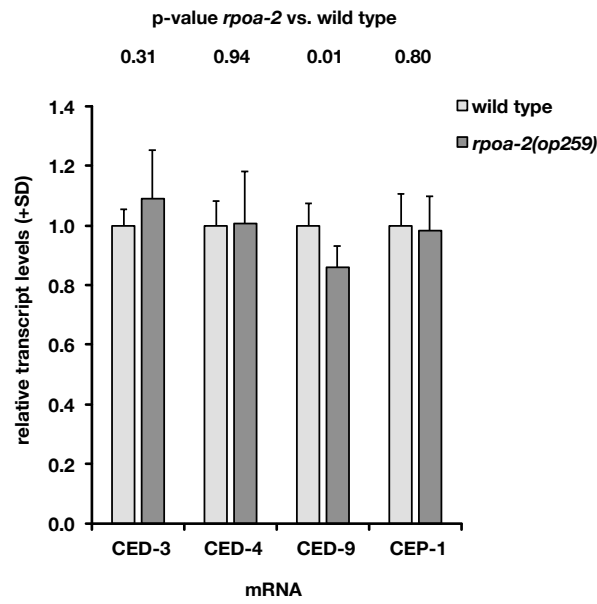

**B**

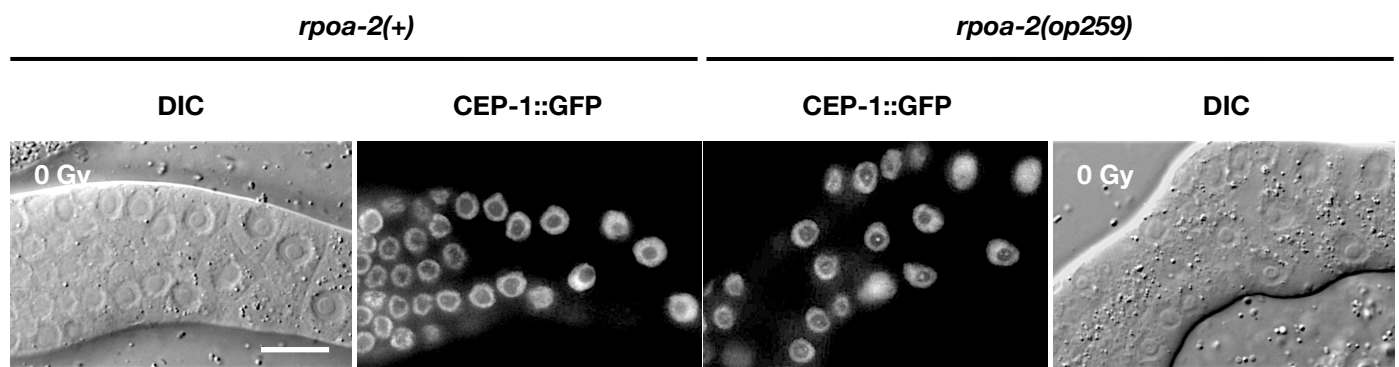

**C**

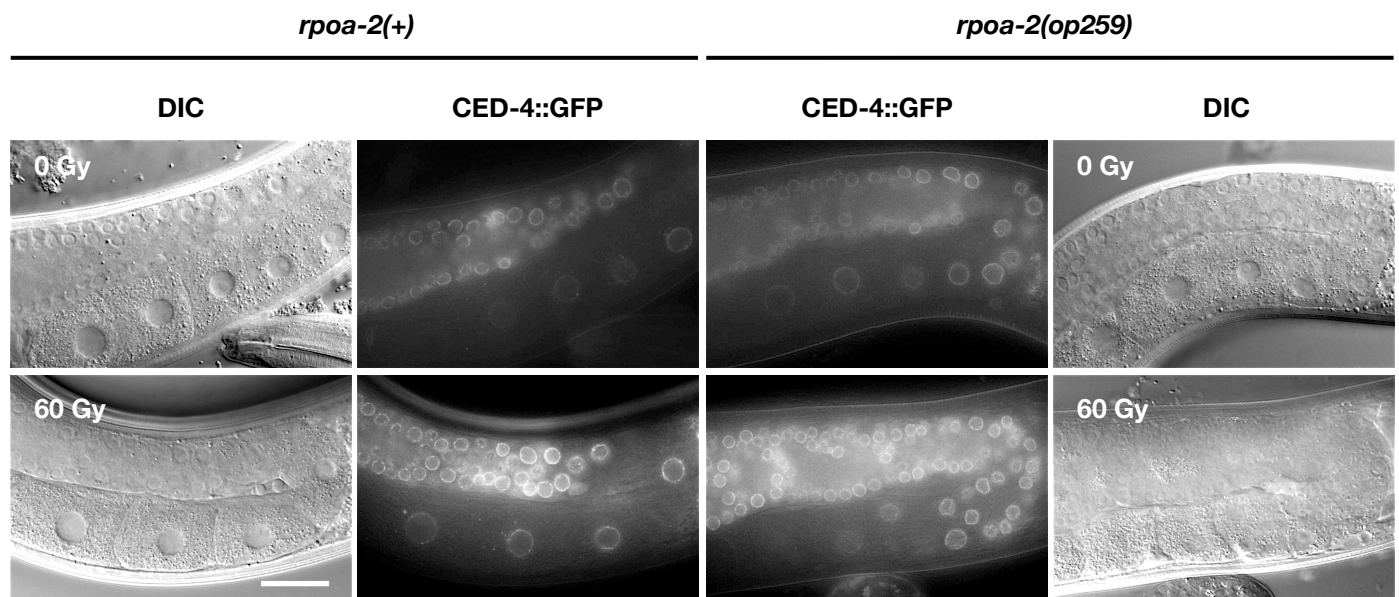

Supplement: Figure S14 — Expression of CEP-1 and core apoptotic factors is not significantly altered in rpoa-2(op259). A) qRT-PCR analysis of core apoptotic factors in whole-worm extracts. Average normalised levels of at least 7 independent samples per condition. Relative transcript levels in adult rpoa-2(op259) worms are very similar to wild type, except for CED-9 mRNA, which has a moderate but statistically significant decrease in rpoa-2(op259) (p-value of t-test indicated). B) CEP-1::GFP expression in germ cells of the late meiotic pachytene region. Gonads were extruded from adult worms on the second day of adulthood. Fluorescence pattern and intensity are similar between rpoa-2(op259) and wild-type gonads. Of note, the nucleoli in rpoa-2(op259) germ cells often have a more pronounced nucleolar dot (visible by DIC), that is positive for CEP-1::GFP (see also Fig. S8B). Size bar, 15 µm. C) CED-4::GFP expression (opIs219) in meiotic germ cells. Perinuclear signal intensity in the late meiotic pachytene region increases slightly upon irradiation. No obvious difference in signal intensity or pattern between wild type and mutant. Size bar, 20 µm. (PDF) [file pgen.1003943.s014.pdf]

**A**

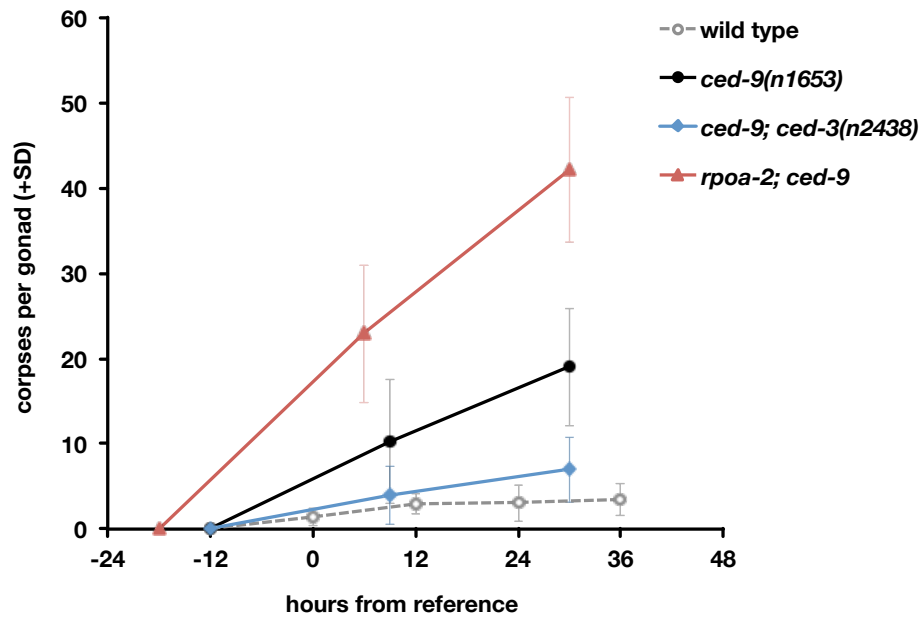

**B**

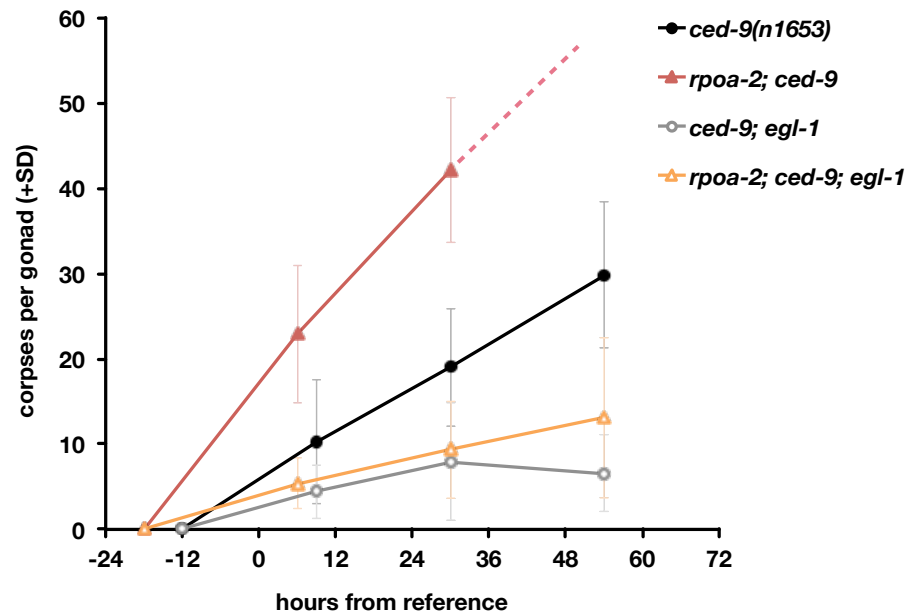

**C**

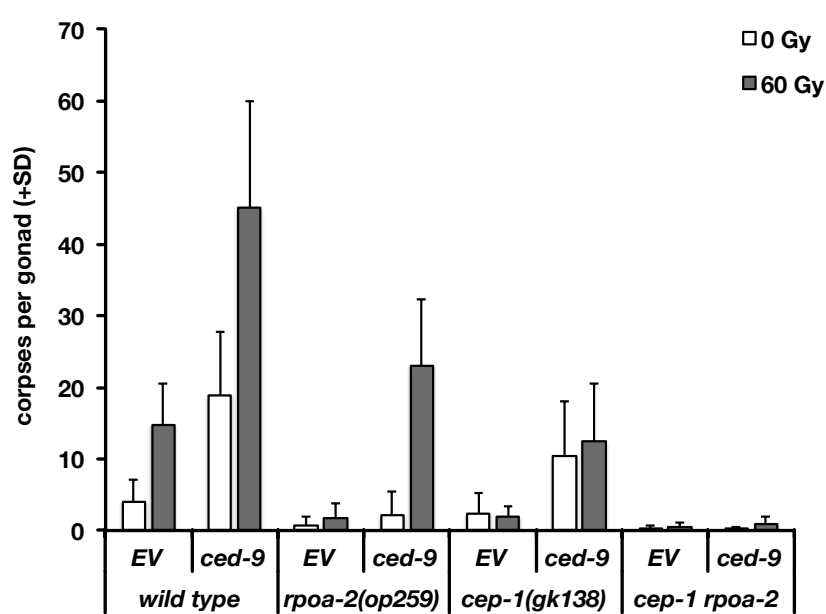

Supplement: Figure S15 — rpoa-2(op259) enhances the pro-apoptotic effect of the ced-9(n1653) mutant but suppresses the effect of ced-9(RNAi). A) Baseline apoptosis in the temperature-sensitive ced-9(n1653) and rpoa-2(op259); ced-9(n1653) mutants. The reduction-of-function mutation ced-3(n2438) reduces the high corpse number of ced-9(n1653) mutants, consistent with the known genetic epistasis. The rpoa-2(op259) mutation, however, accentuates the increased corpse number. Average number of corpses and SD of at least 40 animals per condition. B) When shifted from 15° to 20°C at the L4 stage, most of the rpoa-2(op259); ced-9(n1653) animals become sterile due to excessive apoptosis, which eventually affects all proximal germ cells in the course of adulthood (red dotted line). Excessive apoptosis is suppressed when egl-1 function is lost by the egl-1(n1084 n3082) allele. Average number of corpses and SD of at least 48 animals per condition. C) Germ cell apoptosis in animals treated with ced-9(RNAi) (starting at L1) and irradiated as young adults. ced-9(RNAi) does not significantly increase the number of corpses in rpoa-2(op259); however, knockdown of ced-9 strongly sensitises the mutant for irradiation-induced apoptosis. Average number of corpses at 24 hours after irradiation and SD of at least 40 animals per condition. (PDF) [file pgen.1003943.s015.pdf]

**A**

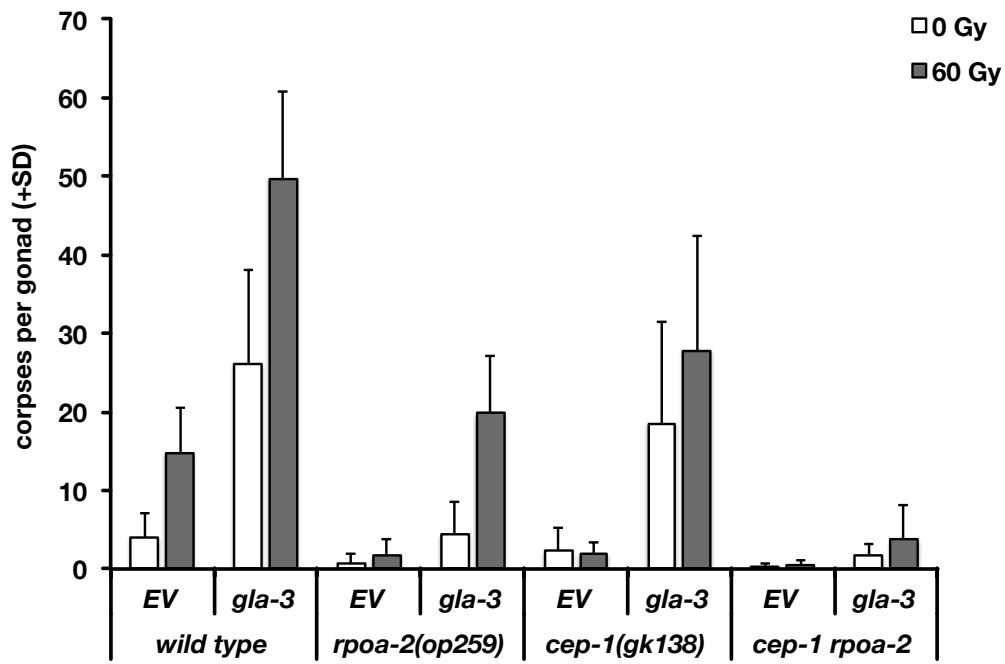

**B**

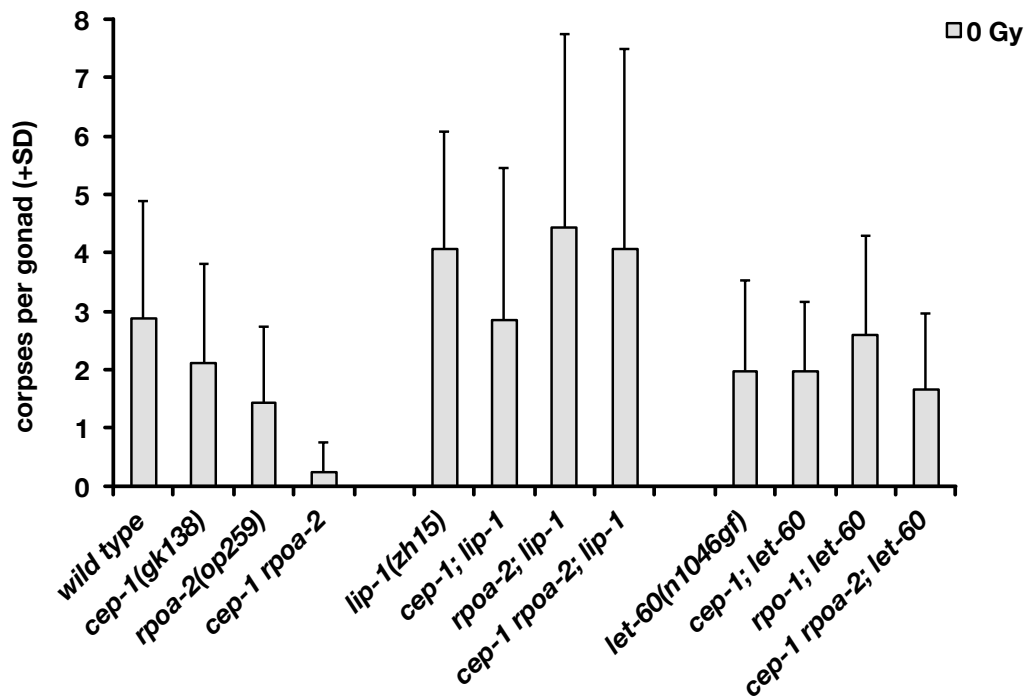

Supplement: Figure S16 — Knock-down of gla-3 sensitises for irradiation-induced apoptosis, which is antagonised by rpoa-2(op259). A) Germ cell apoptosis in animals treated with RNAi to gla-3 (starting at L1) and irradiated as young adults. gla-3(RNAi) does not only increase constitutive cell death in wild-type worms but also significantly increases the response to irradiation; this sensitisation is partly cep-1-independent. In rpoa-2(op259) mutants, gla-3(RNAi) does not strongly increase baseline apoptosis; however, knockdown of gla-3 strongly sensitises the mutant for irradiation-induced apoptosis. Average number of corpses at 24 hours after irradiation and SD of at least 40 animals per condition. B) Loss of lip-1 function or a gain-of-function mutation of let-60 restores baseline germ cell apoptosis in cep-1 rpoa-2. Average number of corpses and SD of at least 40 animals at 24 hours of adulthood. (PDF) [file pgen.1003943.s016.pdf]

**A**

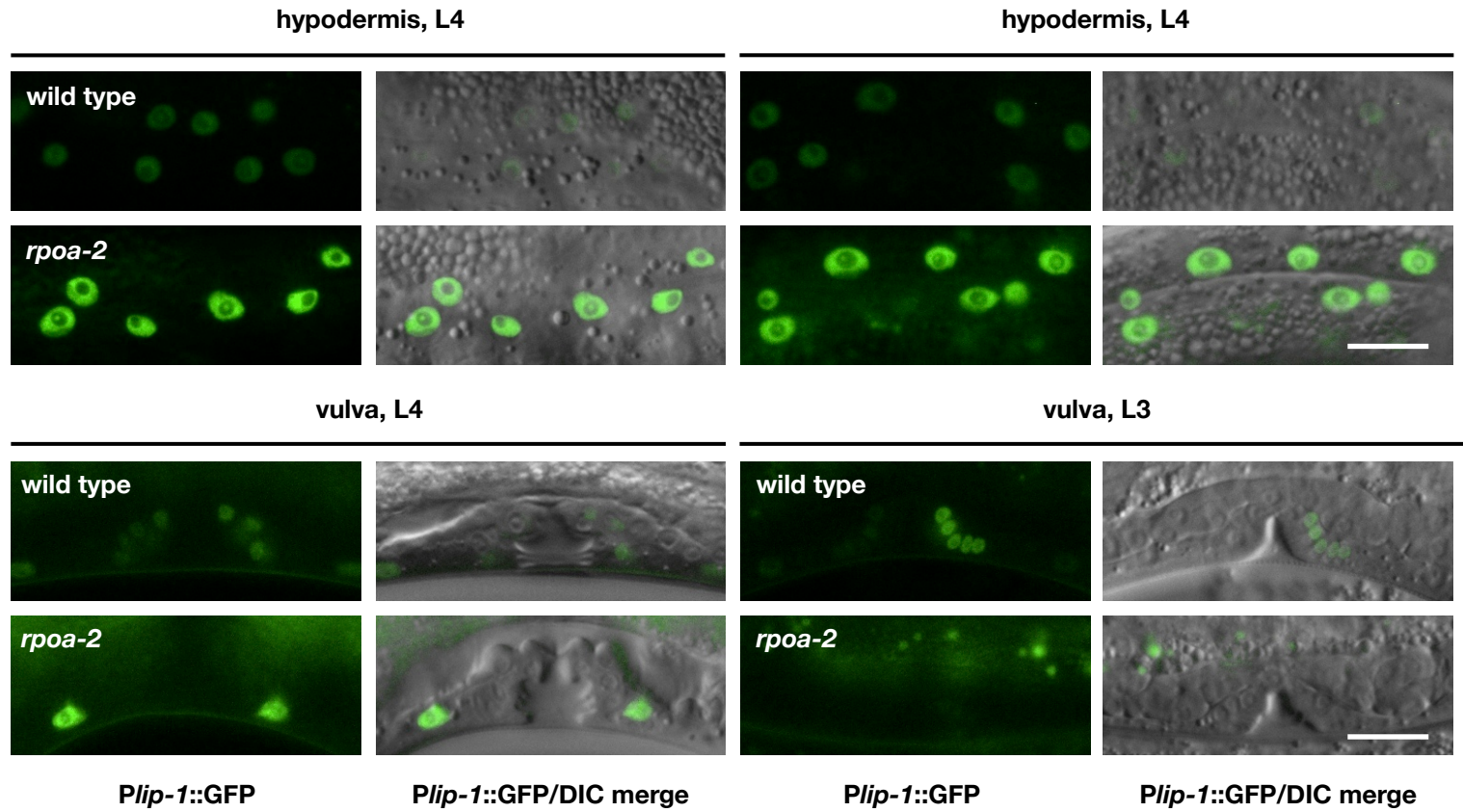

**B**

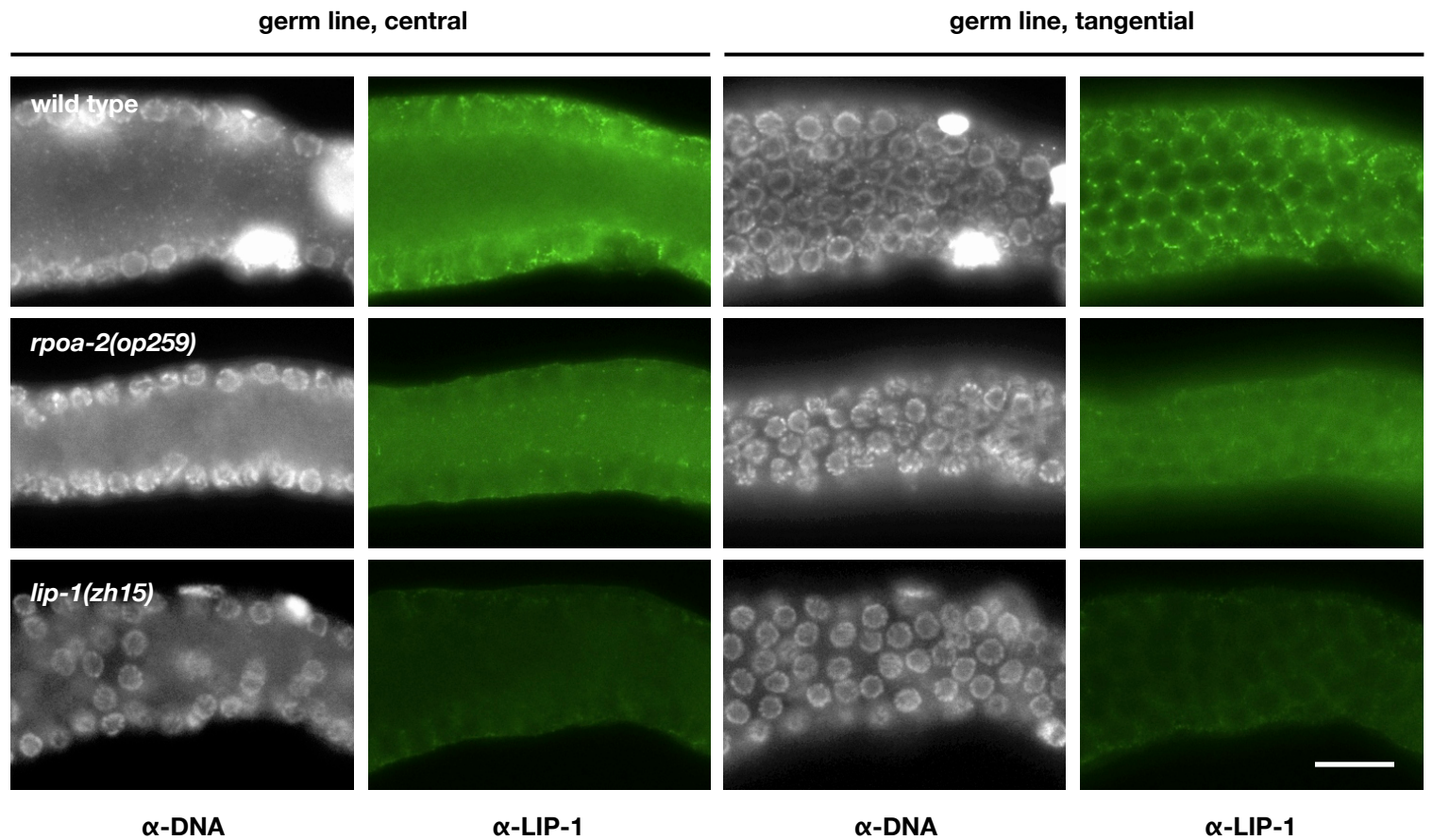

Supplement: Figure S17 — LIP-1 expression and localisation is altered in rpoa-2(op259) mutants. A) Expression of the transcriptional reporter zhIs4[Plip-1::GFP; unc-119(+)] in L3 and L4 stage larvae. Top panel, prominent fluorescence in hypodermal cells of rpoa-2(op259) animals. Size bar 15 µm. Second panel, LIP-1 expression is reduced in vulval cells of the mutant at different developmental stages; contrast with the stronger signal in hypodermal cells. The asymmetric expression in wild-type vulvae was consistently observed in multiple animals. The reporter is not expressed in the germ line. Size bar 15 µm. B) Immunostaining for LIP-1 at 18 hours after the onset of egg laying (see Text S1). The membrane-associated signal ubiquitously found in the gonads of wild-type worms is not found in the late pachytene region of rpoa-2(op259) mutant germ lines. Control staining with an anti-DNA antibody; same exposure settings in all three lines. Size bar 12 µm. (PDF) [file pgen.1003943.s017.pdf]

*rpoa-2(+)*

*rpoa-2(op259)*

*Pgst-4::GFP*

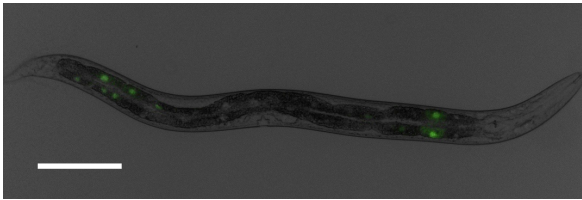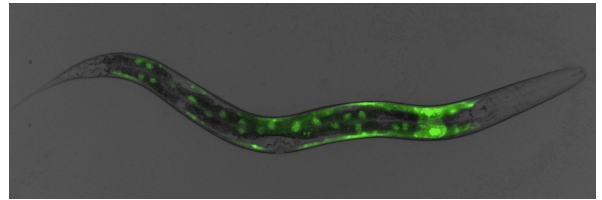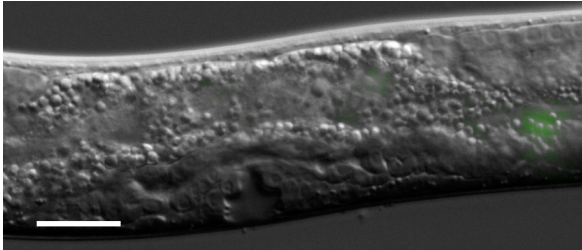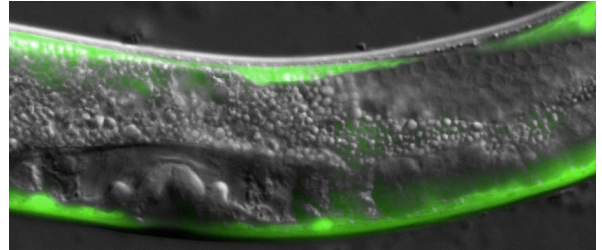

*Pnlp-29::GFP*

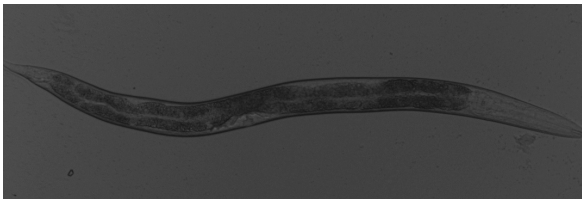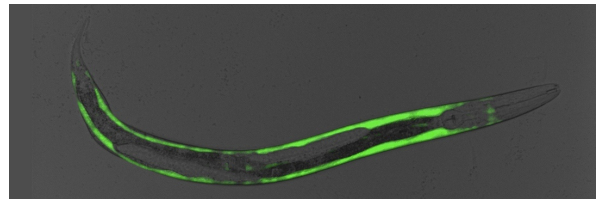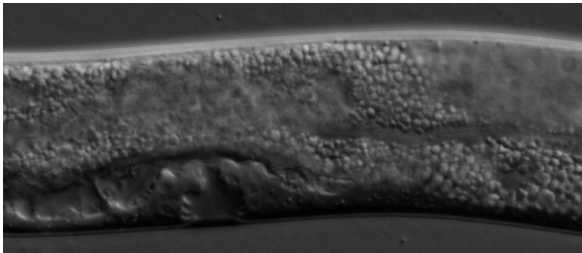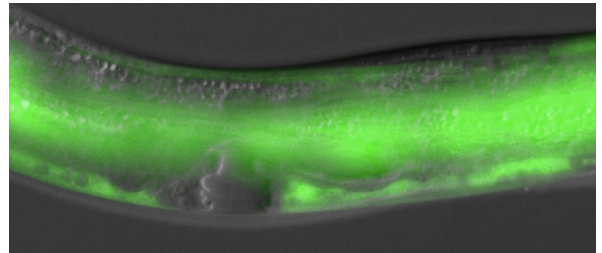

*Psod-3::GFP*

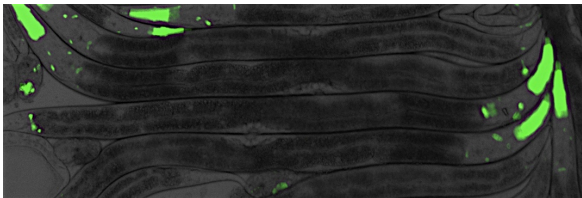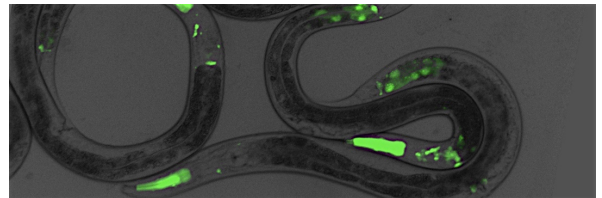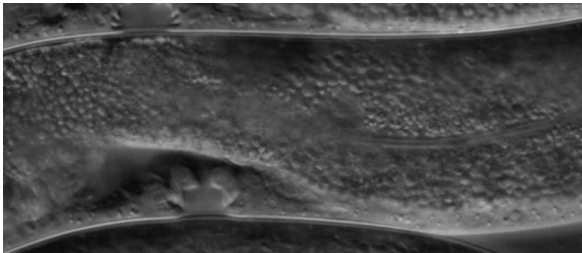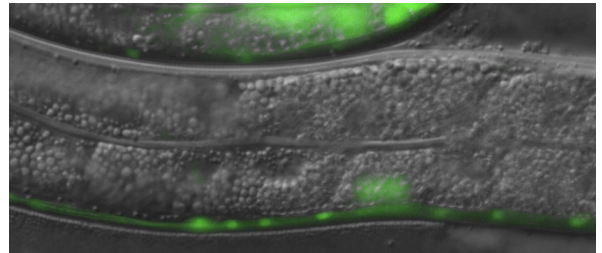

*Phsp-6::GFP*

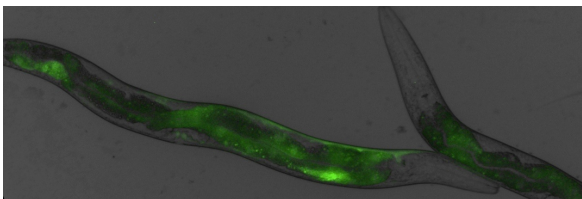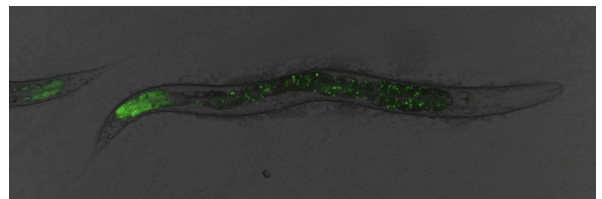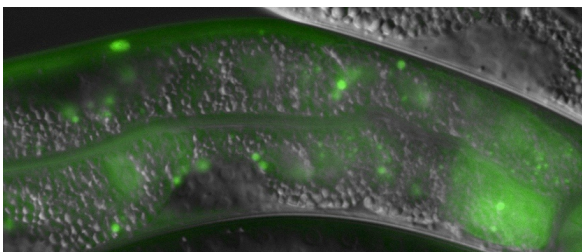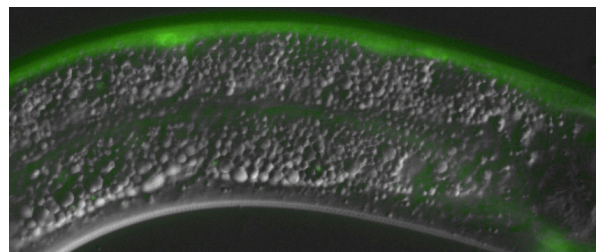

Supplement: Figure S18 — Multiple stress response factors are upregulated in rpoa-2(op259) mutants. Expression in L4 stage larvae of transcriptional reporters for GST-4 (dvIs19[Pgst-4::GFP::NLS]; oxidative stress), NLP-29 (frIs7[Pnlp-29::GFP+Pcol-12::DsRed]; infection, wounding, osmotic stress), SOD-3 (muIs84[Psod-3::GFP+rol-6]; oxidative stress), and HSP-6 (zcIs13[Phsp-6::GFP]; heat shock). Worms are oriented with the head to the right and ventral body part to the bottom. Overview (10× lens) and higher magnification (40×) exposures; size bar 120 µm or 20 µm, respectively. (PDF) [file pgen.1003943.s018.pdf]
