# Supplementary material for: Ribosome Synthesis and MAPK Activity Modulate Ionizing Radiation-Induced Germ Cell Apoptosis in Caenorhabditis elegans
Source: PLoS Genet. 2013 Nov 21;9(11):e1003943. doi: 10.1371/journal.pgen.1003943 (PMC3836707; doi:10.1371/journal.pgen.1003943)
Supplement: Figure S4 — Growth and lifespan of rpoa-2(op259) mutant animals. A) Reproductive cycles of wild-type and rpoa-2(op259) mutant worms grown at 15°C, 20°C, or 25°C, showing the duration of each developmental stage. Adult worms grown under standard conditions were bleached and the synchronised embryos were transferred to fresh plates and raised at the indicated temperature. Time points when the majority of the population had passed a developmental stage transit were recorded. At 20°C, rpoa-2(op259) animals have a delay mostly on account of an extended period as young adults (after moulting but prior to egg laying); the duration of young adulthood at 25°C is approximated by rare escaping animals from temperature sensitive sterility. B) Egg laying rate in the first 2 days of adulthood. Staged, well-fed animals were transferred in small groups to fresh plates and allowed to lay eggs for 3–6 hours. Eggs were then counted and the average number of eggs laid per animal per hour was calculated. Average of at least 36 animals and 3 plates per condition, SD of the weighted averages of the plates. The onset of egg laying is delayed to approximately 30 hours post L4 in rpoa-2(op259) animals. C) Life span is not extended in rpoa-2(op259), unlike in the translation initiation mutant ife-2. Adult worms were transferred onto fresh plates every two days so they would always be clearly distinguishable from the progeny. Death was defined as ceasing of pharyngeal pumping activity, of (head) movements and of reaction to touch. Bagging animals with internal hatching of larvae and animals drying on the dishes were censored. Kaplan-Meier survival curves were calculated using JMP 9 statistical software. The experiment was started with 120 young adult animals per strain. χ2 testing was in comparison to N2 wild-type worms. (PDF) [file pgen.1003943.s004.pdf]

Eberhard et al. 2013, Figure S4

A

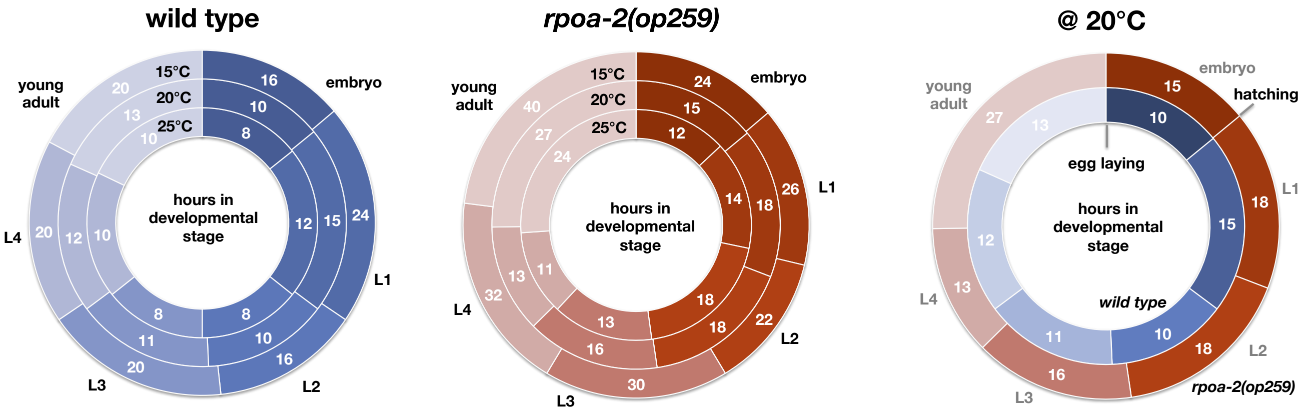

B

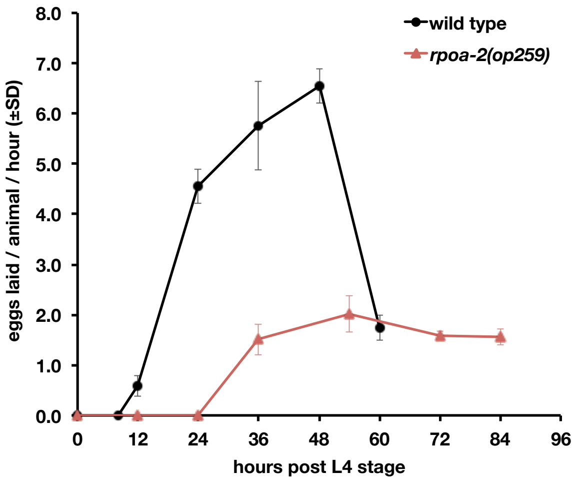

C

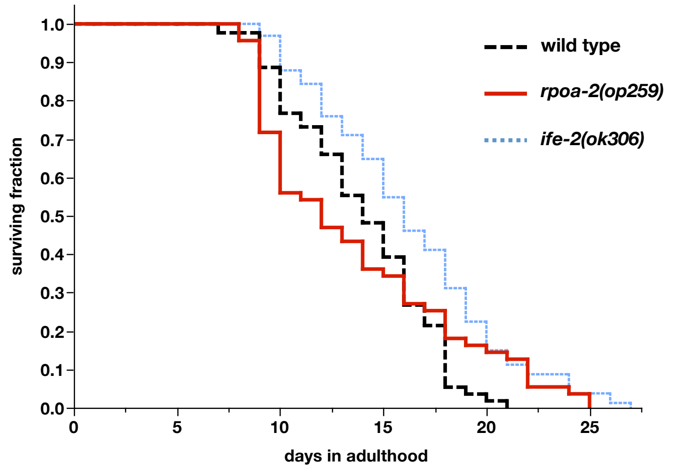

	wild type	*rpoa-2*	*ife-2*
# failed	58	57	82
# censored	63	63	38
Mean survival [d]	14.02	13.67	16.29
Std Error	0.44	0.66	0.48
Median survival [d]	14	12	16
Lower 95% confidence	13	10	15
Upper 95% confidence	16	14	18
χ^2 Wilcoxon (wild type)		0.171	0.0016
