# Supplementary material for: Ribosome Synthesis and MAPK Activity Modulate Ionizing Radiation-Induced Germ Cell Apoptosis in Caenorhabditis elegans
Source: PLoS Genet. 2013 Nov 21;9(11):e1003943. doi: 10.1371/journal.pgen.1003943 (PMC3836707; doi:10.1371/journal.pgen.1003943)
Supplement: Figure S5 — Germ cell proliferation and differentiation in rpoa-2(op259) mutants. A) Germ line organisation in DAPI stained whole worms (maximal intensity projection) 24 hours after the onset of egg laying. Distribution of distinct zones (defined by the characteristic chromatin patterns of germ cell nuclei) is indicated with coloured lines. B) Germ cell content of wild-type or mutant gonads at several time points with respect to the onset of egg laying (reference). Germ cells were classified individually according to their chromatin pattern. Although germ line maturation is delayed in rpoa-2(op259) mutants, a numerically similar germ cell composition as in wild type is eventually established. C) Data table to Fig. S5B. Average number of cells ± SD of 4 gonads per data point. Last row indicates average number ± SD of mitotic figures in 8 gonads. (PDF) [file pgen.1003943.s005.pdf]

A

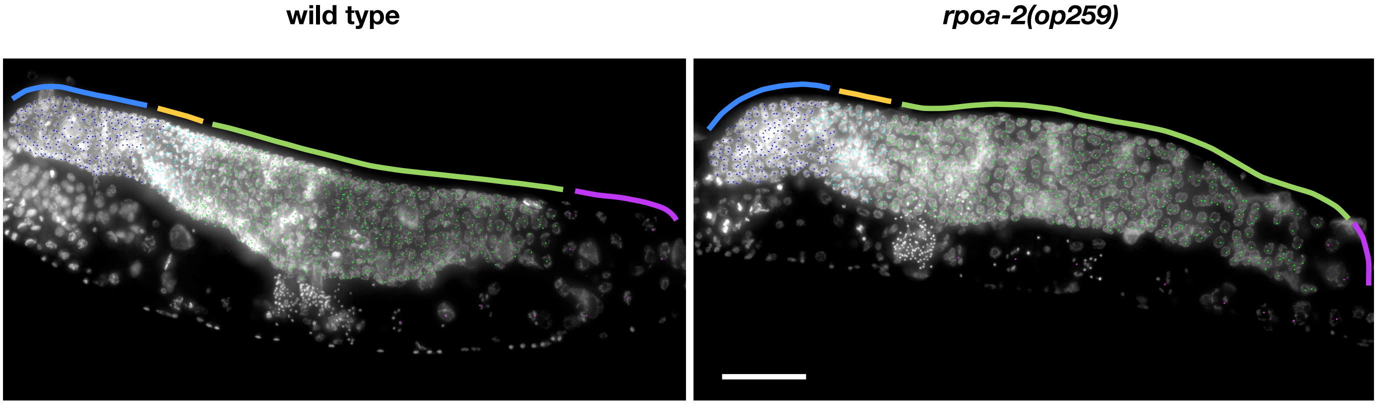

B

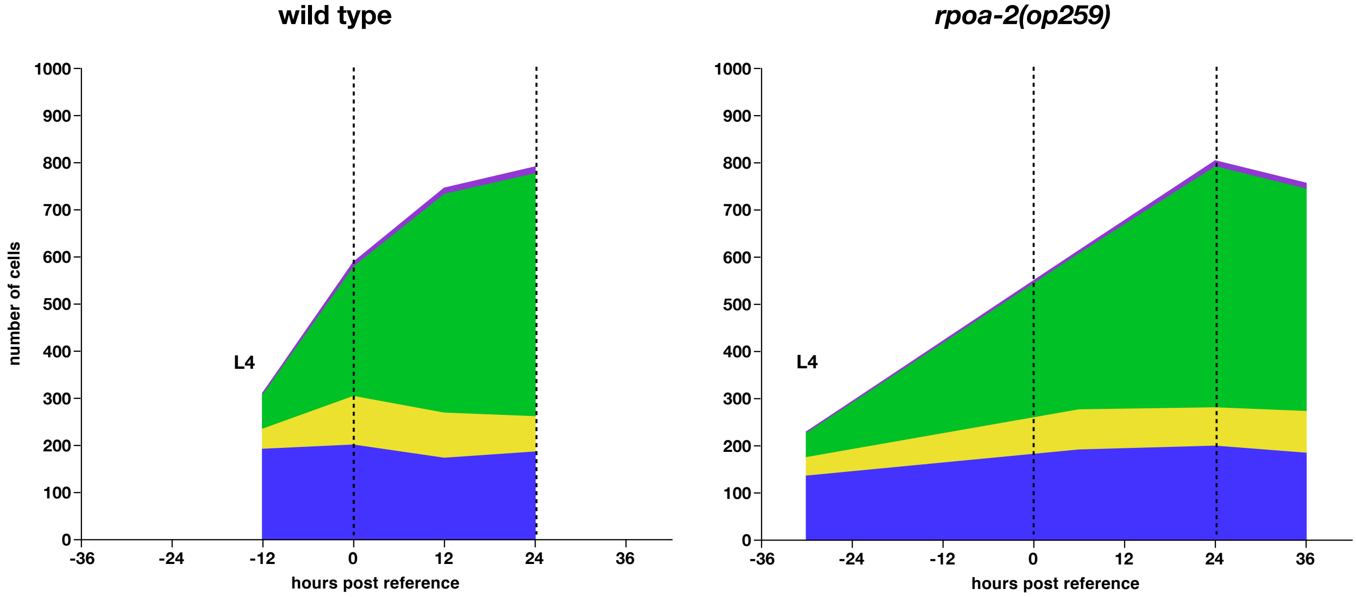

C

	wild type				rpoa-2(op259)			
	**L4**	**reference**	**12 h**	**24 h**	**L4**	**6h post ref**	**24 h**	**36 h**
**mitotic cells**	193 ± 5	202 ± 23	174 ± 16	187 ± 23	137 ± 39	192 ± 21	201 ± 18	186 ± 10
**transition zone**	43 ± 10	103 ± 33	96 ± 18	75 ± 25	39 ± 8	85 ± 11	81 ± 13	89 ± 9
**mid-late pachytene**	73 ± 21	275 ± 14	464 ± 114	516 ± 86	52 ± 34	331 ± 98	511 ± 17	472 ± 30
**oocytes**	0 ± 0	7 ± 1	11 ± 4	12 ± 3	0 ± 0	4 ± 1	10 ± 0	10 ± 1
**total**	309 ± 29	588 ± 55	745 ± 137	790 ± 58	228 ± 76	613 ± 83	803 ± 22	756 ± 40
**mitotic figures**	2.1 ± 1.1	4.4 ± 1.9	2.2 ± 1.2	0.6 ± 0.5	2.0 ± 1.7	1.4 ± 1.4	0.2 ± 0.4	0.2 ± 0.4
