# Supplementary material for: Ribosome Synthesis and MAPK Activity Modulate Ionizing Radiation-Induced Germ Cell Apoptosis in Caenorhabditis elegans
Source: PLoS Genet. 2013 Nov 21;9(11):e1003943. doi: 10.1371/journal.pgen.1003943 (PMC3836707; doi:10.1371/journal.pgen.1003943)
Supplement: Figure S7 — The Gogo phenotype in rpoa-2(op259) mutant animals. A) rpoa-2(op259) mutants are prone to develop distal oocytes (triangles in upper gonad arm) and associated corpses (arrowheads) in the distal arm within a field of meiotic germ cells, far distal to the gonad bend (right side of the picture), leading to a pattern of pachytene stage germ cells – oocyte(s) – pachytene stage germ cells – oocytes (Gogo). In the animal shown, the appearance of the first of the orthotopic oocytes (triangles in the lower gonad arm) is shifted towards the proximal end of the female gonad. Distal oocytes with condensed chromosome pairs (open triangles) are interspersed with nuclei that have a “spaghetti bowl” chromatin pattern characteristic of late meiotic pachytene cells (short arrows, inset), which distinguishes this abnormality from the Pro phenotype (Fig. S6B). One particularly sensitive condition for the Gogo phenotype is irradiation of rpoa-2(op259) worms fed on RNAi bacteria (condition shown). B) Schematic of the Pro and Gogo phenotypes found in a fraction of rpoa-2(op259) mutant gonads at sensitive conditions, indicating the topology of ectopic germ cell differentiation stages; colour code as in Fig. S5A. C) Distal oocytes in rpoa-2(op259) are strongly suppressed by loss of cep-1 function. They are not suppressed by inhibition of cell death with ced-3(RNAi); however, the associated corpses are lost, indicating that the latter are indeed apoptotic. RNAi treatment was started at L1 stage. Table indicates the fraction of gonads having developed a Gogo phenotype by 48 hours after irradiation (average of at least three experiments for rpoa-2(op259) and cep-1 rpoa-2(op259)) and the total number of gonads scored per condition; n.d., not determined. (PDF) [file pgen.1003943.s007.pdf]

A

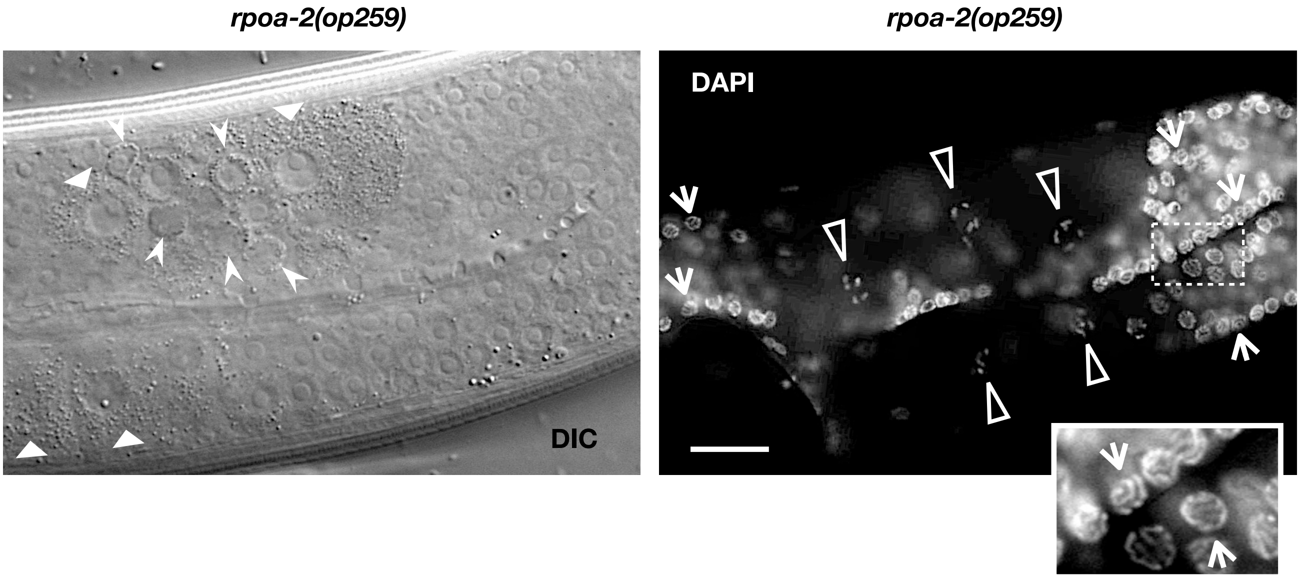

B

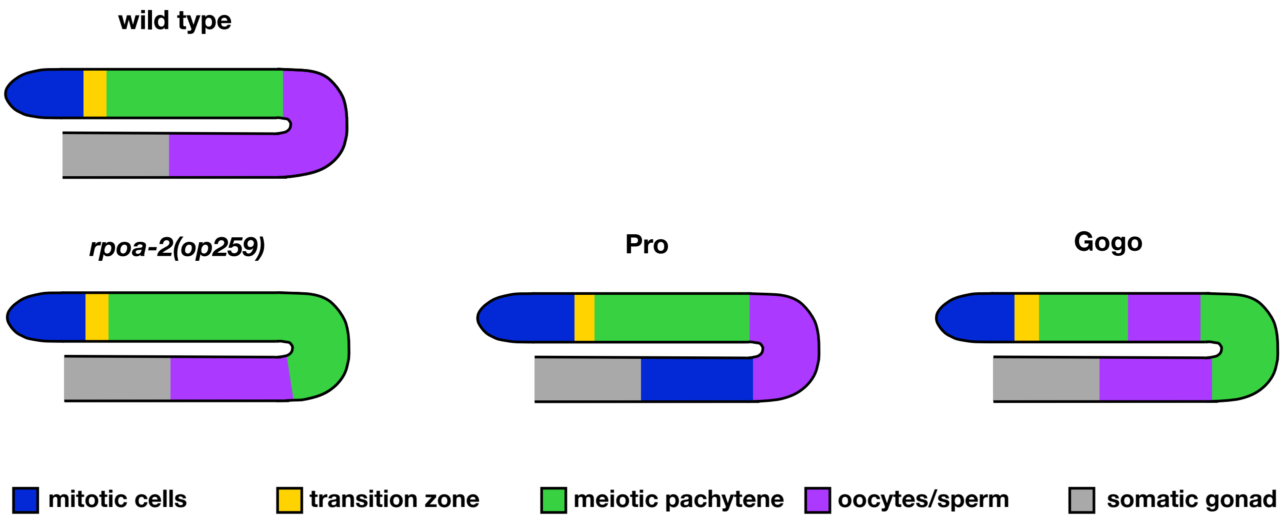

C

bacteria	OP50				*empty vector(RNAi)*				*ced-3(RNAi)*			
IR dose	0 Gy		60 Gy		0 Gy		60 Gy		0 Gy		60 Gy	
genotype	Gogo	n	Gogo	n	Gogo	n	Gogo	n	Gogo	n	Gogo	n
wild type	0%	140	0%	140	**0%**	140	**0%**	140	n.d.		n.d.	
rpoa-2(op259)	4%	90	6%	183	**7%**	246	**21%**	332	9%	22	25%	104
cep-1(gk138)	0%	50	0%	50	**0%**	50	**0%**	50	n.d.		n.d.	
cep-1 rpoa-2	4%	50	3%	40	**3%**	94	**7%**	110	n.d.		n.d.	
