# Supplementary material for: Ribosome Synthesis and MAPK Activity Modulate Ionizing Radiation-Induced Germ Cell Apoptosis in Caenorhabditis elegans
Source: PLoS Genet. 2013 Nov 21;9(11):e1003943. doi: 10.1371/journal.pgen.1003943 (PMC3836707; doi:10.1371/journal.pgen.1003943)
Supplement: Table S1 — Eukaryotic RNA polymerase I/II/III subunits.Common names (grey column) and the human and yeast names of the subunits of the three polymerases are indicated. Shared subunits are in blue (all three nuclear RNA polymerases), or green (Pol I and Pol III). In C. elegans, the homologs of the Pol II subunits had been defined and accordingly named rpb-x (except for the largest and the two accessory subunits; Wormbase WS200). Homologs of the Pol I and Pol III subunits were assembled from Wormbase database entries and from BLAST searches. For the accessory subunits of the core transcription apparatus of RNA pol I (A14, A43, A34.5), the C. elegans homologs remain to be identified (n.i.). “rpa-1” and “rpa-2” have already been attributed to other genes and cannot be used to name the RNA pol I (‘A’) subunits 1 and 2, hence the gene names rpoa-x for RNA pol I subunits. The table was mainly assembled from NCBI Homologene and literature searches [12], [13]. (PDF) [file pgen.1003943.s019.pdf]

Eberhard et al. 2013, Table S1

RNA polymerase I					RNA polymerase II					RNA polymerase III					Archeae
**common**	**human**	***S. cerevisiae***	***C. elegans***		**common**	**human**	***S. cerevisiae***	***C. elegans***		**common**	**human**	***S. cerevisiae***	***C. elegans***		
A190	POLR1A	RPA190	***rpoa-1***	Y48E1A.1	Rpb1	POLR2A	RPO21	***ama-1***	F36A4.7	C160	POLR3A	RPO31	***rpc-1***	C42D4.8	A
A135	POLR1B	RPA135	***rpoa-2***	F14B4.3	Rpb2	POLR2B	RPB2	***rpb-2***	C26E6.4	C128	POLR3B	RET1	***rpc-2***	F09F7.3	B
AC40	POLR1C	RPC40	***rpac-40***	H43I07.2	Rpb3	POLR2C	RPB3	***rpb-3***	C36B1.3	AC40	POLR1C	RPC40	***rpac-40***	H43I07.2	D
AC19	RPAC2		***rpac-19***	F58A4.9	Rpb11	POLR2J	RPB11	***rpb-11***	W01G7.3	AC19	RPAC2		***rpac-19***	F58A4.9	L
A12.2		RPA12	***rpoa-12***	C15H11.8	Rpb9	POLR2I	(RPB9)	***rpb-9***	Y97E10AR.5	C11	POLR3K	RPC11	***rpc-11***	Y77E11A.6	X
ABC27	POLR2E	RPB5	***rpb-5***	H27M09.2	Rpb5	POLR2E	RPB5	***rpb-5***	H27M09.2	ABC27	POLR2E	RPB5	***rpb-5***	H27M09.2	H
ABC23	POLR2F	RPO26	***rpb-6***	C06A1.5	Rpb6	POLR2F	RPO26	***rpb-6***	C06A1.5	ABC23	POLR2F	RPO26	***rpb-6***	C06A1.5	K
ABC14.5	POLR2H	RPB8	***rpb-8***	F26F4.11	Rpb8	POLR2H	RPB8	***rpb-8***	F26F4.11	ABC14.5	POLR2H	RPB8	***rpb-8***	F26F4.11	-
ABC10β	POLR2L	RPB10	***rpb-10***	Y37E3.3	Rpb10	POLR2L	RPB10	***rpb-10***	Y37E3.3	ABC10β	POLR2L	RPB10	***rpb-10***	Y37E3.3	N
ABC10α	POLR2K	RPB12	***rpb-12***	F23B2.13	Rpb12	POLR2K	RPB12	***rpb-12***	F23B2.13	ABC10α	POLR2K	RPB12	***rpb-12***	F23B2.13	P
A14	POLR1D	RPC19		n.i.	Rpb4	POLR2D	(RPB4)	***rpb-4***	F43E2.2	C4	POLR3D	RPC53?		n.i.	F
A43		RPA43		n.i.	Rpb7	POLR2G	RPB7	***rpb-7***	Y54E10BR.6	C25	POLR3H	RPC25	***rpc-25***	ZK856.10	E
A49	POLR1E	RPA49	***rpoa-49***	F23F1.9	RAP74	RAP74	TFG1		C01F1.1						
A34.5		RPA34		n.i.	RAP30	RAP30	TFG2		Y39B6A.36						
