# Supplementary material for: Ribosome Synthesis and MAPK Activity Modulate Ionizing Radiation-Induced Germ Cell Apoptosis in Caenorhabditis elegans
Source: PLoS Genet. 2013 Nov 21;9(11):e1003943. doi: 10.1371/journal.pgen.1003943 (PMC3836707; doi:10.1371/journal.pgen.1003943)
Supplement: Table S2 — RNAi knockdown of individual RNA polymerase subunits does not generally reduce irradiation-induced apoptosis. RNAi knockdown experiments and observed effects on fertility, development, and apoptosis. Synchronised L3 stage worms were transferred to RNAi bacteria, which often led to a visible effect by the time the worms reached adulthood. # experiments indicates the number of independent experiments with either the Ahringer (Ahr) and ORFeome (ORF) RNAi bacterial clones (where ‘0’ means no clones available). Egg laying was categorised as normal, reduced, or strongly reduced (few eggs). Hatching: + normal, most embryos hatch; (+) several non-hatched eggs; (−) mostly non-hatched eggs. Number of apoptotic corpses per gonad at 24 hours after irradiation are shown, as well as SD and total number of animals tested per condition. For knockdown of the ABC27, Rpb2 and Rpb3 homologs together with irradiation, germ line defects were so strong that scoring of apoptotic corpses was no longer possible (n.d.). Consistently, knockdown of rpb-12, of rpb-4, of F23F1.9 (A49), of Y77E11A.6 (Rpb9 paralog C11), or of Y39B6A.36 (RAP30) did not strongly affect viability or reproduction of the treated parental generation or their F1 progeny. Shared subunits between Pol I and Pol III are indicated with a green bar, and between all three polymerases with a blue bar. (PDF) [file pgen.1003943.s020.pdf]

Eberhard et al. 2013, Table S2

POLR subunit	*C. elegans* target gene		# experiments Ahr	ORF	egg laying	hatching	larval development	Apoptosis, 0 Gy mean	SD (n)	Apoptosis, 60 Gy mean	SD (n)
	none	empty vector			normal	+	normal	3.56	±2.84 (88)	16.90	±6.48 (88)
**A190**	*rpoa-1*	Y48E1A.1	0	0							
**A135**	*rpoa-2*	F14B4.3	6	1	few eggs	+	larval arrest, or arrest at size of early stages	1.94	±1.69 (36)	5.31	±4.90 (36)
**AC40**	*rpac-40*	H43I07.2	7	0	few eggs	+	delayed growth / variably penetrant larval arrest	3.67	±2.51 (36)	15.53	±7.37 (36)
**AC19**	*rpac-19*	F58A4.9	1	0	delayed	+	delayed growth	3.70	±2.30 (20)	11.15	±6.85 (20)
**A12.2**	*rpoa-12*	C15H11.8	6	1	delayed	+	normal to slightly delayed	3.83	±1.95 (36)	16.19	±6.79 (36)
**ABC27**	*rpb-5*	H27M09.2	0	1	reduced	none	no larvae	3.06	±1.81 (16)	n.d.	
**ABC23**	*rpb-6*	C06A1.5	6	1	normal	+	slightly delayed, some with germline defects	4.00	±3.36 (36)	12.81	±6.46 (36)
**ABC14.5**	*rpb-8*	F26F4.11	6	0	normal	(-)	delayed, sterile	3.25	±2.02 (20)	14.33	±5.79 (6)
**ABC10β**	*rpb-10*	Y37E3.3	0	0							
**ABC10α**	*rpb-12*	F23B2.13	6	1	(delayed)	+	normal	2.89	±2.21 (36)	16.92	±8.19 (36)
**A14**		n.i.									
**A43**		n.i.									
**A49**	*rpoa-49*	F23F1.9	3	0	normal	+	normal	3.19	±2.04 (32)	17.53	±4.86 (32)
**A34.5**		n.i.									
**Rpb1**	*ama-1*	F36A4.7	3	0	few eggs	(-)	delayed, arrest	2.21	±1.83 (33)	6.43	±5.59 (56)
**Rpb2**	*rpb-2*	C26E6.4	2	0	few eggs	(-)	early arrest	3.81	±3.04 (36)	n.d.	
**Rpb3**	*rpb-3*	C36B1.3	1	1	few eggs	(-)	early arrest	3.03	±2.56 (32)	n.d.	
**Rpb11**	*rpb-11*	W01G7.3	6	1	normal	(+)	slightly delayed, sterile	3.94	±2.60 (36)	16.25	±6.04 (36)
**Rpb9**	*rpb-9*	Y97E10AR.5	0	0							
**Rpb4**	*rpb-4*	F43E2.2	1	1	normal	+	normal	4.28	±2.37 (36)	14.58	±5.12 (36)
**Rpb7**	*rpb-7*	Y54E10BR.6	1	1	normal	(-)	early arrest	3.26	±2.27 (31)	12.44	±6.66 (32)
**RAP74**		C01F1.1	0	0							
**RAP30**		Y39B6A.36	1	0	normal	+	normal	2.00	±1.86 (16)	19.56	±5.06 (16)
**C160**	*rpc-1*	C42D4.8	1	1	reduced	+	early arrest	2.43	±1.79 (35)	14.44	±8.63 (36)
**C128**	*rpc-2*	F09F7.3	1	1	reduced	+	slightly delayed	3.22	±1.93 (36)	12.47	±6.50 (36)
**C11**	*rpc-11*	Y77E11A.6	1	1	normal	+	normal	5.03	±3.67 (36)	17.11	±7.09 (36)
**C4**		n.i.									
**C25**	*rpc-25*	ZK856.10	1	1	reduced	+	slightly delayed	4.19	±1.98 (36)	17.78	±6.90 (36)
