# Supplementary material for: Ribosome Synthesis and MAPK Activity Modulate Ionizing Radiation-Induced Germ Cell Apoptosis in Caenorhabditis elegans
Source: PLoS Genet. 2013 Nov 21;9(11):e1003943. doi: 10.1371/journal.pgen.1003943 (PMC3836707; doi:10.1371/journal.pgen.1003943)
Supplement: Table S3 — Interfering with ribosome synthesis abolishes IR-induced apoptosis. Knockdown of various rRNA processing factors and of ribosomal proteins reduces apoptotic response to irradiation in the germ line. Synchronised L3 stage worms were transferred to RNAi bacteria, which often led to a visible effect by the time the worms reached adulthood. For each RNAi bacterial clone (Gene sequence identifier and gene name where available), the source is indicated as Ahringer library (Ahr) or ORFeome RNAi library (ORF). RNAi target genes are arranged according to an approximate chain of action on ribosomal small subunit (SSU) or large subunit (LSU) synthesis, or ribosome degradation. fib-1 is a major nucleolar protein; rrbs-1 is important for 5S incorporation into the large subunit, eri-1 was shown to be involved in 5.8S rRNA processing. Arbitrarily, rpl-1, rpl-2, rps-1, rps-2 were selected for testing ribosomal proteins (RPs). P-P indicates the factors for which a physical protein interaction with RPOA-2 has been predicted (Wormbase WS200). Data represent average number of corpses per gonad 24 hours after irradiation, ±SD, and the total number of animals scored per condition; n.d., not determined. For opIs110; unc-119(ed3), corpses were scored by DIC and independently by the number of Actin::YFP halos forming from sheath cell protrusions around corpses (reporter opIs110[Plim-7::act-5::yfp; unc-119(+)]). (PDF) [file pgen.1003943.s021.pdf]

RNAi target	Gene	Library	Yeast	Processing	P-P	no IR		60 Gy		no IR, YFP halos		60 Gy, YFP halos	
**wild type**						**mean**	**SD (n)**	**mean**	**SD (n)**	**mean**	**SD (n)**	**mean**	**SD (n)**
EV control	*(none)*					3.64	±2.55 (148)	16.35	±6.07 (212)				
F14B4.3	*rpoa-2*	ORF	Rpa135			2.98	±2.20 (52)	7.25	±4.23 (52)				
C48D1.2	*ced-3*	ORF				0.05	±0.22 (20)	0.00	±0.00 (36)				
T01C3.7	*fib-1*	Ahr	NOP1	nucleolar	+	2.00	±1.46 (16)	6.60	±8.09 (20)				
E02H1.1		ORF	DIM1	SSU		1.25	±1.00 (16)	12.75	±4.66 (20)				
ZK795.3		ORF	IMP4	SSU		2.44	±1.90 (16)	11.15	±4.79 (20)				
F57B9.5	*byn-1*	ORF	ENP1	SSU		1.19	±1.55 (16)	7.90	±5.61 (16)				
C06E1.10	*rha-2*	Ahr	ECM16	SSU		1.88	±1.05 (16)	10.20	±4.17 (20)				
F56D2.6		ORF	PRP43	both		2.19	±1.67 (16)	10.25	±6.56 (20)				
K08F4.2		Ahr	G3BP	degrad.?		2.17	±1.60 (16)	14.91	±4.20 (16)				
opIs110;unc-119(ed3)													
EV control	*(none)*					2.15	±1.33 (52)	11.63	±5.32 (76)	4.63	±3.59 (52)	11.66	±1.55 (62)
F14B4.3	*rpoa-2*	ORF	Rpa135			n.d.		3.38	±1.45 (16)	n.d.		6.00	±1.09 (16)
C48D1.2	*ced-3*	ORF				0.02	±0.14 (52)	0.14	±0.83 (76)	0.33	±0.86 (48)	0.07	±0.07 (68)
C15H11.9	*rrbs-1*	ORF	RRS1	5S incorp.		0.90	±0.88 (10)	2.86	±2.92 (56)	3.75	±3.06 (8)	4.73	±0.57 (48)
R13A5.12	*lpd-7*	ORF	NOP7	LSU	+	1.06	±1.18 (16)	1.90	±1.97 (20)	1.81	±1.60 (16)	3.75	±1.04 (16)
C18A3.3		ORF	EBP2	LSU	+	1.25	±1.29 (16)	1.75	±1.68 (20)	2.63	±2.45 (16)	2.75	±0.87 (16)
Y48B6A.1		ORF	ERB1	LSU	+	1.25	±1.13 (16)	2.35	±1.63 (20)	3.71	±2.46 (14)	5.31	±1.48 (16)
C47B2.5	*eif-6*	ORF	TIF6	LSU	+	0.75	±1.06 (16)	3.55	±3.00 (20)	3.06	±1.81 (16)	5.63	±1.07 (16)
T07A9.5	*eri-1*	ORF	ERI1	5.8S proc.		2.00	±1.37 (16)	10.20	±5.12 (20)	9.69	±5.42 (16)	12.56	±2.63 (16)
Y48A6C.4		ORF	RIX1	LSU	+	0.56	±0.73 (16)	6.85	±4.44 (20)	5.13	±3.88 (16)	5.94	±0.92 (16)
C37H5.5		Ahr	NOC3	LSU		1.25	±1.34 (16)	8.75	±4.53 (20)	8.00	±3.41 (16)	8.31	±2.44 (16)
W07E6.2		ORF	YCR072	LSU	+	n.d.		1.81	±1.11 (16)	n.d.		6.31	±1.17 (16)
Y71F9AL.13	*rpl-1*	Ahr		RP		n.d.		1.13	±0.89 (16)	n.d.		3.81	±1.32 (16)
B0250.3	*rpl-2*	ORF		RP		1.50	±1.55 (16)	1.85	±2.39 (20)	2.75	±2.62 (16)	2.56	±0.76 (16)
F56F3.5	*rps-1*	ORF		RP		n.d.		0.81	±0.83 (16)	n.d.		3.13	±0.89 (16)
C49H3.11	*rps-2*	ORF		RP		0.63	±2.00 (16)	0.95	±1.19 (20)	3.69	±3.46 (16)	3.06	±0.99 (16)
