# Supplementary material for: Ribosome Synthesis and MAPK Activity Modulate Ionizing Radiation-Induced Germ Cell Apoptosis in Caenorhabditis elegans
Source: PLoS Genet. 2013 Nov 21;9(11):e1003943. doi: 10.1371/journal.pgen.1003943 (PMC3836707; doi:10.1371/journal.pgen.1003943)
Supplement: Table S4 — Primers used in this study. Primer sequences with internal numbering (ID) and names. (PDF) [file pgen.1003943.s022.pdf]

Eberhard et al. 2013, Table S4

	ID	Primer Name	Sequence	Remarks
***rpoa-2* genotyping**				
op259	062	pm_RPA2wtR_rv	CGTTGTCGACATTCCGCGag	*rpo-1(wt)* specific
	063	pm_op259aR_rv	CGTTGTCGACATTCCGCGaa	*op259* specific
	033	sg_RPA2-05_fw	CTCGCCAACCCCTAAATCTTTTAT	reverse to 062/063
ok1970	253	dm_ok1970_fw	CATTAGAGCCATCGGTTCTCGT	
	254	dm_ok1970_rv	CTGGTCCAACGGTTTTGATTTCTT	
**YFP::RPOA-2 cloning**				
rpoa-2 promoter	021	tt_RPA2-pLge_fw	ctatcctgCAGGACGTTAAGTGCAACAAATGTGCTCA	
	050	tt_RPA2-pr_rv2	actatggcgCGCCTGAAAACTTGAAAGTTTAAATGAAT	
YFP	053	tt_Asc1(5)yfp_fw	actatggcgcgccaggcgATGAGTAAAGGAGAAGAACTTTTCAC	
	054	tt_yfp(G)Fse1_rv	atttaaggccggccccGTATAGTTCATCCATGCCATGTGTA	
rpoa-2 gene	051	tt_RPA2-ge_fw2	atttaaggccggcctcATGGACTGCGACATAGCGTCGTATCAT	
	052	tt_RPA2-ge_rv2	ccaaccttaaTTAACTTCCAGTTACTTTGGATGGATGTTC	
rpoa-2 3'UTR	019	tt_RPA2-3U_fw	cgcgttaattaaggTTTCATTATTTTTTCAACATTTCCTTCA	
	020	tt_RPA2-3U_rv	ctatgggcCCGAGGTTATTGAAGATCACTCAGACG	
**rRNA probes**				
5S	071	rp_rRNA-5S_fw	GCTTACGACCATATCACGTTGAAT	
	072	rp_rRNA-5S_rv	AGCTTACAACATCCAGGATTCCC	
5.8S	073	rp_rRNA-58S_fw	CTAGCTTCAGCGATGGATCGGT	
	074	rp_rRNA-58S_rv	CAACCCTGAACCAGACGTACCA	
26S-1	075	rp_rRNA-26S1_fw	GTAACGGCGAGTGAAACGGGA	
	076	rp_rRNA-26S1_rv	GTCCTTTGCAACTTTCCCTCACG	
26S-2	077	rp_rRNA-26S2_fw	CGAGAGGAACAGCGGGTTCAAA	
	078	rp_rRNA-26S2_rv	GACCAAGAGACCAAGTCGTATGC	
18S	079	rp_rRNA-18S_fw	CACGAGATTGAGCGATAACAGGTC	
	080	rp_rRNA-18S_rv	CGAAGTCGTTAAACCTCGAAGCG	
its1	081	rp_rRNAits1_fw	GATGCTCGACTGGCTTCACG	
	082	rp_rRNAits1_rv	CCGAACCACGATCATCAAGACTAT	
its2	083	rp_rRNAits2_fw	CTCAATGCCTTAGGCTTCTCTTCG	
	084	rp_rRNAits2_rv	GACGAATCCCAGTATTCGAAAGGAG	
ets1	085	rp_rRNAets1_fw	CACACTCCTATATGTGTCAGGG	
	086	rp_rRNAets1_rv	GACCAATACCGCAACATCATTAGT	
**DIG-oligo probes**				
26S-short	206	do_26S-01c_rv	TACCCGCGCTTACTCGAATTACTA	DIG_26S-01c
	209	do_26S-04c_rv	CGAATAAGTAAAGAGTCGATGAAAGTG	DIG_26S-04c
	212	do_26S-07c_rv	CTGTGGTAACTTTTCTGACACCTC	DIG_26S-07c
**26S-short ends**				
RT	076	rp_rRNA-26S1_rv	GTCCTTTGCAACTTTCCCTCACG	primer_076
PCR / seq	191	do_26S-01_fw	TAGTAATTCGAGTAAGCGCGGGTA	primer_191
RNA adaptor	318	RNA_SRA5Adpt	GUUCAGAGUUCUACAGU	5' end ligation
	319	RNA_SRA3Adpt	P-UCGUAUGCCGUCUUCUGCUUGU-idT	3' end ligation
RT / PCR / seq	320	rc_RT-GX1	CAAGCAGAAGACGGCATACGA	complementary to 319
PCR / seq	321	rc_PCR_fw	GTTCAGAGTTCTACAGTCCGA	analogous to 318
	125	qP_rRNA-26S1_fw	GCTTGAAAGTGCAGCCTAAAGT	26S internal
	214	do_26S-09c_rv	CATTTTCAGGGCTAGTTGATTCGG	26S internal
	199	do_26S-09_fw	CCGAATCAACTAGCCCTGAAAATG	26S internal
	218	do_26S-13c_rv	ATCTAGTTTGCCGACTTCCCTTAC	26S internal
	201	do_26S-11_fw	CTAACTGAGATGCAAAGATTGTGTTC	26S internal
	060	pr_rDNA-26S_rv	ACCCGCGCTTACTCGAATTACTACGAT	26S internal

	ID	Primer Name	Sequence	Remarks
**qRT-PCR of rRNA**				
pre-rRNA	129	qP_rRNA-5ets_fw	CTGTGTTGGTGATGGTAGTGTG	
	130	qP_rRNA-5ets_rv	CATTGCTAGAATCATTCGGGTGT	
	139	qP_rR18S-i1_fw	GAACCGGGTAAAAGTCGTAACA	
	140	qP_rR18S-i1_rv	CCAGTCGAGCATCTTAAGGTTT	
	135	qP_rRNA-its1_fw	GGCTATATGCGTCTAGGCTTCT	
	136	qP_rRNA-its1_rv	CGAACCACGATCATCAAGACTA	
	143	qP_rR5.8S-i2_fw	AATTGCAGACGCTTAGAGTGGT	
	144	qP_rR5.8S-i2_rv	GAAGAGAAGCCTAAGGCATTGA	
	145	qP_rRi2-26S_fw	ACGGTGCGTGTCTTGCTAAT	
	146	qP_rRi2-26S_rv	TTCCTCCGCTAAATGATATGCTT	
rRNA	121	qP_rRNA-18S2_fw	TATTGCCCTTAAACGAGGAATGC	
	122	qP_rRNA-18S2_rv	GAATCAGTTCAGTCCCGGATAG	
	123	qP_rRNA-5.8S_fw	TACTTACCACGAATTGCAGACG	
	124	qP_rRNA-5.8S_rv	ACCAGACGTACCAACTGGAG	
	127	qP_rRNA-26S2_fw	GATGTCGGCTCTTCCTATCATT	
	128	qP_rRNA-26S2_rv	CCAGCTCACGTTCCCTATTAGT	
competimers	155	qP_rRNA-18S2_fwC	TATTGCCCTTAAACGAGGAATGdC	3'-deoxy C
	156	qP_rRNA-18S2_rvC	GAATCAGTTCAGTCCCGGATAdG	3'-deoxy G
	157	qP_rRNA-5.8S_fwC	TACTTACCACGAATTGCAGACdG	3'-deoxy G
	158	qP_rRNA-5.8S_rvC	ACCAGACGTACCAACTGGAdG	3'-deoxy G
	159	qP_rRNA-26S2_fwC	GATGTCGGCTCTTCCTATCATdT	3'-deoxy T
	160	qP_rRNA-26S2_rvC	CCAGCTCACGTTCCCTATTAGdT	3'-deoxy T
**qRT-PCR of Pol II transcripts**				
ced-3	222	qP_ced-3_fw	CATTCATCGGATCGACACAA	
	223	qP_ced-3_rv	TGAAGAGTTGGCGGATGAA	
ced-4	224	qP_ced-4_fw	GATGTGCTTAATAAAACAATCGAACT	
	225	qP_ced-4_rv	ATCCTCGACTTTCCAATTTGTT	
ced-9	149	qP_T07C4.8_fw1	CGGAGAGTCAATTGATGGAAA	
	150	qP_T07C4.8_rv1	GATTCGGTGCGTGAAATAGTC	
cep-1	175	qP_cep-1(all)_fw	CGATGAAGAGAAGTCGCTGT	
	176	qP_cep-1(all)_rv	ATCTGGGAACTTTTGCTTCG	
egl-1		egl-1 for	CAGGACTTCTCCTCGTGTGAAGATTC	
		egl-1 rev	CGAAGTCATCGCACATTGCTGCTA	
ced-13	169	qP_ced-13_fw	CTTTCTCCCGCTGTTGTCTATT	
	170	qP_ced-13_rv	GGTGTTTGAGTTGCAAGCATTA	
	480	qp_ced-13_fw2	AGTTCAGAGTCAAACTCGTC	
	481	qp_ced-13_rv2	AGCTCCCTGTTTATCacTTC	
controls	161	qP_rpl-29_fw	CAAGTCCAAGAACCACACCA	
	162	qP_rpl-29_rv	TCCCTTCATGGAGAGGAAGA	
	163	qP_rps-4_fw	GGAGCATCTGACATCATCCA	
	164	qP_rps-4_rv	GACGAAGACGTTGGAGATACG	
		ce_PGK1_fw	GCGATATTTATGTCAATGATGCTTTC	
		ce_PGK1_rv	TGAGTGCTCGACTCCAcCCA	
	230	qP_cdc-42_fw	CTGCTGGACAGGAAGATTACG	
	231	qP_cdc-42_rv	CTCGGACATTCTCGAATGAAG	
	232	qP_pmp-3_fw	GTTCCCGTGTTCATCACTCAT	
	233	qP_pmp-3_rv	ACACCGTCGAGAAGCTGTAGA	
	234	qP_Y45F10D.4_fw	AAGCGTCGGAACAGGAATC	
	235	qP_Y45F10D.4_rv	GATTTTTCCGTTATCGTCGACT	
