# Supplementary material for: Ribosome Synthesis and MAPK Activity Modulate Ionizing Radiation-Induced Germ Cell Apoptosis in Caenorhabditis elegans
Source: PLoS Genet. 2013 Nov 21;9(11):e1003943. doi: 10.1371/journal.pgen.1003943 (PMC3836707; doi:10.1371/journal.pgen.1003943)
Supplement: Text S1 — Supplementary Results, Supplementary Methods, and Supplementary References. The citations referred to in the Supplementary Figure legends are all listed as Supplementary References in Text S1. (PDF) [file pgen.1003943.s023.pdf]

Ribosome synthesis and MAPK activity modulate ionizing radiation-induced germ cell apoptosis in *Caenorhabditis elegans*

Running Head: Ribosome synthesis and apoptosis in *C. elegans*

Ralf Eberhard, Lilli Stergiou, E. Randal Hofmann, Jen Hofmann, Simon Haenni, Youjin Teo, André Furger, and Michael O. Hengartner

Supplementary Text S1

Supplementary Results

Supplementary Methods

Supplementary References

October 2013

Supplementary Results

Local bias for germ cell apoptosis levels in *rpoa-2(ok1970)/hT2* animals

Heterozygous animals in which the lethal deletion in *ok1970* (Fig. S2A) was genetically maintained by the *hT2* balancer exhibited a peculiar apoptotic phenotype: IR-induced apoptosis was abolished completely, however, baseline apoptosis was increased in comparison to wild type (Fig. S1D). Surprisingly, this was fully on the account of anterior gonads (7 corpses on average, posterior gonads only 2 corpses). Such a strong bias has not been reported before and we have not observed it for other strains. (Worms grown on RNAi bacteria have a tendency for higher corpse numbers in the posterior gonad (our observations)). To exclude that this effect was merely due to the balancer, we looked at other mutant strains balanced by *hT2*; those strains showed an only slightly higher number of corpses in the anterior gonad (not shown), attributing the main effect to *rpoa-2(ok1970/+)*. The cause of this unusual phenotype is at this point unclear.

The Gogo phenotype is dependent on CEP-1/p53 but not on apoptosis execution

We observed a distinct germ line phenotype in a fraction of *rpoa-2(op259)* mutant gonads at 20°C: oocytes occurred ectopically in the distal arm, forming islets within apparently normal early pachytene nuclei in the distal gonad (Fig. S7A). Sometimes, they reached the diameter of the gonad tube and completely separated the smaller pachytene cells in the distal gonad into two or more regions. Because of the resulting succession of (pachytene stage) Germ cells – Oocytes – Germ cells – Oocytes along the gonad, we named this defect 'Gogo' phenotype (schematic in Fig. S7B). DAPI staining confirmed that the distal oocytes were adjoined by meiotic pachytene cells distally and proximally (Fig. S7A) and that they were rather a result of precocious oocyte maturation than of proliferation proximally to gametes as is the case with the Pro phenotype (Fig. S6B). Interestingly, *rpoa-2(op259)* worms fed on HT115 RNAi bacteria were more likely to exhibit distal oocytes at 20°C than worms fed on the standard OP50 food. Further, IR treatment potently enhanced the penetrance of the

phenotype: in *rpoa-2(op259)* animals on HT115 bacteria, around 20 % of gonads showed a distal oogenesis phenotype by 48 hours after irradiation (Fig. S7C). Surprisingly, the sites of ectopic oogenesis were often accompanied by a high number of corpses. Again, irradiation significantly increased cell death at these sites, to corpse numbers that by far outrange orthotopic irradiation-induced apoptosis of wild type worms (semi-quantitative, not shown). Given the stimulatory effect of IR, it was conceivable that these cell deaths and possibly also ectopic oogenesis were mediated by CEP-1/p53. Indeed, loss of *cep-1* function significantly reduced the fraction of gonads developing distal oocytes and associated corpses. Blocking apoptosis execution by *ced-3(RNAi)* also abolished cell corpses, but did not prevent the distal oocytes. This indicates that formation of the latter does not depend on germ cell death, and that CEP-1 might regulate germ cell progression through other modes than induction of apoptosis. These findings add a novel aspect to CEP-1 function besides its roles in DNA damage-induced germ cell apoptosis [14] and in germ cell proliferation [15]. It is conceivable that alterations in Ras/MAPK pathway activity in the germ line of *rpoa-2(op259)* animals also contribute to the Gogo phenotype, given the functions in germ cell progression and oocyte growth, and that the disorder reflects combined effects of CEP-1/p53 and MAP kinase misregulation in the mutant.

CEP-1-positive nucleolar substructures of meiotic pachytene cells are enlarged in *rpoa-2(op259)*

In *rpoa-2(op259)* mutants, we noticed an increase in the size of nucleolar substructures in the large nuclei of germ cells (Fig. 2A and Fig. S14B) and intestinal cells (Fig. S8A). They appeared as mostly central dots by DIC microscopy. CEP-1::GFP precisely colocalised with them (Fig. S8B). In contrast, YFP::RPOA-2 – and thus presumably the active RNA polymerase I complex – spared them. Similar structures have been reported in mammalian cells that are distinct from the sites of ribosome production [16] and that contain p53 and other stress response factors [17,18]. In our electron microscopy images of wild-type worms, some of the meiotic germ cell nucleoli had central areas of lower density, which on occasional sections appeared confluent with the nucleoplasm (not shown). We found various fluorescent nuclear factors besides CEP-1::GFP to also localise into these dots, which we therefore think are in communication with the nucleoplasm.

5-fluorouridine incorporation assay detects rRNA synthesis *in situ*

To assess rRNA transcription specifically in the gonad, we adapted an *in vivo* assay from a cell culture system that uses the nucleotide analogue 5-fluorouridine (5-FU) [19]. It is efficiently incorporated into nascent RNA by the RNA polymerases and can be visualised with an anti-BrdU antibody. Since the absolute majority of transcriptional activity in a cell is committed to rRNA synthesis, a short pulse will primarily highlight transcription of ribosomal RNA. We incubated dissected gonads with 5-FU before fixation and staining (see Supplementary Methods). The signal predominantly localised to the nucleoli, confirming that 5-FU could be absorbed by the germ cells, and that it could be detected most strongly at the sites of early rRNA synthesis (Fig. S9A). Comparison of the staining pattern and signal intensity between wild type and *rpoa-2(op259)* mutant worms could not conclusively demonstrate or exclude a difference between the two (Fig. S9B).

Nucleolar disruption is possibly a mechanistical step in DNA damage-induced apoptosis [20,21]. How readily and dramatically RNA pol I transcription can be affected by genotoxic treatment has been demonstrated with quantitative *in situ* analysis of RNA pol I kinetics in mammalian cells: irradiation caused a rapid but temporary decline of RNA pol I assembly and rRNA transcription, dependently on the damage signalling PI(3)K kinase ATM [19]. The 5-FU assay adapted to the germ line of *C. elegans* did not show such decline of rRNA synthesis after IR or UV-C irradiation.

The 26S-short is a non-polyadenylated truncation of the 26S rRNA

When separating total worm RNA in denaturing agarose gels and staining with EtBr (Fig. 2E), we noticed a distinct band between the outstanding 26S and 18S rRNA bands that was more prominent in *rpoa-2(op259)* samples than in wild-type extracts. Given its size and its relatively high abundance compared to all other RNA, the band likely represented a pre-rRNA processing intermediate or a shortened 26S rRNA. Truncated versions of 26S rRNA that are polyadenylated and supposedly targeted for degradation have been described in other species [22,23]. We used DIG-labelled probes to rRNA (Fig. S11A) to characterise the band in *C. elegans*. The 18S probe, the 26S-2 probe that covers the

3' end of 26S rRNA, and the its2 probe did not hybridise to a band of the according size; only 26S-1, the 5' probe for 26S rRNA, gave a distinct band at the expected position, which was again more pronounced in *rpoa-2(op259)* than in wild-type samples (Fig. S11B). Using short oligo probes, we confined the 3' end of this supposedly truncated 26S rRNA within 600 nucleotides (Fig. S11C). Circularisation of the RNA, reverse transcription, and PCR amplification over the presumptive, ligated ends (see Supplementary Methods) allowed us to precisely determine the sequence at the 5' and 3' ends of the short 26S rRNA (26S-short). The 5' end coincided with the annotated start site of the 26S ribosomal RNA. Surprisingly, the 3' end seemed the result of a clear cut of the 26S rRNA sequence without signs of polyadenylation (Fig. S11D). One additional U in about half the product might be attributed to either end of 26S-short (both ends are flanked by U in the pre-RNA sequence). With a second approach – adapter ligation – we could confirm the above findings for the ends of 26S-short, and the identity of the internal sequence with the 26S rRNA consensus. In this analysis, the majority of 26S-short proved to have an U preceding the annotated start site of the 26S rRNA [7]. There were no differences in the nucleotide sequence of the 26S-short between wild-type and *rpoa-2(op259)* mutant worms.

A band parallels the increase of 26S-short in *rpoa-2(op259)* that apparently corresponds to the 3' counterpart (Fig. S11B). 26S-short is thus possibly a cleavage product of full-length 26S rRNA. Such degradation might happen with mature rRNA in ribosomes, or it could happen during transcription/processing of the pre-rRNA. In an analysis of nuclei isolated from adult *C. elegans* [24], we found a clear increase of the 26S-short/26S rRNA ratio in nuclear RNA compared to RNA extracted from whole worms (not shown). Theoretically, 26S degradation could represent a mechanism to adjust 26S and 18S rRNA levels, before export of the large ribosomal subunit; or the 26S rRNA might be degraded primarily, due to a processing defect for instance. Alternatively, the 26S fragment itself could serve a specific purpose in the nucleoli or nuclei. At least, its relative abundance in total RNA is considerable even in wild type and relatively stable within each worm strain.

26S-short does not depend on apoptosis execution

Interestingly, bands corresponding to truncated 28S rRNA were observed in the context of apoptosis in mammalian cells [25–28] or of viral infection [29]. To determine whether the 26S-short band in *C. elegans* was a product of apoptotic degradation of nucleic acids, we checked the RNA profile of *ced-3(n717)* mutants, where apoptotic decay is mostly supressed due to missing caspase activity. *ced-3(n717)* mutants also had the 26S-short band, at a similar intensity as wild-type worms, and *ced-3(n717)* could not suppress the increase in *rpoa-2(op259)* (Fig. 2F). Thus, the truncated 26S rRNA in whole worm extracts is unlikely to be a product of apoptosis. The multi-exonuclease exosome has important functions in processing of ribosomal RNA [e.g., 30]. We examined an available mutant of *crn-3*, the homolog of the exosome component PM/Scl-100 in mammals (Rrp6 in yeast); as a cell death-related nuclease, it is also required for normal execution of apoptosis [31]. The relative intensity of the 26S-short band in *crn-3(ok2269)* was not clearly different from wild type, and the increase in *rpoa-2(op259)* was not abolished by mutation of this exosome component (Fig. 2F).

Interfering with rRNA processing factors and ribosomal proteins disturbs IR-induced apoptosis

We tested the effect of knocking down various ribosomal RNA processing factors by RNAi: U3 spliceosome subunits to assess early steps of 18S rRNA and thus small subunit (SSU) biogenesis, and early and later factors of large subunit (LSU) maturation (Table S3). We titrated RNAi conditions such that possible effects would become visible without the germ line having decayed already. Despite our efforts, most treatments had strong effects on germ line integrity, on fertility, and on viability. IR-induced germ cell apoptosis was reduced in most cases; the effect was slightly less pronounced in processing factors of the small subunit 18S rRNA and of later steps of ribosome synthesis (Table S3). To assess whether disturbing ribosome synthesis by reducing individual ribosomal proteins would also impact on germ cell death, we arbitrarily picked *rps-1*, *rps-2* and *rpl-1*, *rpl-2* as representatives of the small and large subunits, respectively (the numbering of the ribosomal proteins in metazoa is not systematic as to molecular weight or functional groups). The effect on germ line

appearance was very pronounced; germ cell contours became blurred and the phase contrasts in DIC changed around nucleoli and nuclei. The number of apoptotic corpses was reduced to very low levels in all four cases (Table S3).

IR-induced apoptosis is sensitive to translation inhibition by cycloheximide

The bacterial toxin cycloheximide (CHX) blocks translation in eukaryotic organisms by directly interfering with translation elongation, and it has therefore been widely used in cell culture studies. We treated wild-type worms with increasing concentrations of cycloheximide (starting exposure at a young adult stage) and irradiated them (see Supplementary Methods). At 350 µg/ml, most of the IR response seen in control treated animals was abolished, whereas baseline apoptosis was still present (Fig. S12A). Lower doses that did only marginally or not impair development had little to no effect on apoptosis. At doses higher than 500 µg/ml, only few gonads could be assessed due to strong adverse effects of the drug on the organism; for those scored, IR-induced apoptosis was completely abolished and also the corpse number in non-irradiated control animals was reduced. Thus, irradiation-induced apoptosis is effectively blocked by chemical inhibition of translation. However, a significant effect on IR-induced apoptosis is only reached with CHX doses that also lead to massive impairment of animal health (Fig. S12A).

We used the engulfment defective background *ced-6(n1813)* to increase the 'resolution' of the number of apoptotic corpses at baseline levels, that is, in the absence of exogenous DNA damage. Dying germ cells are inefficiently removed in this mutant and accumulate in the gonad throughout adulthood. We transferred the animals to CHX plates as young adults, and scored the number of accumulated corpses 16 hours later. Consistent with the previous experiments, 500 µg/ml CHX did not strongly block baseline apoptosis; and the corpse number in non-irradiated *ced-6(n1813)* only weakly decreased with higher doses of CHX (Fig. S12B). This weak, gradual reduction in the number of accumulated corpses was not due to a CEP-1/p53-dependent effect, since it could also be observed in *cep-1(gk138); ced-6(n1813)*. In *rpoa-2(op259); ced-6(n1813)*, the number of corpses was reduced to

half the control levels already at 250 µg/ml CHX; and at 500 µg/ml, it reached a mere 40 %. The strong response of baseline apoptosis is in agreement with the notion that in *rpoa-2(op259)* mutants, unlike in wild type, most of the baseline germ cell apoptosis is dependent on CEP-1 and thus on *de novo* protein synthesis. This does, however, not exclude that other mechanisms make *rpoa-2(op259)* more sensitive to CHX treatment. For instance, *rpoa-2(op259)* mutants might be particularly sensitive to chemical inhibition of translation due to a reduced capacity of the translational apparatus (numerically or structurally aberrant ribosomes).

The core apoptotic factors in *rpoa-2(op259)* mutants

Given our finding that *rpoa-2(op259)* leads to modulation of apoptosis downstream of CEP-1-induced upregulation of EGL-1, we considered its effect on the core apoptotic factors CED-9/Bcl-2, CED-4/Apaf-1, or CED-3/effector caspase (schematic in Fig. S13A). We first looked at mRNA levels. Relevant changes at the transcript level of these core factors have been found for several mutants with defective germ line apoptosis: in the Rb complex mutants *lin-35* or *dpl-1*, decreased germ cell death correlates with derepression of CED-9 transcription in the germ line, or reduced transcriptional activation of CED-4 and CED-3, respectively [32]; the pro-survival factors PAX-2 and EGL-38 limit germ cell apoptosis probably through upregulation of CED-9 transcription [33]. We compared CED-9, CED-4, and CED-3 transcript levels between *rpoa-2(op259)* and wild-type animals. CED-3 and CED-4 were very stable between the two, excluding a strong effect of *rpoa-2(op259)* on transcription of these core pro-apoptotic factors (Fig. S14A). CED-9 transcript levels were moderately but consistently decreased in *rpoa-2(op259)* whole worm RNA extracts (*t*-test, p=0.012), which disfavours the hypothesis that *rpoa-2(op259)* blocks apoptosis by upregulating CED-9 expression.

We used the CED-4::GFP (*opIs219*) reporter line [34] to assess for possible alterations of CED-4 at the level of protein expression or subcellular localisation in the apoptotic region. We detected perinuclear fluorescence in all germ cells of the meiotic pachytene region, similar to what has been described for immuno-detection of CED-4 [35,36]. There was no obvious difference in the level and

pattern of CED-4::GFP between the wild-type and *rpoa-2(op259)* background (Fig. S14C). These observations speak against a reduced expression of pro-apoptotic CED-4 as the reason for the low germ cell apoptosis levels in *rpoa-2(op259)* animals.

Due to the lack of adequate reporters, we used a genetic approach to the potential role of the pro-survival protein CED-9. Loss of *ced-9* function is lethal due to excessive cell death in early development. The *ced-9(n1653)* allele however, is temperature sensitive [37], exhibiting massive apoptosis at 25°C but showing moderately increased cell death at 20°C. A reduction of *ced-3* function by the *ced-3(n2438)* allele can lower excessive death in this mutant (Fig. S15A), consistent with the action of *ced-3* downstream of *ced-9*. We tested for epistasis of *rpoa-2(op259)* and *ced-9(n1653)*. To our surprise, apoptosis levels in the *rpoa-2(op259); ced-9(n1653)* double mutant were not only restored to the high levels of *ced-9(n1653)*, but apoptosis was even more strongly enhanced (Fig. S15A). At 20°C, young adult double mutants rapidly started accumulating a large number of cell corpses; eventually, all germ cells decayed so that none progressed to oocytes and the animals became sterile. Cell death and sterility could be suppressed by loss of *ced-3* function, confirming the apoptotic nature of the process (not shown).

We speculated that the now increased cell death levels might be provoked by the increased EGL-1 levels we had detected in *rpoa-2(op259)* animals. Indeed, the presumptive *egl-1(n1084 n3082)* loss-of-function allele potently suppressed excessive apoptosis in *rpoa-2(op259); ced-9(n1653)* (Fig. S15B). These findings also show that the *ced-9(n1653)* product CED-9(Y149N) does not detach the core apoptotic machinery from upstream signals and is very responsive to EGL-1, and second, *rpoa-2(op259)* does have not only increased EGL-1 transcript levels but also active EGL-1 protein. That *rpoa-2(op259); ced-9(n1653)* animals have more apoptosis than the *ced-9* single mutants indicates that the apoptosis execution machinery is present and fully functional in *rpoa-2(op259)*.

To further characterise the critical role of CED-9 in *rpoa-2(op259)*, we knocked down its expression by RNAi. In wild-type worms, *ced-9(RNAi)* treatment led to a strong increase in germ cell apoptosis;

yet fertility in the generation tested was maintained, indicating that the knock-down effect was that of a reduction and not a full loss of *ced-9* function at this moment. In *rpoa-2(op259)* mutants – contrasting with the effect of the *ced-9(n1653)* mutation – *ced-9(RNAi)* did not increase the baseline number of corpses (Fig. S15C). However, knockdown of *ced-9* restored significant IR sensitivity in *rpoa-2(op259)*, a response that was clearly dependent on CEP-1 (Fig. S15C). These observations point to a functional difference between a reduction of wild type CED-9 levels and the CED-9(Y149N) point mutant. Further work will be required to elucidate the nature of this difference.

Taken together, our molecular and genetic data suggest that *rpoa-2(op259)* reduces apoptosis by a molecular mechanism impinging at the level of CED-9. Our observations also argue against the hypothesis that the block in apoptosis in *rpoa-2(op259)* is solely due to the slow proliferation and differentiation rates observed in this mutant.

Supplementary Methods

DAPI staining of whole worms

Staged worms were washed in M9 buffer, fixed (3 % PFA for 30 min at 4°C followed by 100 % methanol for 10 min at -20°C), stained with DAPI 200 ng/ml in PBS/T for 10 min and mounted in Prolong Gold (Invitrogen) (>3 washes with PBS/Tween 0.1 % between all steps).

5-FU incorporation

Germ lines were extruded from adult animals by needle dissection – thus overcoming the impenetrable cuticle – and incubated with 5-FU (8 mM in M9 buffer) for 15 min before fixation in 3 % PFA for 30 min at 4°C, freeze cracking on poly-lysine coated slides, fixation in 100 % methanol for 10 min at -20°C, staining (blocking in 5 % BSA in PBS/T for 2 hours at RT; 1° anti-BrdU (Sigma BU-33) 1:1000 in blocking buffer o/n at 4°C; 2° rabbit anti-mouse IgG (Alexa Fluor) 1:500 in blocking buffer for 1 hour at RT; DAPI 200 ng/ml in PBS/T for 10 min; >3 washes with PBS/Tween 0.1 % between all steps), and mounting in Prolong Gold (Invitrogen).

Hybridisation with DIG-labelled probes

Specific 300 nt long rDNA segments were cloned into pGEM-T easy (Promega), and digoxigenin (DIG)-labelled antisense RNA probes were generated by SP6 transcription from linearised vectors (Roche, DIG-Northern kit). DIG-oligo probes were generated by 3'-end labelling of specific DNA oligonucleotides (Roche). Total RNA extracted from adult worms (200 to 800 ng) were separated on denaturing agarose gels and blotted to nylon membranes by passive transfer over several hours followed by UV-crosslinking. DIG-labelled RNA probes were hybridised at 68°C, DNA oligos at 55°C; detection was with alkaline phosphatase-coupled anti-DIG antibody and the chemoluminescent substrate CPDstar. Membrane stripping often left residual signal in the subsequent detection, which was compensated by stepping from probes with weak signals to those with strong signals.

26S-short RNA end-detection

RNA circularisation and reverse transcription were performed according to the principle presented in [38]. Total RNA was separated on agarose gels and the band between the 26S and 18S rRNA was excised, RNA was extracted and circularised with RNA ligase. This was followed by reverse transcription with a primer annealing close to the 5' end of 26S rRNA (primer_076). In the case of RNA circles, reverse transcription should extend across the former 5' end into the adjoining 3' end.

The cDNA served as template for a PCR reaction with a pair of primers that should preferentially amplify the truncated 26S rRNA: primer_076 close to the 5' end, and a forward primer close to the presumptive 3' end of the truncated 26S rRNA (primer_191). With short enough an extension time during the PCR reaction, specifically the reverse transcripts from circularised truncated 26S rRNA and not from contaminating full length 26S rRNA should be amplified. The distinct PCR product of the expected size (<700 bp) was sequenced. If the short 26S rRNA band represented a precisely truncated but variably polyadenylated version of 26S rRNA, one would expect a single sequence down to the 3' truncation site followed by some As (or Ts) that would flare into a sequence mix. However, Sanger sequencing (primer_076 or primer_191) resulted in unique sequence reads that abruptly changed into an overlap of two sequences; the two overlapping sequences could be resolved as the same sequence shifted by 1 nt. The 3' ends thus stemmed from a neat cut of the 26S rRNA sequence and did not carry a poly-A tail. The additional U in about half the product could stem from alternative cutting at either end. In a second approach, adapter RNA oligonucleotides were ligated to the ends of gel separated transcripts: RNA_SRA5Adpt at the free 5' ends and consecutively RNA_SRA4Adpt (P5-phosphorylated 5' end and idT-modified 3' end) at the 3' ends. Reverse transcription with rc_RT_GX1 complementary to the RNA_SRA4Adpt 3' adapter or with primer_076 to focus on the 5' terminus, and subsequent sequencing of specific PCR products (rc_PCR_fw and primer_076, primers_125/214, primers_199/218, primers_201/060, primer_191 and rc_RT_GX1) confirmed the 3' end of 26S-short as a cut in the sequence 5'-CCAUUUUAUGG-3' (position 2830 of 26S rRNA, position 6372 in rDNA reference sequence), either after the second or the third U, and an U preceding the annotated start site of the 26S rRNA [7]. It also confirmed the consensus 26S rRNA sequence, indicating that the 26S-short rRNA is a truncated 26S rRNA version and therefore not likely to result from transcription of another locus than the rDNA repeats.

Cycloheximide assays

Cycloheximide (CHX) was dissolved in 12.5 % v/v ethanol and serially diluted. NGM agar plates seeded with OP50 were UV-irradiated for 10 min in a Stratalinker to kill the bacteria (according to [39]), and 250 µl of the CHX solution were evenly spread on the plate 6 hours before use. Final concentrations were calculated as µg CHX per ml agar. Young adult worms were transferred from normal plates to the drug-containing plates 6 hours before irradiation. Scoring of apoptotic corpses was at the time points indicated.

LIP-1 immunofluorescence

Antibodies used for staining were anti-LIP-1 [40] (pre-absorbed on acetone powder of *lip-1(zh15)* deletion mutants, 1:100) and a protein-independent control antibody to dsDNA (Abcam HYB331-01, 1:500). Secondary antibodies: Alexa Fluor goat anti-rat IgG (anti-LIP-1), goat anti-mouse IgG (anti-dsDNA), both 1:500. Blocking buffer: 10 % BSA in antibody buffer according to [41]. Gonads were extruded by dissecting adult hermaphrodites; samples were fixed with 3 % PFA for 30 min at 4°C, freeze cracked, fixed with 100 % methanol for 10 min at -20°C, stained (blocking for 1 hour at RT; 1° antibodies in blocking buffer o/n at 4°C; 2° antibodies in blocking buffer for 1 hour at RT; >3 washes with PBS/Tween 0.1 % between all steps), and mounted on poly-lysine coated slides.

Supplementary References

1. Sijen T, Fleenor J, Simmer F, Thijssen K, Parrish S, et al. (2001) On the role of RNA amplification in dsRNA-triggered gene silencing. Cell 107: 465–476.
2. Ketting RF, Haverkamp TH, van Luenen HG, Plasterk RH (1999) Mut-7 of C. elegans, required for transposon silencing and RNA interference, is a homolog of Werner syndrome helicase and RNaseD. Cell 99: 133–141.
3. Obenauer JC, Cantley LC, Yaffe MB (2003) Scansite 2.0: Proteome-wide prediction of cell signaling interactions using short sequence motifs. Nucleic Acids Res 31: 3635–3641.
4. Sulston JE, Brenner S (1974) The DNA of Caenorhabditis elegans. Genetics 77: 95–104.
5. Files J, Hirsh D (1981) Ribosomal DNA of Caenorhabditis elegans. J Mol Biol 149: 223–240.
6. Albertson DG (1984) Localization of the ribosomal genes in Caenorhabditis elegans chromosomes by in situ hybridization using biotin-labeled probes. EMBO J 3: 1227–1234.
7. Ellis R, Sulston J, Coulson A (1986) The rDNA of C. elegans: sequence and structure. Nucleic Acids Res 14: 2345–2364.
8. Taylor D, Devkota B, Huang A, Topf M, Narayanan E, et al. (2009) Comprehensive molecular structure of the eukaryotic ribosome. Structure 17: 1591–1604. doi:10.1016/j.str.2009.09.015.
9. Qi S, Pang Y, Hu Q, Liu Q, Li H, et al. (2010) Crystal structure of the Caenorhabditis elegans apoptosome reveals an octameric assembly of CED-4. Cell 141: 446–457. doi:10.1016/j.cell.2010.03.017.
10. Ward J, Muzzini D, Petalcorin M, Martinez-Perez E, Martin J, et al. (2010) Overlapping Mechanisms Promote Postsynaptic RAD-51 Filament Disassembly during Meiotic Double-Strand Break Repair. Mol Cell 37: 259–272. doi:10.1016/j.molcel.2009.12.026.
11. Deng X, Hofmann ER, Villanueva A, Hobert O, Capodieci P, et al. (2004) Caenorhabditis elegans ABL-1 antagonizes p53-mediated germline apoptosis after ionizing irradiation. Nat Genet 36: 906–912. doi:10.1038/ng1396.
12. Werner M, Thuriaux P, Soutourina J (2009) Structure-function analysis of RNA polymerases I and III. Curr Opin Struct Biol 19: 740–745. doi:10.1016/j.sbi.2009.10.005.
13. Kuhn C-D, Geiger SR, Baumli S, Gartmann M, Gerber J, et al. (2007) Functional architecture of RNA polymerase I. Cell 131: 1260–1272. doi:10.1016/j.cell.2007.10.051.
14. Schumacher B, Hofmann K, Boulton S, Gartner A (2001) The C. elegans homolog of the p53 tumor suppressor is required for DNA damage-induced apoptosis. Curr Biol 11: 1722–1727.
15. Waters K, Yang A, Reinke V (2010) Genome-wide analysis of germ cell proliferation in C.elegans identifies VRK-1 as a key regulator of CEP-1/p53. Developmental Biology 344: 1011–1025. doi:10.1016/j.ydbio.2010.06.022.
16. Krüger T, Scheer U (2010) p53 localizes to intranucleolar regions distinct from the ribosome production compartments. J Cell Sci 123: 1203–1208. doi:10.1242/jcs.062398.
17. Latonen L, Moore HM, Bai B, Jäämaa S, Laiho M (2011) Proteasome inhibitors induce nucleolar aggregation of proteasome target proteins and polyadenylated RNA by altering ubiquitin availability. Oncogene 30: 790–805. doi:10.1038/onc.2010.469.
18. Karni-Schmidt O, Zupnick A, Castillo M, Ahmed A, Matos T, et al. (2008) p53 is localized to a sub-nucleolar compartment after proteasomal inhibition in an energy-dependent manner. J Cell Sci 121: 4098–4105. doi:10.1242/jcs.030098.
19. Kruhlak M, Crouch E, Orlov M, Montaño C, Gorski S, et al. (2007) The ATM repair pathway inhibits RNA polymerase I transcription in response to chromosome breaks. Nature 447: 730–734. doi:10.1038/nature05842.
20. Rubbi CP, Milner J (2003) Disruption of the nucleolus mediates stabilization of p53 in response to DNA damage and other stresses. EMBO J 22: 6068–6077.
21. Olson M (2004) Sensing Cellular Stress: Another New Function for the Nucleolus? Science's STKE 2004: pe10. doi:10.1126/stke.2242004pe10.
22. Shcherbik N, Wang M, Lapik YR, Srivastava L, Pestov DG (2010) Polyadenylation and degradation of incomplete RNA polymerase I transcripts in mammalian cells. EMBO Rep 11: 106–111. doi:10.1038/embor.2009.271.

23. Kuai L, Fang F, Butler J, Sherman F (2004) Polyadenylation of rRNA in Saccharomyces cerevisiae. P Natl Acad Sci Usa 101: 8581–8586. doi:10.1073/pnas.0402888101.
24. Haenni S, Ji Z, Hoque M, Rust N, Sharpe H, et al. (2012) Analysis of C. elegans intestinal gene expression and polyadenylation by fluorescence-activated nuclei sorting and 3'-end-seq. Nucleic Acids Res 40: 6304–6318. doi:10.1093/nar/gks282.
25. Nadano D, Sato T (2000) Caspase-3-dependent and -independent degradation of 28 S ribosomal RNA may be involved in the inhibition of protein synthesis during apoptosis initiated by death receptor engagement. J Biol Chem 275: 13967–13973.
26. Degen W, Pruijn G, Raats J, van Venrooij W (2000) Caspase-dependent cleavage of nucleic acids. Cell Death Differ 7: 616–627. doi:10.1038/sj.cdd.4400672.
27. Samali A, Gilje B, Doskeland S, Cotter T, Houge G (1997) The ability to cleave 28S ribosomal RNA during apoptosis is a cell-type dependent trait unrelated to DNA fragmentation. Cell Death Differ 4: 289–293.
28. Houge G, Robaye B, Eikhom T, Golstein J, Mellgren G, et al. (1995) Fine mapping of 28S rRNA sites specifically cleaved in cells undergoing apoptosis. Mol Cell Biol 15: 2051–2062.
29. Banerjee S, An S, Zhou A, Silverman R, Makino S (2000) RNase L-independent specific 28S rRNA cleavage in murine coronavirus-infected cells. J Virol 74: 8793–8802.
30. Allmang C, Mitchell P, Petfalski E, Tollervey D (2000) Degradation of ribosomal RNA precursors by the exosome. Nucleic Acids Res 28: 1684–1691.
31. Parrish J, Xue D (2003) Functional genomic analysis of apoptotic DNA degradation in C. elegans. Mol Cell 11: 987–996.
32. Schertel C, Conradt B (2007) C. elegans orthologs of components of the RB tumor suppressor complex have distinct pro-apoptotic functions. Development 134: 3691–3701. doi:10.1242/dev.004606.
33. Park D, Jia H, Rajakumar V, Chamberlin HM (2006) Pax2/5/8 proteins promote cell survival in C. elegans. Development 133: 4193–4202. doi:10.1242/dev.02614.
34. Zermati Y, Mouhamad S, Stergiou L, Besse B, Galluzzi L, et al. (2007) Nonapoptotic role for Apaf-1 in the DNA damage checkpoint. Mol Cell 28: 624–637. doi:10.1016/j.molcel.2007.09.030.
35. Pourkarimi E, Greiss S, Gartner A (2011) Evidence that CED-9/Bcl2 and CED-4/Apaf-1 localization is not consistent with the current model for C. elegans apoptosis induction. Cell Death Differ 19: 406–415. doi:10.1038/cdd.2011.104.
36. Greiss S, Hall J, Ahmed S, Gartner A (2008) C. elegans SIR-2.1 translocation is linked to a proapoptotic pathway parallel to cep-1/p53 during DNA damage-induced apoptosis. Genes Dev 22: 2831–2842. doi:PMC2569882.
37. Hengartner MO, Ellis RE, Horvitz HR (1992) Caenorhabditis elegans gene ced-9 protects cells from programmed cell death. Nature 356: 494–499. doi:10.1038/356494a0.
38. West S, Gromak N, Norbury C, Proudfoot N (2006) Adenylation and exosome-mediated degradation of cotranscriptionally cleaved pre-messenger RNA in human cells. Mol Cell 21: 437–443. doi:10.1016/j.molcel.2005.12.008.
39. Kourtis N, Tavernarakis N (2009) Cell-specific monitoring of protein synthesis in vivo. PLoS ONE 4: e4547. doi:10.1371/journal.pone.0004547.
40. Lee M-H, Hook B, Lamont LB, Wickens M, Kimble J (2006) LIP-1 phosphatase controls the extent of germline proliferation in Caenorhabditis elegans. EMBO J 25: 88–96. doi:10.1038/sj.emboj.7600901.
41. Duerr JS (2006) Immunohistochemistry. Duerr JS (2006) Immunohistochemistry. WormBook, ed. The C. elegans Research Community, http://www.wormbook.org. doi/10.1895/wormbook.1.105.1.
